# Formative research in Ethiopia and Bangladesh: strengthening household survey measures of maternal micronutrient supplementation in low- and middle-income countries

**DOI:** 10.7189/jogh.16.04159

**Published:** 2026-07-03

**Authors:** Shelley Walton, Ellina Wood, Hanna Y Berhane, Firehiwot Workeneh, Fiker Ferdaweke, Sk Masum Billah, Sharif Uddin Lotus, Melinda Munos, Sunny Kim, Swetha Manohar, Phuong Nguyen, Syed Shehryar Azeem, Rebecca Heidkamp

**Affiliations:** 1 International Health Department, Johns Hopkins Bloomberg School of Public Health, Baltimore, Maryland, USA; 2 Department of Nutrition and Behavioral Sciences, Addis Continental Institute of Public Health, Addis Ababa, Ethiopia; 3 Sydney School of Public Health, The University of Sydney, Sydney, Australia; 4 International Centre for Diarrhoeal Disease Research, Maternal and Child Health Division, Dhaka, Bangladesh; 5 International Food Policy Research Institute, Washington, DC, USA; 6 International Food Policy Research Institute, Hanoi, Vietnam

## Abstract

**Background:**

As countries transition from implementing iron folic acid (IFA) to multiple micronutrient supplements (MMS) for pregnant women, they need accurate data on who is being reached with these interventions. The aim of our formative research in Ethiopia and Bangladesh was to design updated household survey questions, response options, and visual aids to measure coverage of prenatal micronutrient supplements.

**Methods:**

In phase I, we landscaped health providers and retailers to identify prenatal micronutrient products available in each country and interviewed currently pregnant women (CPW) and recently delivered women (RDW) about how they differentiated among micronutrient products. In phase II, we used these findings to draft survey questions and visual aids. In phase III, we iteratively tested and refined the draft tools through cognitive interviews with women.

**Results:**

We had 73 participants in Ethiopia and 132 in Bangladesh, encompassing CPW, RDW, healthcare workers, and retailers. Women in both contexts distinguished among micronutrient products based on colour, shape, packaging, and perceived purpose. In cognitive tests, women generally understood the English term ‘iron’ as a catch-all term for any iron-containing supplement. Women did not recognise any specific term for multivitamins or MMS. Shorter recalls of seven days or one month for adherence were feasible for respondents.

**Conclusions:**

It is challenging for the measurement community to identify terms and images that help women distinguish between supplement types. The final set of survey questions developed as part of this research is comprehensible and can be feasibly adapted and used in multi-topic surveys in MMS contexts, but requires validation.

Recent global estimates suggest 13.4 million livebirths were preterm (<37 completed weeks) and 23.4 million were small for gestational age (SGA) in 2020, while a substantial share were also low birth weight (LBW) [1]. To improve birth outcomes, the World Health Organization (WHO) recommends daily supplementation during pregnancy with iron-containing supplements in the form of iron and folic acid (IFA) or, in certain contexts, the United Nations International Multiple Micronutrient Antenatal Preparation (UNIMMAP) Multiple Micronutrient Supplements (MMS), a formulation of 15 micronutrients [2,3]. Daily calcium supplementation is also recommended in contexts with low intakes [4]. Countries are increasingly considering a transition from IFA to MMS based on strong evidence that the latter provides equal or greater benefits for LBW, preterm birth, and SGA, while addressing a wider range of micronutrient deficiencies [5,6]. However, expected benefits vary by context and may be mediated by implementation factors [5,6].

Country and global policy actors need reliable data on who is being reached with micronutrient supplement interventions during pregnancy, with nationally representative household surveys being an important source in this context. In a 2018 survey of individuals working in and across low- and middle-income countries, 75% of the 235 respondents reported accessing data on IFA coverage through the Demographic Health Survey (DHS) in the previous year [7]. The DHS Round 8 (DHS-8) core questionnaire includes three questions about iron-containing supplements, administered to women with a live birth in the last three years (Figure S1 in the **Online Supplementary Document**) [8]. A study in Nepal validating the accuracy of women’s responses to similar questions found that, at six months postpartum, women accurately reported whether they received any IFA during pregnancy, but significantly over-reported the total number of tablets consumed during pregnancy, leading to biased estimates of adherence [9]. Validation studies of questions about other antenatal care (ANC) services show that postpartum women’s recall is often inaccurate, particularly when the recall period is longer [10–13]. To improve the accuracy of adherence recall, the Nepal study’s authors recommend using shorter postpartum recall periods and posing questions to currently pregnant women (CPW) [14].

The transition from IFA to MMS adds another dimension of complexity to measuring iron-containing supplement coverage, requiring a distinction in the proportion of the population reached by each type of supplement. Some contexts also implement daily calcium supplementation during pregnancy.

There is limited evidence to guide the design of household survey questions that distinguish between different daily micronutrient supplements, which reflects a broader lack of investment in such formative and validation research [15,16]. Questions in the DHS and other global survey tools do not sufficiently account for the cognitive processes respondents use across settings to interpret questions and recall information. While global survey programmes field-test their tools and may conduct some cognitive testing, these multi-topic surveys lack the resources to conduct formative or validation research for every indicator [17]. While some guidance is available on question validation methods for maternal and newborn health, there is no established approach to conducting formative research to improve question design for reproductive, maternal, newborn, and child health and nutrition topics in household surveys. resulting in considerable variability in data quality [18].

In 2023–2024, the Data for Decisions in Nutrition project partnered with research teams in Ethiopia and Bangladesh to design a set of household survey questions with visual aids for inclusion in multitopic health and nutrition surveys to better account for how women recall supplement use during pregnancy [19]. The objectives of our formative research were to understand whether and how women in Ethiopia and Bangladesh differentiate between IFA, MMS, and calcium supplements; to understand the cognitive processes women use to recall whether and how they adhered to their supplement regimen during pregnancy; and to draft, test, and refine survey questions and visual aids.

Here, we use the term ‘coverage’ to refer to population-level reach of supplementation and distinguish receipt (was the supplement given or obtained), consumption/adherence (how many days per week/month the woman reports taking the supplement over a defined recall window), and duration (months of use). We focus on constructs that are plausibly measurable by self-report in household surveys, recognising that criterion validity for adherence metrics ultimately requires validation against objective measures.

## METHODS

### Setting

We conducted this study in urban settings in Addis Ababa, Ethiopia, and urban Dhaka and rural Sylhet and Khulna districts in Bangladesh. These locations were selected to engage women who had been exposed to various prenatal micronutrient supplement products across the health system and retail markets and who represented diverse socioeconomic groups.

Ethiopia has a persistently high prevalence of LBW (17.7%) and micronutrient deficiencies [20]. One in four (24%) women of reproductive age (15–49 years) has iron deficiency anaemia, while deficiencies in iodine (59%), folate (46%), and zinc (34%) are widespread [20–22]. As of 2024, the Government of Ethiopia is piloting an implementation of MMS through routine ANC services in 21 *woredas* (districts), including Addis Ababa, and across five regions: Gambella; Oromia; Sidama; Southern Nations, Nationalities, and Peoples; and Somali [23].

In Bangladesh, 22.6% of newborns are LBW [24]. Among non-pregnant non-lactating women (15–49 years), 4.8% have iron deficiency anaemia and 26.3% have calcium deficiency [25,26]. Locally manufactured MMS, branded as ‘FullCare’, has been available in Bangladesh since July 2021 through franchise pharmacies supported by non-governmental organisations in several districts. In addition, the Government of Bangladesh has conducted a demonstration project replacing IFA with MMS at ANC contacts in public facilities in two districts [27]. At the time of the study, MMS uptake remained low due to slow implementation and procurement challenges [24]. Government health facilities distribute preventative calcium supplements for pregnant women [26].

### Study design

**Figure 1:**
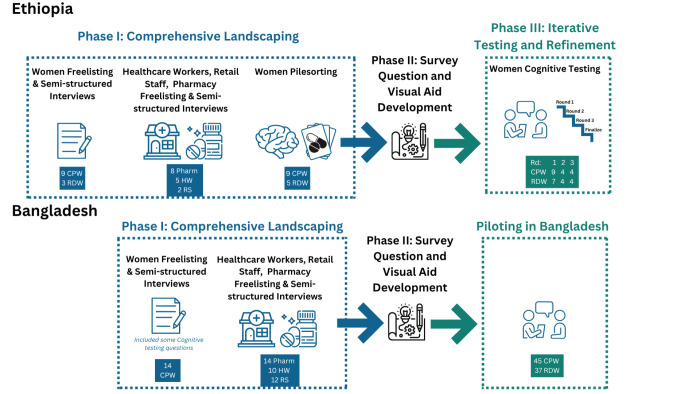
Designs of the studies in Ethiopia and Bangladesh.

The research process unfolded in three phases (**Figure 1**): phase I, comprehensive landscaping; phase II, survey question and visual aid development; and phase III, iterative testing and refinement. In Ethiopia, we completed all phases, while in Bangladesh, we drew on insights from Ethiopia and implemented a streamlined study. We selected some activities from phase I, all of phase II, and some components of phase III. This pragmatic decision, driven by time constraints and an interest in testing a more efficient model, provided valuable lessons about the scalability and adaptability of our methods.

The aim of phase I was to identify the micronutrient products (*e.g.* tablets, pills, syrups, powders) to which women were exposed during pregnancy. We conducted visits to healthcare facilities and retail outlets in each study site. At each location, we recorded brand names, dosage forms, packaging types, and labelled text for all maternal micronutrient products observed. Where possible, we obtained samples or took photographs to capture the packaging design, pill shape, and colour (Figures S3 and S4 in the **Online Supplementary Document**). We later incorporated these photographs into the pile-sorting exercise in Ethiopia and into visual aids in both countries. At each healthcare, pharmacy, or retail site, we completed freelisting exercises with staff to capture the terms and descriptions they use for products women take during pregnancy. Participants listed all products they knew, noted key product characteristics, and explained common reasons for use.

With women, we used freelisting and semi-structured interviews in both settings and pile sorting in Ethiopia only [28,29]. During freelisting, we asked women to list all products that pregnant women in their community are exposed to at health centres, pharmacies, and markets. For each item mentioned, we probed for local names, packaging formats, and distinguishing features. We then used semi-structured interviews to gain more detail about how women accessed, perceived, and used these products. For pile sorting, we asked participants to sort cards with names and product photos identified during the freelisting into piles based on perceived similarity. We probed to understand the rationale for grouping.

During phase II, we used phase I findings to adapt the DHS-8 survey questions for recently delivered women (RDW), create new questions for CPW, and design prototype visual aids. We focused on two issues: terms and images that help women differentiate between IFA, MMS, and calcium products in Bangladesh, and specifying a recall duration that was feasible for women to respond to. For the visual aids, we selected photos from products identified in phase I and grouped them into categories based on salient concepts from freelisting.

In phase III, we tested and refined the survey questions and visual aids. In Ethiopia, we completed three rounds of cognitive testing [15]. We showed women the draft visual aids alongside the questions and collected feedback on how they influenced question comprehension and response recall. The research team debriefed after each round and adjusted the questions and visual aids. In Bangladesh, survey questions and visual aids were piloted as part of survey preparation and adjusted based on participant feedback. By the conclusion of phase III, we had a refined set of survey questions for RDW and CPW and visual aids for each country context. All data collection tools are available in Texts S1–14 in the **Online Supplementary Document** and can be adapted for use in other contexts.

### Participants, sample size, and recruitment

We used purposive sampling in phases I and III to capture the perspectives of women of varying socioeconomic statuses. Sample sizes were pragmatic, balancing time and resources with the need to achieve saturation, ensure representation across outlet types, and capture variation in product access and descriptions. We did not use a formal saturation grid or pre-specified stopping rule.

In phase I, we included public, private, and non-governmental organisation facilities, prioritising sites that distributed MMS in the previous year. We also included private pharmacies and retail outlets (supermarkets, kiosks, market stalls). In Ethiopia, the phase I comprehensive landscaping was conducted across six health facilities, three pharmacies, and two retail shops in Addis Ababa. In Bangladesh, during phase I, we visited all 19 pharmacies/retail shops and 15 health centres within 1–2 km of the commercial centre. We interviewed on-site staff (*e.g.* healthcare workers, pharmacists, and retail staff), aiming to recruit one or two staff types per facility/outlet (Figure S2 in the **Online Supplementary Document**).

Phase I enrolled CPW in Ethiopia and Bangladesh and RDW in Ethiopia. Field research teams worked with staff at health facilities in study areas to identify CPW from ANC registers and, in Ethiopia, RDW using postnatal care registries; researchers invited eligible women on scheduled clinic days. We purposively selected women by age, location, education, and pregnancy stage (CPW in second or third trimester; RDW at ≤12 months postpartum) with the aim of achieving balanced diversity among participants and sufficient data for thematic and subgroup analyses. In phase III, in Ethiopia, we purposively sampled women who attended ANC/postnatal care during a period when MMS was available at health facilities. In Ethiopia, cognitive interview sample sizes followed the best practice of including 10–30 respondents in total [30].

In all rounds in Ethiopia and in phase I in Bangladesh, women were excluded from the study if they were under the age of 18, had a disability affecting verbal communication, or were experiencing a pregnancy-related emergency. Since phase III in Bangladesh was connected to a survey with a broader age range (including women 15–17 years of age), we aligned the criteria and excluded women under the age of 15 years. Women were only interviewed once (*e.g.* if a woman participated in a phase I freelist, she was excluded from subsequent phases).

### Analysis methods

We used FreeList Analysis under R Environment using Shiny (FLARES) software (open-source web-based software; AnthroCogs) to calculate Smith’s Salience Index [31]. Smith’s Index considers how often an item is mentioned and its position within individual lists, assigning greater weight to items listed earlier. We used researcher discretion to identify natural breaks in the salience scores of 0.25, but we did not treat this as a strict cut-point or use it to dichotomise items.

All semi-structured interviews were recorded and transcribed *verbatim* before translation into English. Local study co-investigators spot-checked them for accuracy. Two team members independently analysed interview transcripts in Excel using a thematic matrix with *a priori *developed codes. They then met to reconcile discrepancies through discussion and consensus. We did not calculate a formal intercoder reliability statistic, consistent with our matrix-based analytic approach. Transcripts were translated into English, so some nuance, especially local supplement terms, may have been lost despite spot-checking; we retained original terms where possible and clarified ambiguous wording with local co-investigators. We summarised coded segments in the matrix to enable comparison across respondents and sites and to identify recurring and divergent patterns.

We followed Trochim’s five-step process for pile sorting analysis and treated each product as a distinct unit of analysis [32]. We applied multidimensional scaling to the pile-sort data and used the its output to identify natural groupings or clusters of products. Researchers labelled the clusters based on participants’ stated rationales for grouping. The aim was to generate formative insight for instrument design rather than a definitive cognitive taxonomy.

We analysed cognitive interviews in Excel using pre-defined thematic matrix codes related to four types of cognitive errors: comprehension (does not understand the question), retrieval (cannot recall relevant information), judgment (feels uncomfortable responding; *e.g.* social desirability), and response (prespecified response items difficult for the respondent) [33]. Two team members independently analysed results after each round of interviews, compared them to identify cognitive errors in questions or visual aids, and made suggestions for improvement.

## RESULTS

### Sample and site characteristics

The background characteristics presented here reflect only the female participants (**Table 1**). In line with the recruitment criteria, most women were between the ages of 18 and 34 years, had varying education levels, and completed at least two ANC visits. Most CPW in Ethiopia were in their second or third trimester, while most CPW in Bangladesh were in their first or second trimester.

**Table 1:** Background characteristics of women from Ethiopia and Bangladesh

	Ethiopia										Bangladesh				
	**Freelist (n = 12)**		**Pilesort (n = 14)**		**Cognitive interview round 1 (n = 16)**		**Cognitive interview round 2 (n = 8)**		**Cognitive interview round 3 (n = 8)**		**Freelist (n = 14)**	**Pilot pretest (n = 9)**		**Pilot field Practice (n = 73)**	
	**CPW**	**RDW**	**CPW**	**RDW**	**CPW**	**RDW**	**CPW**	**RDW**	**CPW**	**RDW**	**CPW (n = 14)**	**CPW (n = 4)**	**RDW (n = 5)**	**CPW (n = 41)**	**RDW (n = 32)**
**Age in years**															
15–17	0	0	0	0	0	0	0	0	0	0	0	0	0	17	2
18–24	2	1	2	3	0	0	3	0	1	1	8	2	1	2	1
25–29	0	2	1	0	5	2	0	2	2	2	5	2	2	1	1
30–34	5	0	3	2	3	3	1	1	1	1	0	0	1	1	3
35–39	2	0	3	0	1	2	0	1	0	0	1	0	1	3	6
40–49	0	0	0	0	0	0	0	0	0	0	0	0	0	15	19
**Education**															
No education	0	0	0	1	2	1	1	1	1	1	2	0	0	0	0
Primary education	1	1	2	0	3	3	3	3	2	2	4	1	0	25	16
Secondary education	3	1	2	2	2	1	0	0	1	1	4	1	1	11	11
Higher education	5	1	5	2	2	2	0	0	0	0	4	2	4	3	5
**Trimester**															
First trimester (months 0–3)	0	NA	0	NA	0	NA	0	NA	0	NA	6	0	NA	18	NA
Second trimester (months 4–6)	3	NA	3	NA	3	NA	1	NA	1	NA	5	3	NA	21	NA
Third trimester (months ≥7)	6	NA	6	NA	6	NA	3	NA	3	NA	2	1	NA	2	NA
Do not know	0	NA	0	NA	0	NA	0	NA	0	NA	1	0	NA	0	NA
**Number of ANC visits**															
No visits												2	0	1	3
One visit	1	0	0	0	0	0	0	0	0	0	3	0	0	15	3
Two to three Visits	6	0	4	0	6	1	3	0	2	0	4	0	2	19	8
Four or more visits	2	2	5	5	2	5	1	3	2	4	5	2	3	2	18
Do not know	0	1	0	0	1	1	0	1	0	0	2	0	0	0	0
**Place of ANC**															
Private health facility	0	0	3	0	0	0	0	0	0	0	4	0	2	18	6
Public health facility	9	2	6	3	9	6	4	3	4	4	6	2	3	24	26
NGO	0	1	0	2	0	1	0	1	0	0	0	0	0	0	1
Home	0	0	0	0	0	0	0	0	0	0	3	0	0	4	3
Pharmacy	0	0	0	0	0	0	0	0	0	0	1	0	0	1	0

ANC – antenatal care clinic, CPW – currently pregnant women, NGO – non-governmental organisation, RDW – recently delivered women

### Phase I results

#### Availability of maternal micronutrient products

The range of products reported during freelisting and market landscaping in Ethiopia was relatively limited; respondents mostly reported iron-containing products. Women listed an average of 1.8 products each, covering seven different products, and did not mention calcium. Women typically used general terms like ‘vitamin’ rather than specifying a specific product type or brand. Healthcare workers listed an average of 3.4 products each, covering 10 distinct products, and pharmacy staff listed an average of 4.6 products for a combined 17 different products.

Bangladesh had a larger number of products identified during freelisting and market landscaping. Women listed an average of 2.6 products each, covering 10 different products; healthcare workers listed an average of 2.8 product types, with 23 different products; pharmacy staff listed an average of 4.8 product types (54 products); and retail staff listed an average of 6.6 product types (78 products), for a combined 132 products.

**Figure 2:**
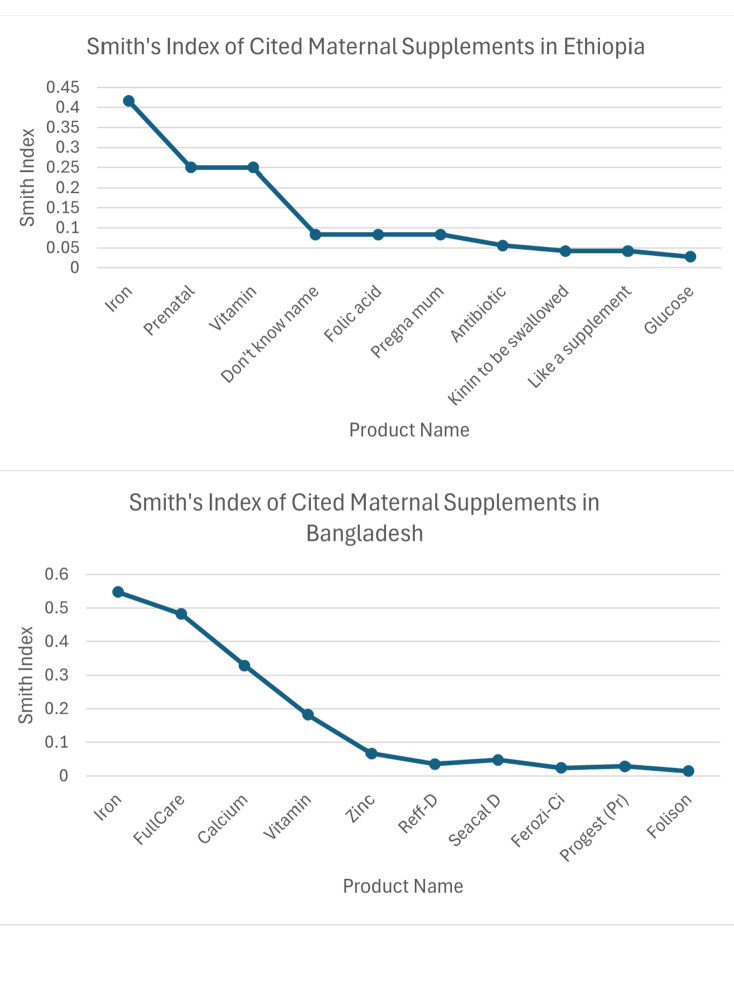
Women’s Smith’s Salience Index of Cited Maternal Supplements in Ethiopia (n = 12) and Bangladesh (n = 14).

For Smith’s Salience Index for freelisting by women in Ethiopia, we present salience values descriptively and note an apparent natural break around 0.25 as an interpretive reference point (**Figure 2**). These terms included ‘iron’ (0.42), ‘prenatal’ (0.25), and ‘vitamin’ (0.25). In comparable results from Bangladesh, most salient terms included ‘iron’ (0.55), ‘FullCare’ (0.48), and ‘calcium’ (0.33). The term ‘vitamin’ was also commonly cited in Bangladesh, with a Smith’s Index of 0.18.

#### Differentiation between micronutrient products

In Ethiopia, women consistently used the English term ‘iron’ and identified its purpose as treating iron deficiency. Most reported receiving iron in blister packs, with some variation in the reported colour of the pill (*e.g.* red, yellow, or brown). Women did not use the term MMS in the freelist. The four women who listed the term ‘prenatal’ described it as oval-shaped and brown. In contrast, the term ‘vitamin’ was used to describe heterogeneous products, including different colours, sizes, and packaging. In Ethiopia, pile-sort (n = 14) women created, on average, 2.2 unprompted piles. Hierarchical cluster analysis revealed two main groupings, labelled based on participant explanations: 1. ‘vitamins,’ which included the following products: Prenatal, Nature Made Prenatal, UNIMMAP MMS, Promam, Pregnavit, Iron Folate, and Fenza; and ‘iron/blood’, which included the following products: Iron Folate, Haem-up, UNIMMAP MMS, Nature Made Prenatal, Femia, and Tesha-5 (Figure S3 in the **Online Supplementary Document**). The overlap in supplement composition across groupings (*e.g.* IFA *vs*. MMS *vs*. other multivitamins) suggests that women do not recognise composition as a distinguishing factor among groups.

In Bangladesh, women consistently described ‘iron’ as round and small, but the reported colour varied from dark brown to red to pink/white mixed. All women could describe the purpose of iron and reported receiving iron in blister packs. ‘Calcium’ was uniformly described as big, white, and in a blister pack. ‘FullCare’ was also uniformly described as tall, big, white, and in red or pink packaging, with an image of a pregnant woman. The eight women who mentioned FullCare described it as having a mix of ingredients. In contrast, ‘vitamin’ had no uniform description, with women describing it as yellow, pink, or white, and saying it came in either blister packs or packets of different colours.

#### Women’s approach to adherence recall

When asked how they recalled the number of pills taken, women in Ethiopia described estimating their intake based on the number of months they were instructed to take the product, the expected number of tablets per month (typically 30), and the daily routines they followed. Adherence was often anchored to ANC visits, starting with the first visit and continuing for one- or three-month durations. A month’s supply was described as three blister packs of 10 tablets each. Some women reported missing doses due to illness or disruptions in their daily routine. When supplements were provided in bottles, women estimated their intake by the number of full bottles they completed. One CPW in Ethiopia stated, ‘I took it every day after dinner, so I didn’t forget to take it after dinner. And also, my daughter reminds me to take it. I didn’t skip any of the pills. They give me three strips last month.’

Similarly, women in Bangladesh anchored adherence to their first ANC visit and the number of blister packs completed; they reported taking it every day as instructed by the clinic. Reasons that they missed tablets included being busy at home or traveling. One CPW in Bangladesh reported: ‘By looking at the blisters toward the end of the month, I get to count if I missed taking any medicines during that period.’

### Phase II results

#### Design of prototype survey questions and visual aids

For Ethiopia, we adapted RDW prototype questions from the DHS-8 IFA questions, and we designed questions with similar constructs for CPW, *e.g.* type, source, adherence (Figure S1 in the **Online Supplementary Document**). We planned to reduce the number of questions in the final set through the phase III cognitive testing process.

To improve product identification, we began with a two-question sequence using the broad term ‘vitamin tablets or syrups’, followed by prompts about categories such as ‘iron and folic acid’, ‘prenatal’, ‘folic acid’, and ‘any other vitamin’, reflecting salient terms from phase I. We included a question about the month of pregnancy when supplementation began to produce an indicator of ‘initiation in the first trimester’ and to help anchor recall of the total amount consumed. For CPW, we asked about consumption over the last week and last month; for RDW, we focused on total months of consumption during their most recent pregnancy and asked about intake during a ‘typical’ week and month. We also included questions that explicitly asked about ‘missed days’.

In Bangladesh, we started with the final question set from Ethiopia and used phase I findings to adapt terminology to better reflect the local context. Specifically, we used the terms ‘MMS tablet or FullCare’, ‘supplements with multiple micronutrients’, and ‘iron tablets or syrups’ to align with products commonly recognised by participants.

**Figure 3:**
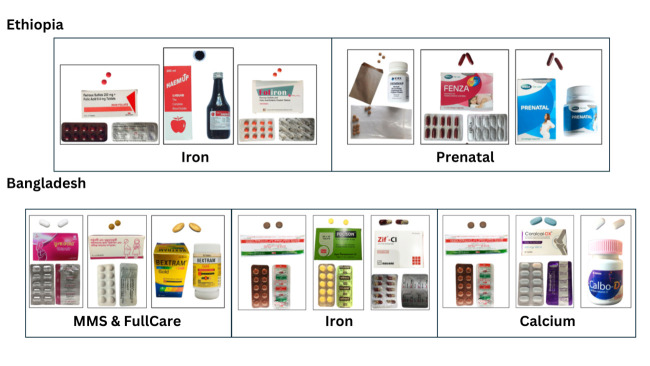
Final visual aids for Ethiopia and Bangladesh.

We designed visual aid prototypes to complement the survey questions. Key design decisions included how many products to show, the overall layout, and how to represent different product categories. Product selection drew on freelisting and pile-sort results, contextual relevance, and opportunities to group products into different categories (Table S3 in the **Online Supplementary Document**). For example, because women reliably recognised iron supplements and MMS by colour and packaging, we included those characteristics in the images (**Figure 3**).

### Phase III results

We identified two main areas of cognitive challenge in Ethiopia and Bangladesh through cognitive interviews or piloting: understanding of terminology and timing of initiation, temporal recall of duration, and frequency of product intake (**Table 2**; Tables S1 and S2 in the **Online Supplementary Document**).

**Table 2:** Summary of cognitive interview results

	Key findings and adaptations made
**Comprehension of terminology**	
Terminology: ‘vitamin’, ‘iron’, ‘folic acid’, ‘prenatal’	In ET, ‘iron’ was well understood, but ‘folic acid’ and ‘prenatal’ were inconsistently interpreted; ‘multivitamin’ was not understood. Adapted ET to ‘iron or iron-folic acid’ and ‘iron with many vitamins or prenatal’. In BD, ‘iron’, ‘medicine’, or specific local names were commonly used. Final BD questions included the terms ‘MMS tablet or FullCare’, ‘supplements with multiple micronutrients’, and ‘iron tablet or syrup.’
Visual aid use	In ET visual aids improved comprehension and response plausibility. Women relied on packaging form (bottle, loose pills, blister packs) for supplement identification. Visual aid revised to show real-world formats including blisters, bottles and loose pills repackaged in sachet or envelope. It was challenging to group images into categories women intuitively understood; many interpreted the images as representing specific products rather than broader categories (*e.g.* IFA, MMS)
**Temporal recall**	
Timing of initiation (start)	In ET and BD, month-based recall was better understood than week-based. Dropped ‘weeks’ and retained ‘months’ in questions.
Duration of intake	In ET, women could recall number of months taken; responses were consistent with cross-check questions. Question retained in final tool.
Usual intake	In ET, women could report how many days in a ‘usual week’ or ‘usual month’ they took the supplement in their previous pregnancy using an open-ended response; this provided more plausible responses than categorical responses of ‘daily’ or ‘always. Question shifted to asking about ‘usual’ with open-ended responses
Seven-day recall/weekly intake	In ET, open numeric recall (report 0–7 days/week) provided more plausible responses than categorical responses (*e.g.* sometimes; 1–2 days). Open response format retained, categorical format dropped.
Seven-day recall/monthly recall/total days taken over pregnancy	In ET, CPW provided more plausible responses to seven-day recall compared to a month or 30-day recall; RDW women overestimated response to total days of pregnancy or said it was too difficult to answer. The total days of pregnancy question was dropped. In BD, CPW provided plausible responses to both seven-day and month recall, total days of pregnancy were overestimated or difficult to answer.

BD – Bangladesh, CPW – currently pregnant women, ET – Ethiopia, IFA – iron folic acid, MMS – multiple micronutrient supplement, RDW – recently delivered women

#### Understanding product terms

We faced challenges throughout the question design process in identifying terms for different supplement types that women in Ethiopia and Bangladesh consistently understood. In Ethiopia, the English term ‘iron’ was commonly used by participants (as reflected in transcripts) and was generally understood as referring to any iron-containing supplement (*e.g.* IFA, MMS), while terms like ‘iron folic acid’, ‘prenatal’, ‘MMS’, and ‘multivitamin’ were not familiar. One CPW in Ethiopia explained, ‘Iron is beneficial for the foetus and for the mother… but I don’t know what folic acid is.’ In Ethiopia, more general terms, including ‘vitamin’ and ‘supplement’, were also not well understood, with some women equating supplements to foods like fruits or dates. In Bangladesh, the terms ‘iron’, ‘calcium’, ‘medicine’, or specific local names (*e.g.* Fullcare for MMS) were commonly used.

In the final set of questions in Ethiopia, we refined the terminology for the two supplement types to ‘iron or folic acid’ and ‘iron with many vitamins or prenatal’. In the final set of questions in Bangladesh, we refined the terminology for three supplement types to ‘FullCare’, ‘supplements with multiple micronutrients’, and ‘iron tablet or syrup’. In Bangladesh, we added a separate set of questions for ‘calcium’ (Table S4 in the **Online Supplementary Document**).

In Ethiopia, when reviewing the visual aid, women recognised iron and prenatal supplements by packaging, colour, and size. To improve clarity, we added borders around each product image to distinguish products within categories. We included multiple packaging forms for products (*e.g.* loose pills, blisters, cartons, bottles) and accounted for products repackaged by health workers into small paper and plastic bags (**Figure 3**). Women often referred to the category, including MMS, as ‘iron’. They tended to look for an image that was an exact match to what they had taken and did not recognise products within a category as similar to what they had taken. Despite these challenges, participants and enumerators found the visual aid intuitive and easy to use. In Bangladesh, participants did not report any challenges with using the visual aid.

#### Temporal recall

In Ethiopia, we tested questions RDWs related to three dimensions of recall: timing of initiation (when women start to take a supplement); duration (length of time taking a supplement); and frequency (how often women take the supplement). For CPW, we tested similar questions for initiation and frequency.

For timing of initiation, we cognitively tested the question, ‘During your last pregnancy (RDW)/current pregnancy (CPW), how many months pregnant were you when you first started taking (INSERT SUPPLEMENT TYPE)?’ The question demonstrated high levels of comprehension, and women often linked the start of their supplement intake to when they first visited ANC.

For duration of consumption, during round I of cognitive testing, we asked RDW, ‘During your last pregnancy, how many months or weeks did you take vitamin tablets or syrup?’ The results showed good comprehension; women were more likely to recall the duration in months rather than weeks. We refined the question to only ask about ‘months’.

Regarding frequency of consumption, for RDW, recalling the total number of days during an entire pregnancy that women took supplements proved cognitively demanding, and the question was removed. When we asked RDW and CPW to report frequency using categorical response options such as ‘daily’ or ‘a few days a week’, nearly all women responded ‘daily’. Further probing revealed that some women responding ‘daily’ had skipped doses. In the second round, we included follow-up questions such as ‘Were there days you did not take the tablet or syrup?’ and ‘If yes, how often did you miss?’ Initially, all participants responded ‘no’, suggesting perfect adherence. However, probing revealed missed days, indicating that direct yes/no questions failed to capture actual behaviour.

We found open-ended questions with shorter recall periods to be more comprehensible and plausible. Asking RDW about ‘days in a usual week’ led to more consistent responses about adherence. We ultimately decided to ask about a ‘usual week’ rather than a ‘usual month’ because responses to the week question showed greater variability, suggesting women were not defaulting to ‘seven days’ or ‘all week’. For CPW, we compared asking about the number of days ‘during the last month’ to ‘during the last week’. The CPW reported that recalling their actual behaviour over these periods was straightforward, with recall for the ‘last week’ being easier. Here, ‘plausible’ refers to internally coherent responses across related probes (*e.g.* initiation month, months of use, and reported weekly intake) and less defaulting to ‘daily’. Plausibility reflects improved comprehension and response process. Yet, it still requires validation against objective measures (*e.g.* pill counts/blister packs or records).

In Bangladesh, to estimate how many days they took supplements, women commonly relied on strategies such as counting remaining pills in blister packs. Routines around consumption (*e.g.* take it every day before bed) also played a role in recalling adherence.

The final question sets for Ethiopia and Bangladesh differed in the number of questions and in the specific terms used to distinguish between the IFA and MMS categories (**Table 3**).

**Table 3:** Final question set for women from Ethiopia and Bangladesh

RDW				
**Number**	**Question**		**Response option**	
1	During your last pregnancy, were you given, or did you buy any tablet or syrup that contains iron (*show visual aid*)?		Yes (1); no (2); don’t know (8)	
2	During your last pregnancy, were you given or did you buy any of the following: A) BD – MMS tablet or FullCare; B) BD – supplements with multiple micronutrients | ET – iron with many vitamins or prenatal; C) BD – iron tabled or syrups | ET – iron or iron folic acid (*show visual aid*)		A to C – Yes (1); no (2); don’t know (8)	
3	During your last pregnancy, how many months pregnant were you when you first started taking (*list product if response to Q2 is ‘yes’*)?		Months ( ); don’t know (98)	
4	During your last pregnancy, how many months did you take (*list product if response to Q2 is ‘yes’*)?		Months ( ); don’t know (98)	
5	During your last pregnancy, how many days in a usual week did you take (*list product if response to Q2 is ‘yes’*)?		Days ( ); don’t know (998)	
	During your last pregnancy, where did you get these (*list product if response to Q2 is ‘yes’*)? Anywhere else?		List modified from DHS-8 model woman's questionnaire (Q427)	
**CPW**				
**No.**		**Question**		**Response option**
1		During your last pregnancy, were you given or did you buy any tablet or syrup that contains iron (*show visual aid*)?		Yes (1); no (2); don’t know (8)
2		During your last pregnancy, were you given or did you buy any of the following: A) BD – MMS tablet or FullCare; B) BD – supplements with multiple micronutrients | ET – iron with many vitamins or prenatal; C) BD – iron tabled or syrups | ET – iron or iron folic acid (*show visual aid*)		A to C – yes (1); no (2); don’t know (8)
3		During this pregnancy, how many months pregnant were you when you first started taking (*list product if response to Q2 is ‘yes’*)		Months ( ); don’t know (98)
4		How many days did you take the (*list product if response to Q2 is ‘yes’*) last MONTH?		Days ( ); don’t know (998)
5		During this pregnancy, where did you get these (*list product if response to Q2 is ‘yes’*)? Anywhere else?		List modified from DHS-8 model woman's questionnaire (Q427)

BD – Bangladesh, CPW – currently pregnant women, ET – Ethiopia, DHS-8 – District Health System Round 8, MMS – multiple micronutrient supplement, RDW – recently delivered women

## DISCUSSION

Our findings reiterate the importance of changing the standard DHS-8 question for iron-containing supplements during pregnancy to improve comprehension and reduce common response errors in self-report adherence measures. They also show that it is difficult for women to distinguish among iron-containing supplement products. Using locally understood terms is important, but in the case of MMS, no distinct term was consistently recognised by women in either Ethiopia or Bangladesh. A validation study in Nepal similarly emphasised that maternal recall of supplement receipt was only moderately valid when survey questions were not adapted to reflect local systems and terminology [34].

In both countries, the English term ‘iron’ was frequently used as a catch-all term for any iron-containing prenatal supplement, including MMS. This lack of differentiation presents a risk of misreporting. As MMS products are introduced and scaled by governments, distinct and consistent branding and labelling compared to IFA may reduce respondent confusion; this is a hypothesis-generating implication of our findings that warrants further study. This may be challenging in Bangladesh, as all supplements distributed by the government that we observed had very similar packaging designs.

The lack of specific terminology reinforces the importance of visual aids to help with recall. Historically, DHS surveys showed respondents physical samples of IFA tablets. With the addition of MMS and, in some countries, calcium, this may no longer be practical. A major challenge in designing the visual aid was identifying a reduced set of images that represent the wide variety of available products. It was unclear whether women understood the images as representing a category of products or were looking for specific products.

The proliferation of supplement types and brands across healthcare and retail settings raises questions about intervention specification. As UNIMMAP MMS is being introduced and scaled across countries, other prenatal vitamins containing multiple micronutrients are already available in pharmacies and retail settings. Restricting measurement to UNIMMAP MMS formulations is in line with WHO protocols, but may underestimate coverage where similar but not equivalent formulations are distributed. This may be less of a concern in contexts where most women receive ANC at public-sector facilities using standard products. Also, it does not appear that women can distinguish between UNIMMAP and non-UNIMMAP formulations unless UNIMMAP products use and promote very distinct branding.

Women in Ethiopia and Bangladesh were able to easily answer the final set of questions on supplement initiation, duration, and frequency. We recommend replacing the current DHS-8 Q428 (Figure S1 in the **Online Supplementary Document**) with up to three questions (**Table 3**) about the total number of months supplements were consumed, the timing of initiation, and usual intake. We believe these questions will yield more accurate and useful information regarding initiation and adherence than the existing question on the total number consumed during pregnancy. It is important to note that estimates from the revised timing and adherence questions will not be directly comparable to those from the DHS-8 question. One option is to field the previous and revised questions together for a single survey round (‘bridge’ study) to calibrate results and document expected shifts. However, because the existing question has been shown to yield inaccurate estimates, we prioritise improving measurement accuracy even if comparability is reduced in the short term.

Posing survey questions to CPW is innovative, but also entails trade-offs in survey design. During household listing and sampling, it may be challenging to accurately identify pregnant women and obtain a sufficient sample size for sub-national representativeness. Importantly, interviewing CPW may reduce recall error for proximal behaviours, but it can introduce biases, such as overrepresentation of ANC-attending women. Therefore, to improve estimates and strengthen inferences about supplement adherence, we recommend including CPW questions in addition to, not instead of, RDW questions.

Uptake of new indicators will need to be widely socialised and supported technically. We can learn from successful efforts to change anthropometric indicators from the National Center for Health Statistics growth reference to the WHO growth standard. However, in that case, it was possible to recalculate comparable indicators in past datasets. During the transition to new growth indicators, there was a period of parallel reporting of both new and old indicators, along with the issuance of bulletins and guidance. Similar processes can be applied to the new CPW indicators and the revised RDW indicators.

### Strengths and limitations

This study was designed to be incremental and iterative, with multiple phases of data collection that informed and shaped each subsequent phase. This approach allowed us to refine our tools and insights in real time, increasing the relevance and rigour of our findings in two distinct settings.

The study also has some limitations. All Ethiopian data were collected in urban settings, which limits our ability to extrapolate findings to rural populations. Differences in health system access, supplement exposure, and sociocultural norms in rural areas may affect the relevance and applicability of our findings. In addition, our site selection prioritised areas with greater exposure to multiple supplement products across health system and retail markets; transferability to settings with limited product diversity or different repackaging practices will require additional adaptation and testing. We also did not conduct a formal rural/urban comparative analysis within Bangladesh. We did not conduct full cognitive testing in Bangladesh, and cross-country differences should not be attributed to context alone, given possible method-driven variation. Further, we cannot state the degree to which these new measures elicited more accurate results. Before these questions can be used, at-scale validation studies are essential.

## CONCLUSIONS

This study contributes to the growing body of evidence on improving health and nutrition, both generally and specifically for maternal micronutrient supplement coverage. The results highlight the need to tailor maternal micronutrient supplement coverage questions to match how women recognise and recall products. Product recognition is influenced by packaging and branding, underscoring the importance of using locally adapted language and visual aids. Accurate reporting also requires minimising cognitive burden by aligning questions with how women think about their supplement use. Our approach provides a scalable way to develop context-adapted survey items for measuring nutrition intervention coverage.

## Additional material


Online Supplementary Document
