# Supplementary material for: Formative research in Ethiopia and Bangladesh: strengthening household survey measures of maternal micronutrient supplementation in low- and middle-income countries
Source: J Glob Health. 2026 Jul 3;16:04159. doi: 10.7189/jogh.16.04159 (PMC13329879; doi:10.7189/jogh.16.04159)
Supplement: Online Supplementary Document [file jogh-16-04159-s001.pdf]

Supplement to: Walton S, Wood E, Berhane H, Firehiwot W, Ferdaweke F, Billah S, Lotus S, Munos, M, Kim S, Manohar S, Nguyen P, Azeem, S, Heidkamp R. Formative research in Ethiopia and Bangladesh: strengthening household survey measures of maternal micronutrient supplementation in low- and middle-income countries. J Glob Health. 2026;16:04159.

Table of Contents

1. Figure S1: Core DHS Maternal Micronutrient Supplementation Questions
2. Figure S2 : Ethiopia & Bangladesh Study Participants
3. Figure S3: Ethiopia Salient Product List
4. Figure S4: Bangladesh Salient Product List
5. Table S1: Ethiopia Cognitive Interview Findings & Survey Revisions for Currently Pregnant Women
6. Table S2: Ethiopia Cognitive Interview Findings & Survey Revisions for Recently Delivered Women
7. Table S3: Summary of Visual Aid Testing and Revisions in Ethiopia and Bangladesh
8. Table S4: Bangladesh Calcium Questions used in One Nutrition Coverage Survey
9. Text S1: Freelisting Semi-Structured Interview, Bangladesh
10. Text S2: Landscaping Guide, Bangladesh
11. Text S3: Landscaping Guide, Ethiopia
12. Text S4: Freelisting Semi-Structured Interview, Ethiopia
13. Text S5: Pile-Sorting Semi-Structured Interview, Ethiopia
14. Text S6: Round 1 Cognitive Interview CPW, Ethiopia
15. Text S7: Round 1 Cognitive Interview RDW, Ethiopia
16. Text S8: Round 1 Cognitive Interview Module 4, Ethiopia
17. Text S9: Round 2 Cognitive Interview CPW, Ethiopia
18. Text S10: Round 2 Cognitive Interview RDW, Ethiopia
19. Text S11: Round 2 Cognitive Interview Module 4, Ethiopia
20. Text S12: Round 3 Cognitive Interview CPW, Ethiopia
21. Text S13: Round 3 Cognitive Interview RDW, Ethiopia
22. Text S14: Round 3 Cognitive Interview Module 4, Ethiopia

Figure S1: Core DHS Maternal Micronutrient Supplementation Questions

426<br>(3)	During this pregnancy, were you given or did you buy any iron tablets or iron syrup?<br><br>SHOW TABLETS/SYRUP/MULTIPLE MICRONUTRIENT SUPPLEMENT.	YES . . . . 1<br>NO . . . . 2<br>DON'T KNOW 8	→ 429 (NO, DON'T KNOW)
427<br>(1)<br>(3)	Where did you get the iron tablets or syrup?<br><br>Anywhere else?<br><br>PROBE TO IDENTIFY THE TYPE OF SOURCE.<br>IF UNABLE TO DETERMINE IF PUBLIC, PRIVATE, OR NGO SECTOR, RECORD 'X' AND WRITE THE NAME OF THE PLACE(S).	**PUBLIC SECTOR**<br>GOVERNMENT HOSPITAL . . . . A<br>GOVERNMENT HEALTH CENTER . . . . B<br>GOVERNMENT HEALTH POST . . . . C<br>MOBILE CLINIC . . . . D<br>COMMUNITY HEALTH WORKER/FIELD WORKE E<br>OTHER PUBLIC SECTOR F<br>__________<br>(SPECIFY)<br><br>**PRIVATE MEDICAL SECTOR**<br>PRIVATE HOSPITAL . . . . G<br>PRIVATE CLINIC . . . . H<br>PHARMACY . . . . I<br>PRIVATE DOCTOR . . . . J<br>MOBILE CLINIC . . . . K<br>COMMUNITY HEALTH WORKER/FIELD WORKE L<br>OTHER PRIVATE MEDICAL SECTOR M<br>__________<br>(SPECIFY)<br><br>**NGO MEDICAL SECTOR**<br>NGO HOSPITAL . . . . N<br>NGO CLINIC . . . . O<br>OTHER NGO MEDICAL SECTOR P<br>__________<br>(SPECIFY)<br><br>**OTHER SOURCE**<br>SHOP . . . . Q<br>MARKET . . . . R<br>[MASS DISTRIBUTION CAMPAIGN] . . . . . S<br><br>OTHER __________ X<br>(SPECIFY)	
428<br>(3)<br>(4)	During the whole pregnancy, for how many days did you take the iron tablets or syrup?<br><br>IF ANSWER IS NOT NUMERIC, PROBE FOR APPROXIMATE NUMBER OF DAYS.	DAYS . . . . [ ][ ][ ]<br><br>DON'T KNOW 998	

Figure S2 : Ethiopia & Bangladesh Study Participants

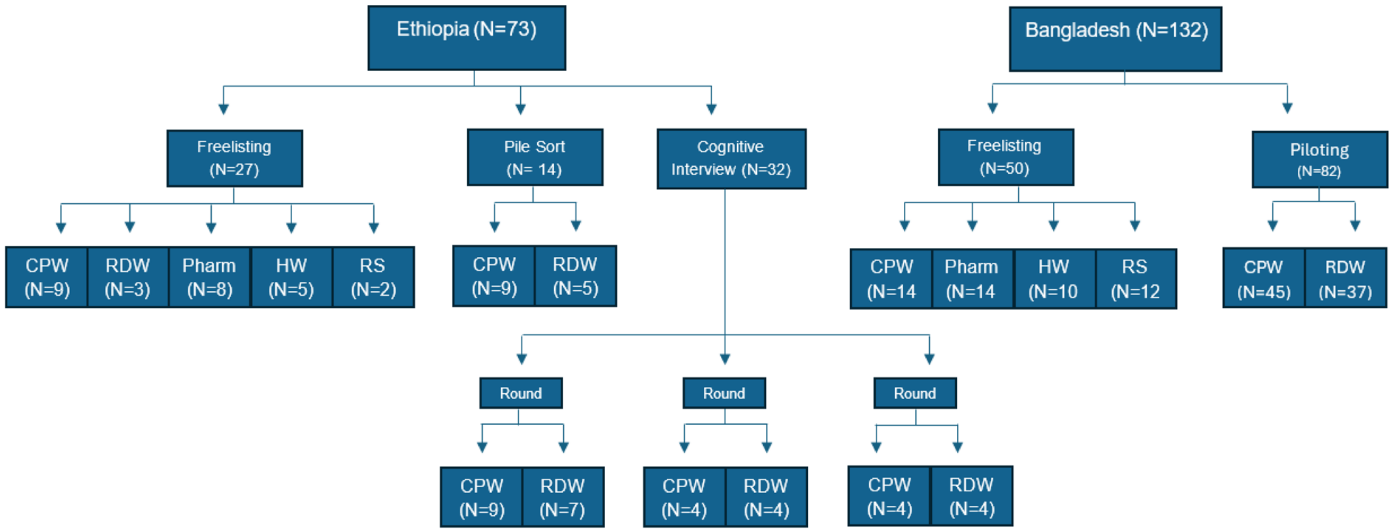

Figure S3: Salient Product List, Ethiopia

Formal Product Name	Formulation	Image
Iron and Folic Acid		
Haem Up	20 mg ferrous ascorbate (equivalent to 20 mg elemental iron), 1.5 mg folic acid, 15 mcg cyanocobalamin, 100 mg lysine	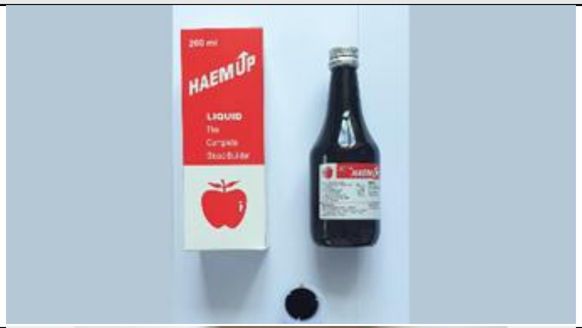
Iron folate	200 mg ferrous sulfate, 0.4 mg folic acid	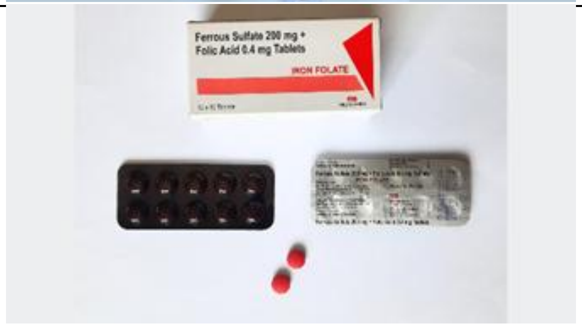

Formal Product Name	Formulation	Image
Foliron	150 mg of ferrous sulfate (55 mg iron), 0.5 mg folic acid	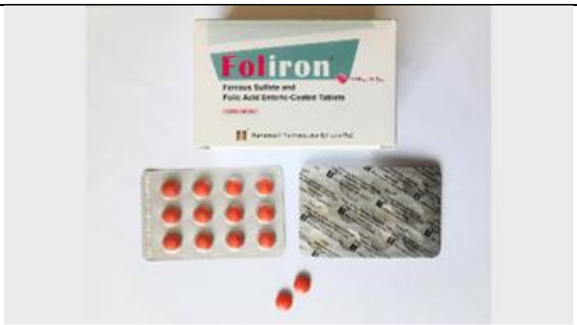
Prenatal Multivitamin		
UNIMMAP	800 µg vitamin A, 200 IU vitamin D, 10 mg vitamin E, 18 mg niacin, 400 µg folic acid, 1.4 mg vitamin B1 (thiamine), 1.4 mg vitamin B2 (riboflavin), 1.9 mg vitamin B6 (pyridoxine), 2.6 µg vitamin B12, 70 mg vitamin C, 15 mg zinc, 30 mg iron, 65 µg selenium, 2 mg copper, 150 µg iodine	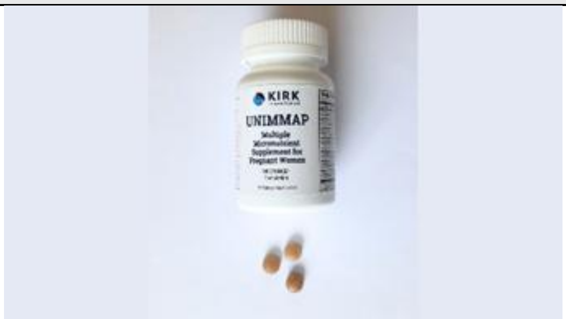
Prenatal	4000 IU vitamin A, 400 IU vitamin D, 2 mg vitamin B1, 2 mg vitamin B2, 10 mg Nicotinamide, 2 mg vitamin B6, 2 mg calcium pantothenate, 0.25 mg folic acid, 37.50 mg vitamin C, 2 mcg vitamin B12, 100 mg calcium (as calcium carbonate), 1 IU vitamin E, 20 mg Iron (as ferrous sulphate), 0.5 mg Copper (As copper sulphate), 0.05 mg Iodine (as potassium iodide), 1 mg magnesium (as magnesium sulphate), 0.1 mg manganese (as manganese sulphate), 1 mg potassium (as potassium sulphate), 0.15 mg zinc (as zinc sulphate)	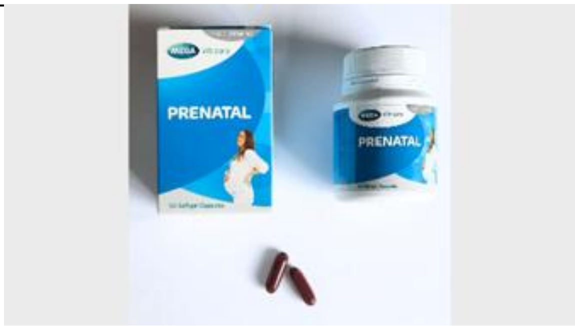

Formal Product Name	Formulation	Image
Fenza	3 mg Natural mixed carotenoids (3822.71 IU Equivalent to Vitamin A),<br>5 IU Vitamin E (d-alpha tocopheryl acetate),<br>200 IU Vitamin D3,<br>5 mg Vitamin B1 (as Thiamine mononitrate),<br>2.5 mg Vitamin B2 (Riboflavin),<br>20 mg Vitamin B3 (Nicotinamide),<br>5 mg Vitamin B6 (Pyridoxine hydrochloride),<br>0.004 mg Vitamin B12 (Cyanocobalamin), 0.25 mg Folic acid,<br>100 mg Vitamin C (Ascorbic acid),<br>6.9 mg Pantothenic acid,<br>50 mg Calcium,<br>20 mg Magnesium,<br>5 mg zinc,<br>0.1 copper,<br>0.1 iodine,<br>0.15 Biotin<br>15 mg iron (as ferrous fumarate),<br>300 mg Tuna oil (25% DHA)	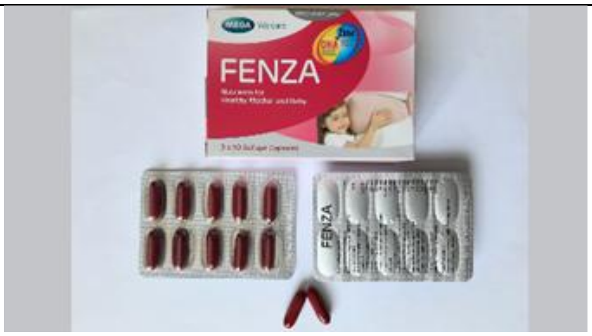

Figure S4: Salient Product List, Bangladesh

Formal Product Name	Formulation	Image
Iron and Folic Acid		

IFA	200 mg Ferrous Fumarate and 400 ug Folic Acid	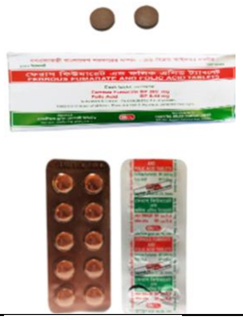
Folison	5 mg Folic acid	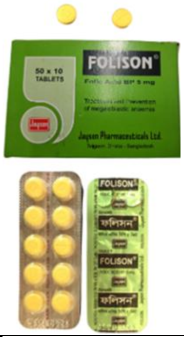
Zif-Cl	50 mg Carbonyl Iron + .5 Folic Acid + 61.8 Zinc Sulfate	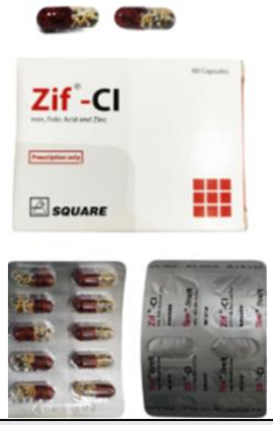
Prenatal Multivitamin		
FullCare	800 µg vitamin A, 200 IU vitamin D, 10 mg vitamin E, 18 mg niacin, 400 µg folic acid, 1.4 mg vitamin B1 (thiamine), 1.4 mg vitamin B2 (riboflavin), 1.9 mg vitamin B6 (pyridoxine), 2.6 µg vitamin B12, 70 mg vitamin C, 15 mg zinc, 30 mg iron, 65 µg selenium, 2 mg copper, 150 µg iodine	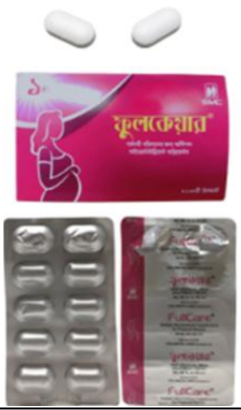

MMS Tablet	800 µg vitamin A, 200 IU vitamin D, 10 mg vitamin E, 18 mg niacin, 400 µg folic acid, 1.4 mg vitamin B1 (thiamine), 1.4 mg vitamin B2 (riboflavin), 1.9 mg vitamin B6 (pyridoxine), 2.6 µg vitamin B12, 70 mg vitamin C, 15 mg zinc, 30 mg iron, 65 µg selenium, 2 mg copper, 150 µg iodine	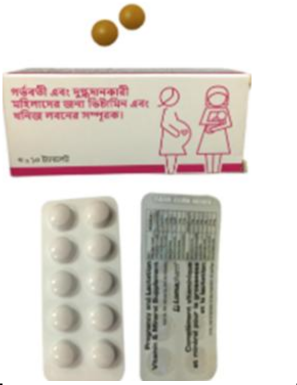
Bextram Gold	Vitamin A, Vitamin C, Vitamin D, Vitamin E, Vitamin K, Vitamin B1 (Thiamine), Vitamin B2 (Riboflavin), Vitamin B3 (Niacin), Vitamin B6 (Pyridoxine), Folic acid, Biotin, Pantothenate, Calcium, Iron, Phosphorous, Iodine, Magnesium, Zinc, Selenium, Copper, Manganese, Chromium, Molybdenum, Chloride Potassium, Boron, Nickel, Silicon, Tin, Vanadium, Lutein and Cyanocobalamin.	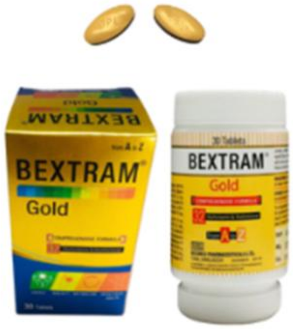
Calcium		
Calcium	500 mg calcium carbonate	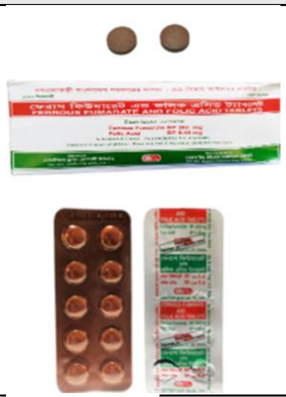
Calbo-D	500 mg elemental calcium + 200 IU vitamin D3	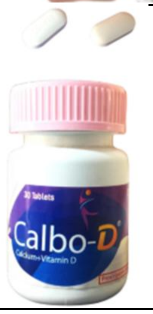

Coralcal-D	500 mg coral calcium + 200 IU vitamin D3	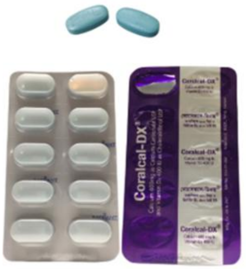

Table S3: Summary of Visual Aid Testing and Revisions in Ethiopia and Bangladesh

Dimension tested	Results	Resulting adaptation
**Recognition of Iron Products**	In both Ethiopia and Bangladesh, women familiar with iron supplements could reliably identify them using various cues (color, pill shape, packaging, images on packaging).	Iron-related questions were dropped after confirming recognition. Visual aid retained a diverse range of packaging (strips, bottles, boxes, loose pills) to support recognition.
**Recognition of MMS (UNIMMAP & FullCare)**	Women exposed to MMS in both countries recognized the product based on visual attributes (color, packaging, images). In Ethiopia, UNIMMAP was often received as loose pills in paper packets, plastic bags, or unsealed bottles.	The visual aid was adjusted to prominently feature loose pills and include all reported packaging types.

Dimension tested	Results	Resulting adaptation
**Recognition of Folic Acid**	Folic acid was not recognized as a distinct product by women in Ethiopia and Bangladesh; it was often mistaken for iron.	Folic acid-specific questions and visuals were removed due to low relevance/recognition.
**Recognition of Calcium**	In Bangladesh, women who had received or been exposed to calcium supplements were able to recognize them based on appearance and packaging.	Calcium visuals were retained in the aid.
**Other Products to Include**	In Ethiopia, women did not suggest additional supplements; they interpreted the visual aid as comprehensive and specific.	Dropped open-ended questions about other products and focused on assessing recognition within predefined types.
**Understanding of Product Categories vs Specific Products**	In Ethiopia & Bangladesh, women often interpreted the visual aid as a list of specific products rather than product categories. If their exact product wasn't shown, they assumed they had not taken that type of supplement.	Revised instructions and probing questions were added to encourage category-level thinking.
**Clarity of Visual Aid (Images & Text)**	Women generally found the visual aid clear and used it to identify products they had taken. Women who could read emphasized the importance of being able to read the product labels and reported that the text was clear and visible.	No changes needed. Maintained image and text size in visual aid.
**Suggestions for Visual Aid Improvement**	In Ethiopia, some women were unclear about which products were meant to be grouped together in the visual aid. No other specific suggestions for improving the font, labels, or layout were made.	A visual border was added around each product grouping to clarify categorization.

Table S3 Key: MMS = Multiple Micronutrient Supplement; UNIMMAP = United Nations International Multiple Micronutrient Antenatal Preparation

Table S4: Bangladesh Calcium Questions Used in One Nutrition Coverage Survey

SN	Question	Response
PP.6	During your pregnancy with [INSERT NAME IN PP.0], were you given, or did you buy any tablets that contain calcium?<br><br>INSTRUCTION: SHOW VISUAL AID OF CALCIUM<br><br>READ ALOUD: PLEASE THINK ABOUT THESE AND SIMILAR PRODUCTS; THE PICTURES ARE JUST EXAMPLES	01. No>>skip to PP.13<br>02. Yes<br>98. Don't know>>skip to PP.13
PP.7	How many months pregnant were you when you first started taking tablets that contain calcium during your pregnancy with [INSERT NAME IN PP.0]?<br><br>INSTRUCTION: SHOW VISUAL AID OF CALCIUM	(Record no. months)<br>(0-9 months<br>98. Don't know
PP.8	During your pregnancy with [INSERT NAME IN PP.0], how many **months** did you take tablets that contain calcium?<br><br>INSTRUCTION: SHOW VISUAL AID OF CALCIUM	(Record no. months)<br>(0-9 months<br>98. Don't know
PP.9	During your pregnancy with [INSERT NAME IN PP.0], how many days a **MONTH** did you usually take tablets that contain calcium?	(Record no. days)

	INSTRUCTION: SHOW VISUAL AID<br><br>INSTRUCTION: IF THE WOMAN DOES NOT REMEMBER, PROBE FOR THE APPROXIMATE NUMBER OF DAYS, E.G., BY ASKING HOW MANY MONTHS PREGNANT SHE WAS WHEN SHE BEGAN TAKING THE TABLETS AND WHETHER SHE TOOK THE TABLETS EVERY DAY AFTER THAT.	(0-31 days)<br>98. Don’t know
PP.10	Where did you get tablets that contain calcium from during your pregnancy with [INSERT NAME IN PP.0]?<br>[PROBE: ANYWHERE ELSE?]<br><br>[MULTI SELECT]<br><br>INSTRUCTION: SHOW VISUAL AID OF CALCIUM	01. Medical college hospital<br>02. Specialized govt hospital<br>03. District hospital<br>04. MCWC<br>05. Upazila health complex<br>06. UH & family welfare center<br>07. Community clinic<br>08. Satellite Clinic/EPIU outreach<br>09. NGO sector (NGO clinics, NGO worker)<br>10. Blue star provider<br>11. Private medical college<br>12. Private hospital<br>13. Private clinic<br>14. Qualified doctor chamber<br>15. Unqualified doctor chamber<br>16. Pharmacy<br>17. Retail shop

		18. Community health worker<br>19. Family/Friend/Neighbor<br>88. Others (specify)<br>98. Don’t know

Table S4 Key: PP = xxx; SN = xxx

Text S1: Freelisting Semi-Structured Interview, Bangladesh

Tool S1

(Site Inventory)

Maternal Micronutrient Light Touch FR

MODULE 1: IDENTIFICATION			
S.N.	QUESTION	RESPONSE CODE	
101	সাক্ষাৎকারের তারিখ Date	[__][__] [__][__] [__][__][__][__]<br>DAY MONTH YEAR<br>দিন মাস বছর	
102	পরিদর্শন শুরুর সময় Visit Start Time	[__][__] [__][__]<br>HR MIN<br>ঘন্টা মিনিট	
103	পরিদর্শন শেষের সময় Visit End Time	[__][__] [__][__]<br>HR MIN<br>ঘন্টা মিনিট	
104	সুপারভাইজারের নাম ও আইডি Supervisor name and ID	___________________________________	[__][__]
105	সাক্ষাৎকার গ্রহণকারীর নাম ও আইডি Interviewer name and ID	___________________________________	[__][__]
106	বিভাগ Division	ঢাকা Dhaka = 01<br>সিলেট Sylhet = 02	[__][__]
107	জেলা District	ঢাকা Dhaka = 01<br>সিলেট Sylhet = 02	[__][__]
108	উপজেলা Upazila	ঢাকা সদর Dhaka Sadar = 01<br>সাভার Savar = 02<br>সিলেট সদর Sylhet Sadar = 03<br>গোয়াইনঘাট Gowainghat = 04	[__][__]
109	গ্রামের নাম Name of village		
110	স্থানের ধরণ Location type (PRIMARY)	স্বাস্থ্যকেন্দ্র Health Facility = 01	[__][__]
		দোকান/ ফার্মেসি Retail / pharmacy = 02	
		কমিউনিটি স্বাস্থ্যকর্মী (ব্রাক) CHW (BRAC) = 03	
		অন্যান্য Other = 04	
111	সেবাকেন্দ্রের ধরণ Facility Type	কমিউনিটি ক্লিনিক CC = 01	[__][__]
		উপজেলা স্বাস্থ্য ও পরিবার কল্যান কেন্দ্র/ পরিবার কল্যান কেন্দ্র UH & FWC/FWC = 02	
		সুর্যের হাসি Surjer Hashi = 03	
		এস এম সি নীল তারকা ক্লিনিক SMC Blue Star Clinic=04	
		মডেল ফার্মেসি Model Pharmcy = 05	
		রেজিস্ট্রার্ড ফার্মেসি Registered Pharmacy=06	
		রেজিস্ট্রার্ড ফার্মেসি নয় Non-registered Pharmacy=07	
		অন্যান্য Other = 08	
	দোকানের ধরণ Retail Type	সুপারশপ Supershop =01	

112		খুচরা দোকান (দোকানে প্রবেশ করতে পারে তবে বিক্রেতা সরবরাহ করতে পারে) Retail shop (can enter shop but may be served) = 02	
		কিয়স্ক (শুধুমাত্র কাউন্টার, নিজে নেয়া যাবে এমন নয়) Kiosk (only counter, no self-service) = 03	
		বাজারের দোকান (অস্থায়ী দোকান) অস্থায়ী খোলা বাজারে (সাপ্তাহিক হাট) Market stall (non-permanent stall) on non-permanent open-air market (e.g. weekly) = 04	
		স্থায়ী বাজারের দোকান Market stall at permanent market = 05	
		গেইন পার্টনার GAIN PARTNER = 06	
		অন্যান্য (নির্দিষ্ট করন) Other (specify)	
113	সম্মতি: আপনি কি এই সাক্ষাৎকারে অংশগ্রহণ করতে সম্মত? CONSENT: Do you agree to patricipate in this interview?	হ্যা Yes = 01<br>না No = 02	[__][__]
114	সম্মতি: আপনি কি এই গবেষণার জন্য বিতরণ করা বা বিক্রি করা পণ্যগুলির ছবি তুলতে অনুমতি দিতে সম্মত? CONSENT: Do you agree to allowing photographs of products being distributed or sold for this study?	হ্যা Yes = 01<br>না No = 02	[__][__]
note 1	আমি সাক্ষাত্কার শুরু করার আগে আমি আপনাকে আপনার সম্পর্কে কয়েকটি প্রশ্ন জিজ্ঞাসা করব। আপনার শেয়ার করা যেকোনো তথ্য গোপনীয় থাকবে।<br>I will ask you a few questions about yourself before I start the interview. Please note that any information shared is confidential.		

MODULE 2: DEMOGRAPHIC INFORMATION			
**S.N.**	**QUESTION**	**RESPONSE CODE**	
201	স্বাস্থ্য কেন্দ্রের বা দোকানের নাম?<br><br>নির্দেশনাঃ স্বাস্থ্য কেন্দ্র বা দোকানের নাম জিজ্ঞেস করবেন না। স্বাস্থ্য কেন্দ্রে বা দোকানে প্রবেশের আগে এর নাম লিখে ফেলুন। নাম না থাকলে ঘর ফাকা রাখুন। Name of health facility or retail location?<br><br>Interviewer Instruction (do not ask employee, write shop name before entering the shop). If there no name for the shop leave it blank		
202	উত্তরদাতার পদবী<br>Respondent role/position?	ফার্মাসিস্ট Pharmacist = 01	[__][__]
		খুচরা দোকানদার Retail staff = 02	
		স্বাস্থ্যকর্মী Clinical provider = 03	
203	উত্তরদাতা যা বলেছেন ঠিক তা লিখুন Write down exactly what the respondent says for Q202		
204	কতমাস ধরে আপনি এখানে কাজ করেন?<br>How long have you worked here? (In months)	মাস MONTHS<br>জানিনা Don't know = 98	[__][__]<br>MONTHS
205	আপনি কি কোন ভিটামিন বা পুষ্টি সম্পূরক বিতরণ বা বিক্রি করে থাকেন?<br>Do you distribute or sell any vitamins or nutritional supplements to pregnant women?	হ্যা Yes = 01<br>না No = 02	[__][__]

206	দোকান বা স্বাস্থ্য কেন্দ্রের বাইরের ছবি<br>Take photo(s) of exterior of shop		

**MODULE 3: VITAMINS & NUTRITIONAL SUPPLEMENTS**			
**Interview instruction:** We want the FORMAL name of product here			
**S.N.**	**QUESTION**	**RESPONSE CODE**	
301	এই স্থান থেকে গর্ভবতী মহিলাদের জন্য কোন ধরনের ভিটামিন বা পুষ্টি সম্পূরক (১) বিতরণ করা হয় বা গর্ভবতী মহিলারা ক্রয় করে থাকেন?<br>What vitamins or nutrition supplements are distributed to or purchased by pregnant women at this location? (prod1)	ধরন ১ Freelist item 1 (item1)	
		ধরন ২ Freelist item 2 (item2)	
		ধরন ৩ Freelist item 3 (item3)	
		ধরন ৪ Freelist item 4 (item4)	
		ধরন ৫ Freelist item 5 (item5)	
		ধরন ৬ Freelist item 6 (item6)	
		ধরন ৭ Freelist item 7 (item7)	
		ধরন ৮ Freelist item 8 (item8)	
		ধরন ৯ Freelist item 9 (item9)	
		ধরন ১০ Freelist item 10 (item10)	
		ধরন ১১ Freelist item 11 (item11)	
		ধরন ১২ Freelist item 12(item12)	
		ধরন ১৩ Freelist item 13(item13)	
		ধরন ১৪ Freelist item 14(item14)	
		ধরন ১৫ Freelist item 15(item15)	

		ধরন ১৬ Freelist item 1(item1)	
		ধরন ১৭ Freelist item 17(item17)	
		ধরন ১৮ Freelist item 18(item18)	
		ধরন ১৯ Freelist item 19(item19)	
		ধরন ২০ Freelist item 20(item20)	

302	এই পণ্যগুলির মধ্যে কোনটি সবচেয়ে জনপ্রিয়? Which one of these products is the most popular?	
303	বেশিরভাগ গর্ভবতী মহিলারাই কি এই সাপ্লিমেন্টটি কিনে থাকেন নাকি নির্দিষ্ট কিছু গর্ভবতী মহিলারা এটা কিনেন?<br>Is it purchased by many/most pregnant women or a sub-group of pregnant women?	

জেলা District	[__][__]
উপজেলা Upazila	[__][__]
স্থানের ধরণ Location	[__][__]
সেবাকেন্দ্রের ধরণ Facility Type	[__][__]
দোকানের ধরণ Retail Type	[__][__]
Freelist Item	[__][__]

301_pro 1.1	ভিটামিন বা পুষ্টি সম্পূরক (১) এর ধরণ কি? What is the form of product?	ক্যাপসুল Capsule = 01<br>ট্যাবলেট Tablet = 02<br>সিরাপ Liquid = 03<br>গুড়া Powder = 04<br>অন্যান্য Other (specify) = 05	[__][__]
1.2	ভিটামিন বা পুষ্টি সম্পূরক (১) এ কোন কোন ভিটামিন থাকে? What vitamins does it contain?		
1.3	এই ভিটামিন বা পুষ্টি সম্পূরক (১) গুলো কিনতে এসে আপনার গ্রাহক/ক্রেতারা কি নাম/শব্দ ব্যবহার করেন? তারা কোন নির্দিষ্ট শব্দ ব্যবহার করে? (ইন্টারভিউয়ার এর জন্য নির্দেশনা: যখন কেউ আপনার দোকানে আসে, তারা কীভাবে এই পণ্যটি চায়?)<br>What (informal) names/terms do your clients/customers use for this product? What is the specific word they use?<br>(Instruction for the interviewer: When someone comes to your shop, how do they ask for this product?)		
1.4	আপনি যখন আপনার গ্রাহক/ক্রেতার সাথে এই সাপ্লিমেন্টটি (১) নিয়ে কথা বলেন তখন আপনি কোন নাম/শব্দ ব্যবহার করেন?<br>What do name/terms do you use for this product when talking to your clients/customers?		
1.5	ভিটামিন বা পুষ্টি সম্পূরক (১) গ্রাহক/ক্রেতার কাছে বিতরণের সময় কীভাবে প্যাকেজ করা হয়?<br>How is this packaged when distributed to client/customer?	মূল প্যাকেজিংঃ বক্স Original packaging: box = 01<br>মূল প্যাকেজিংঃ পূর্ণ ব্লিস্টার/ওষুধের পাতা Original packaging: Full blister pack = 02<br>মূল প্যাকেজিংঃ আংশিক ব্লিস্টার/ওষুধের পাতা Original packaging: Partial blister pack = 03<br>মূল প্যাকেজিংঃ বোতল Original packaging: bottle = 04<br>খোলা প্যাকেটঃ ছোট প্যাকেট/খাম Repackaged: sachet/envelope = 05<br><br>অন্যান্য (নির্দিষ্ট করুন) Other [SPECIFY] = 06	[__][__]
1.6	আর কিভাবে ভিটামিন বা পুষ্টি সম্পূরক (১) প্যাকেজিং করা হয়? How is this packaged? (Additional comments)		
1.7	এই ভিটামিন বা পুষ্টি সম্পূরক (১) পেতে কি কোন স্বাস্থ্যকর্মীর প্রেসক্রিপশন প্রয়োজন হয়?	হ্যা, সবসময় প্রেসক্রিপশন প্রয়োজন Yes always requires prescription = 01<br>মাঝেমাঝে/ এটা নির্ভর করে	[__][__]

	Does this product require a prescription from a health care provider?	Sometimes / it depends (explain) = 02<br>না, প্রেসক্রিপশনের প্রয়োজন নেই<br>No does not require a prescription = 03
1.7.1	যদি 2, ব্যাখ্যা করুন If Option 2 for Q1.7, Please explain	
1.8	একজন ক্রেতা /গ্রাহক এই পণ্যটি সাধারণত কি পরিমাণ চেয়ে থাকে? What is the general quantity of this product a client/customer asks for?<br>যেমনঃ #ট্যাবলেট, #ট্যাবলেটের বক্স ইত্যাদি Number of the product Example: # tablets, # boxes with X tabelets each, etc)	
1.8.1	প্রতিবার ভিজিটের সময় সাধারণত গ্রাহক/ক্রেতাকে কি পরিমাণ ভিটামিন বা পুষ্টি সম্পূরক (১) বিতরণ করা হয়?<br>What quantity of the product is usually provided to the client/customer during single visit?<br>যেমনঃ #ট্যাবলেট, #ট্যাবলেটের বক্স ইত্যাদি<br>Example: # tablets, # boxes with X tabelets each, etc)	
1.9	কারা সাধারণত এই ভিটামিন বা পুষ্টি সম্পূরক (১) গ্রহণ/কিনে থাকেন?<br>(নির্দিষ্ট জনগোষ্ঠীর বিবরণঃ যারা প্রাইভেট হাসপাতাল/চেম্বার থেকে সেবা নেয়; উচ্চ আয় ইত্যাদি)<br>Who tends to receive/purchase this product?<br>(describe subgroup - e.g. private care, higher income, etc)	
1.10	এটা কি সাধারণত নির্দিষ্ট কোন সম্প্রদায়ের মায়েদের জন্য সুপারিশ করা হয়ে থাকেন?<br>(নির্দিষ্ট জনগোষ্ঠীর বিবরণঃ যারা প্রাইভেট হাসপাতাল/চেম্বার থেকে সেবা নেয়; উচ্চ আয় ইত্যাদি) কেন?<br>Are you recommend this to a specific group of women? (describe subgroup - e.g. private care, higher income, etc) why?	
1.11	ভিটামিন বা পুষ্টি সম্পূরক (১) সম্পর্কে অন্যান্য কোন মন্তব্য<br>Other enumerator comments about product	
1.12	ভিটামিন বা পুষ্টি সম্পূরক (১) এর ছবিঃ বক্সের সামনের অংশের ছবি Photo of product: Primary package (side A)<br>Primary package example: box that contains blisters, original bottle,	
1.13	ভিটামিন বা পুষ্টি সম্পূরক (১) এর ছবিঃ বক্সের পেছনের অংশের ছবি Photo of product: Primary package (side B)<br>Primary package example: box that contains blisters, original bottle,	
1.14	ভিটামিন বা পুষ্টি সম্পূরক (১) এর ছবিঃ বক্সের ভেতরের ব্লিষ্টারের সামনের অংশের ছবি Photo of product: Secondary Package (side A)<br>Secondary package example: blister packs within a box; repackaging example	

1.1<br>5	ভিটামিন বা পুষ্টি সম্পূরক (১) এর ছবিঃ বক্সের ভেতরের ব্লিষ্টারের পেছনের অংশের ছবি<br>Photo of product: Secondary Package (side B)<br>Secondary package example: blister packs within a box; repackaging example
1.1<br>6	ভিটামিন বা পুষ্টি সম্পূরক (১) এর ছবিঃ অন্যান্য বক্স, ব্লিষ্টার আর ওষুধের একসাথে ছবি Photo of product: Other<br>Any other useful photos

জেলা District	[__][__]
উপজেলা Upazila	[__][__]
স্থানের ধরণ Location	[__][__]
স্বাস্থ্যকেন্দ্রের ধরণ Facility Type	[__][__]
দোকানের ধরণ Retail Type	[__][__]
Freelist	[__][__]

MODULE 4: OTHER PRODUCTS (PILLS/TABLETS/SYRUP) FOR PREGNANT WOMEN			
**S.N.**	**QUESTION**		**RESPONSE CODE**
401_med	এই স্থান থেকে ভিটামিন বা পুষ্টি সম্পূরক এর মত অন্য কোন ধরনের ওষুধ (১) কি গর্ভবতী মহিলাদের বিতরণ করা হয় বা গর্ভবতী মহিলারা ক্রয় করে থাকেন? (গর্ভকালীন জটিলতার জন্য নয়।)	ধরন ১ Freelist item 1 (item1)	
		ধরন ২ Freelist item 2 (item2)	
		ধরন ৩ Freelist item 3 (item3)	
		ধরন ৪ Freelist item 4 (item4)	
		ধরন ৫ Freelist item 5 (item5)	
		ধরন ৬ Freelist item 6 (item6)	

	Are there medicines or other products that have similar appearance to vitamins or nutrition supplements that are distributed to or purchase by pregnant women at this location? (Just for pregnancy, Not for any complication)	ধরন ৭ Freelist item 7 (item7)	
		ধরন ৮ Freelist item 8 (item8)	
		ধরন ৯ Freelist item 9 (item9)	
		ধরন ১০ Freelist item 10 (item10)	
		ধরন ১১ Freelist item 11 (item11)	
		ধরন ১২ Freelist item 12(item12)	
		ধরন ১৩ Freelist item 13(item13)	
		ধরন ১৪ Freelist item 14(item14)	
		ধরন ১৫ Freelist item 15(item15)	
		ধরন ১৬ Freelist item 16(item16)	

1.1	ওষুধ (১) এর ধরণ কি? What is the form of product?	আয়রন Iron = 01<br>ফলিক এসিড Folic acid = 02<br>আয়রন ফলিক এসিড Iron Folic Acid = 03<br>ক্যালসিয়াম Calcium (+/- Vit D) = 04<br>মাল্টিভিটামিন Multivitamin (3+ vitamins) = 05<br>কৃমিনাশক বড়ি Deworming Tablets = 06<br>অন্যান্য (নির্দিষ্ট করুন) Other [SPECIFY] = 07	
1.2	ওষুধ (১) এ কোন কোন ভিটামিন থাকে? What vitamins does it contain?		
1.3	ওষুধ (১) কিনতে এসে আপনার গ্রাহক/ক্রেতারা কি নাম/শব্দ ব্যবহার করেন? What (informal) names/terms do your clients/customers use for this product?		
1.4	আপনি যখন আপনার গ্রাহক/ক্রেতার সাথে এই ওষুধ (১) নিয়ে কথা বলেন তখন আপনি কোন নাম/শব্দ		

	ব্যবহার করেন? (যখন কেউ আপনার দোকানে আসে, তারা কীভাবে এই পণ্যটি চায়?)<br>What do name/terms do you use for this product when talking to your clients/customers? (Instruction for the interviewr: When someone comes to your shop, how do they ask for this product?)		
1.5	ওষুধ (১) গ্রাহক/ক্রেতার কাছে বিতরণের সময় কীভাবে প্যাকেজ করা হয়?<br>How is this packaged when distributed to client/customer?	মূল প্যাকেজিংঃ বক্স Original packaging: box = 01<br>মূল প্যাকেজিংঃ পূর্ণ ব্লিস্টার/ওষুধের পাতা Original packaging: Full blister pack = 02<br>মূল প্যাকেজিংঃ আংশিক ব্লিস্টার/ওষুধের পাতা Original packaging: Partial blister pack = 03<br>মূল প্যাকেজিংঃ বোতল Original packaging: bottle = 04<br>খোলা প্যাকেটঃ ছোট প্যাকেট/খাম Repackaged: sachet/envelope = 05<br><br>অন্যান্য (নির্দিষ্ট করুন) Other [SPECIFY] = 06	[__][__]
1.6	আর কিভাবে ওষুধ (১) প্যাকেজিং করা হয়? How is this packaged? (Additional comments)		
1.7	এই ওষুধ (১) পেতে কি কোন স্বাস্থ্যকর্মীর প্রেসক্রিপশন প্রয়োজন হয়?<br>Does this product require a prescription from a health care provider?	হ্যা, সবসময় প্রেসক্রিপশন প্রয়োজন<br>Yes always requires prescription = 01<br>মাঝেমাঝে/ এটা নির্ভর করে<br>Sometimes / it depends (explain) = 02<br>না, প্রেসক্রিপশনের প্রয়োজন নেই<br>No does not require a prescription = 03	[__][__]
1.7.1	যদি 2, ব্যাখ্যা করুন If Option 2, Explain		
1.8	সাধারণত গ্রাহক/ক্রেতারা যখন ওষুধ নিতে আসেন তখন তারা কি পরিমাণ চেয়ে থাকেন? What is the general quantity of this product a client/customer asks for?<br>Probe: Number of the product Example: # tablets, # boxes with X tabelets each, etc)		
1.9	প্রতিবার ভিজিটের সময় সাধারণত গ্রাহক/ক্রেতাকে কি পরিমাণ ভিটামিন বা পুষ্টি সম্পূরক (১) বিতরণ করা হয়?<br>What quantity of the product is usually provided to the client/customer during single visit?<br>যেমনঃ #ট্যাবলেট, #ট্যাবলেটের বক্স ইত্যাদি		[__][__]

	Example: # tablets, # boxes with X tabelets each, etc)		
1.10	কারা সাধারণত এই ভিটামিন বা পুষ্টি সম্পূরক (১) গ্রহণ/কিনে থাকেন? (নির্দিষ্ট জনগোষ্টীর বিবরণঃ যারা প্রাইভেট হাসপাতাল/চেম্বার থেকে সেবা নেয়; উচ্চ আয় ইত্যাদি)<br>Who tends to receive/purchase this product? (describe subgroup - e.g. private care, higher income, etc)		
1.11	ওষুধ (১) সম্পর্কে অন্যান্য কোন মন্তব্য<br>Other enumerator comments about product		
1.12	ওষুধ (১) এর ছবিঃ বক্সের সামনের অংশের ছবি<br>Photo of product: Primary package (side A)<br>Primary package example: box that contains blisters, original bottle,		
1.13	ওষুধ (১) এর ছবিঃ বক্সের পেছনের অংশের ছবি<br>Photo of product: Primary package (side B)<br>Primary package example: box that contains blisters, original bottle,		
1.14	ওষুধ (১) এর ছবিঃ বক্সের ভেতরের ব্লিষ্টারের সামনের অংশের ছবি<br>Photo of product: Secondary Package (side A)<br>Secondary package example: blister packs within a box; repackaging example		
1.15	ওষুধ (১) এর ছবিঃ বক্সের ভেতরের ব্লিষ্টারের পেছনের অংশের ছবি<br>Photo of product: Secondary Package (side B)<br>Secondary package example: blister packs within a box; repackaging example		
1.16	ওষুধ (১) এর ছবিঃ অন্যান্য বক্স, ব্লিষ্টার আর ওষুধের একসাথে ছবি<br>Photo of product: Other<br>Any other useful photos		

Text S2: Landscaping Guide, Bangladesh

Tool S2
(KII Pregnant Women)
Maternal Micronutrient Light Touch FR

Module 1: Identification		
**SN**	**Question**	**Response Code**

101	সাক্ষাৎকারের তারিখ<br>Date	[ ][ ] [ ][ ] [ ][ ][ ][ ]<br>DAY MONTH YEAR<br>দিন মাস বছর	
102	পরিদর্শন শুরুর সময়<br>Visit Start Time	[ ][ ] [ ][ ]<br>HR MIN<br>ঘন্টা মিনিট	
103	পরিদর্শন শেষের সময়<br>Visit End Time	[ ][ ] [ ][ ]<br>HR MIN<br>ঘন্টা মিনিট	
104	সুপারভাইজারের নাম ও আইডি Supervisor name and ID		[ ][ ]
105	সাক্ষাৎকার গ্রহণকারীর নাম ও আইডি<br>Interviewer name and ID		[ ][ ]
106	বিভাগ Division	Dhaka= 01<br>Sylhet = 02	[ ][ ]
107	জেলা District	Dhaka= 01<br>Sylhet = 02	[ ][ ]
108	উপজেলা Upazila		
109	গ্রামের নাম Name of village	____________________	
110	উত্তরদাতার নাম/আইডি<br>Respondent Name/ID	____________________	[ ][ ]
111	সম্মতি: আপনি কি এই সাক্ষাৎকারে অংশগ্রহণ করতে সম্মত?<br>CONSENT: Do you agree to participate in this interview?	হ্যাঁ Yes = 01<br>না No = 02	[ ][ ]

Consent Form
According to Tool C

MODULE 2: DEMOGRAPHIC INFORMATION			
**সাক্ষাৎকারগ্রহণকারীর জন্য নির্দেশনা (জোরে পড়ুন): আমি সাক্ষাৎকার শুরু করার আগে আপনাকে আপনার সম্পর্কে কয়েকটি প্রশ্ন জিজ্ঞাসা করব। অনুগ্রহ করে মনে রাখবেন যে আপনার শেয়ার করা যেকোনো তথ্য গোপন রাখা হবে।** **Interviewer Instruction (Read aloud)**: I will ask you a few questions about yourself before I start the interview. Please note that any information shared is confidential.			
**S.N.**	**QUESTION**	**RESPONSE CODE**	
201	উত্তরদাতার নাম Respondent name		
202	আপনার সর্বশেষ জন্মদিনে আপনার বয়স কত ছিল? How old were you at your last birthday?	বয়স AGE IN YEARS<br>জানি না Don't know = 98	[ ][ ]
203	আপনি শিক্ষার সর্বোচ্চ কোন শ্রেণি <u>সম্পূর্ণ</u> করেছেন? What is the highest level of education you attended?	অপ্রাতিষ্ঠানিক শিক্ষা No schooling = 01<br>স্বশিক্ষিত Literate without schooling = 02<br>প্রাক- প্রাথমিক Pre-primary = 03<br>প্রাথমিক সম্পন্ন Primary (Class 1-5 completed) = 04<br>মাধ্যমিক সম্পন্ন Secondary (Class 6-10 completed) = 05<br>উচ্চ মাধ্যমিক সম্পন্ন Higher Secondary (Class 11-12 completed) = 07<br>কারিগরি/ বৃত্তিমূলক প্রশিক্ষণ Technical/Vocational training (TVET) = 08<br>স্নাতক ডিগ্রী সম্পন্ন Undergraduate degree completed = 09<br>স্নাতকোত্তর ডিগ্রী সম্পন্ন Postgrad degree completed = 10<br>প্রযোজ্য নয় Not applicable = 11<br>জানি না Don't know = 98	[ ][ ]
204	আপনি কতবার সন্তান প্রসব করেছেন? How		[ ][ ]

	many times have you given birth?		
205	আপনি এখন কত মাসের গর্ভবতী? How many months pregnant are you now?		[ ][ ]
206	আপনি কি এই গর্ভাবস্থায় গর্ভকালীন সেবা পেয়েছেন? Have you received antenatal care during this pregnancy?	হ্যাঁ Yes = 01<br>না No = 02<br>জানি না Don't know = 98	[ ][ ]
207	এই গর্ভাবস্থার জন্য আপনি কোথায় গর্ভকালীন সেবা পেয়েছেন? অন্য কোথাও? Where did you receive antenatal care for this pregnancy? Anywhere else?	বাড়ি Home=01<br>জেলা হাসপাতাল District hospital=02<br>সরকারি হাসপাতাল Government hospital=03<br>মেডিকেল কলেজ হাসপাতাল Medical College Hospital=04<br>মা ও শিশু কল্যাণ কেন্দ্র Maternal and Child Welfare Centre=05<br>উপজেলা স্বাস্থ্য কমপ্লেক্স Upazila Health Complex=06<br>স্বাস্থ্য ও পরিবার কল্যাণ কেন্দ্র Health and family Welfare Centre=07<br>স্যাটেলাইট ক্লিনিক/ইপিআই আউটরিচ Satellite Clinic/EPI Outreach=08<br>কমিউনিটি ক্লিনিক Community clinic=09<br>এনজিও ক্লিনিক NGO Static Clinic=10<br>এনজিও স্যাটেলাইট ক্লিনিক NGO Satellite Clinic=11<br>প্রাইভেট মেডিকেল কলেজ হাসপাতাল Private Medical College Hospital=12<br>প্রাইভেট হাসপাতাল এবং ক্লিনিক Private Hospital and Clinic=13<br>এমবিবিএস ডাক্তার Qualified Doctor=14<br>সনাতনী ডাক্তার Traditional Doctor=15<br>ফার্মেসি Pharmacy=16<br>অন্যান্য, (নির্দিষ্ট করুন) Other, (Specify)----	[ ][ ]
208	এই গর্ভাবস্থার আপনি কত মাস গর্ভকালীন সময়ে প্রথম গর্ভকালীন সেবা পেয়েছিলেন?	৩ মাসের কম <3 months =01<br>৩-৬ মাস 3-6 months =02<br>৭-৯ মাস 7-9 months =03<br>জানি না Don't know = 98	[ ][ ]

	How many months pregnant were you when you first received antenatal care for this pregnancy?		
209	এই গর্ভাবস্থায় আপনি এ পর্যন্ত কতবার গর্ভকালীন সেবা পেয়েছেন? How many times have you received antenatal care so far during this pregnancy?		[ ][ ]

Module 3: Demographic Information				
ন্নির্দেশনা (উচ্চস্বরে পড়ুন): এ পর্যায়ে মহিলাটি স্থানীয় ভাষায় যা কিছু বলেন, সবকিছু লিখে রাখুন। ইংরেজিতে ব্যাখ্যা বা অনুবাদ করবেন না। মহিলাদের যতটা সম্ভব সাপ্লিমেন্টের নাম তালিকাভুক্ত করতে উৎসাহিত করুন এবং বারবার জিজ্ঞাসা করুন, "আর কিছু?" যখন মহিলাটি সম্পূর্ণভাবে তাদের তালিকা শেষ করবেন, তখন আপনি প্রতিটি আইটেমের জন্য ফলো-আপ প্রশ্ন করতে পারেন।<br>**Interviewer instructions (Read aloud)**: Write down everything the woman says in local language at this stage do not clarify or translate to English. Encourage women to list as many products as possible, continue to probe for, "anything else" Once the woman is completely done with their free list you can ask the follow-up questions for each item listed.				
**S.N.**	**QUESTION**	**RESPONSE CODE**	**Item Name** (Exact same words that used by respondents)	**Facility/Retail** (Exact same words that used by respondents)
301	আপনার সম্প্রদায়ের একজন মহিলা যখন গর্ভবতী হন, তখন তাদের স্বাস্থ্য কেন্দ্র থেকে কি কি সাপ্লিমেন্ট প্রদান করা হয়? গর্ভবতী মহিলারা ফার্মেসি থেকে কোন সাপ্লিমেন্টগুলো কেনেন?<br><br>When a woman in your community is pregnant, what products are they given at a health facility? What products do pregnant women	ধরন ১ Freelist item 1 (item1)		
		ধরন ২ Freelist item 2 (item2)		
		ধরন ৩ Freelist item 3 (item3)		
		ধরন ৪ Freelist item 4 (item4)		
		ধরন ৫ Freelist item 5 (item5)		
		ধরন ৬ Freelist item 6 (item6)		
		ধরন ৭ Freelist item 7 (item7)		
		ধরন ৮ Freelist item 8 (item8)		
		ধরন ৯ Freelist item 9 (item9)		
		ধরন ১০ Freelist item 10 (item10)		
		ধরন ১১ Freelist item 11 (item11)		
		ধরন ১২ Freelist item 12(item12)		
		ধরন ১৩ Freelist item 13(item13)		

জেলা District	[ ][ ]
উপজেলা Upazila	[ ][ ]
স্থানের ধরণ Location	[ ][ ]
উত্তরদাতার আইডি Respondent ID	[ ][ ]
Freelist	[ ][ ]

	purchase at a pharmacy?	ধরন ১৪ Freelist item 14(item14)		
		ধরন ১৫ Freelist item 15(item15)		

301 item1	ধরন ১ Freelist item 1	
301 item1.1	(ধরন ১ নাম) কিসের জন্য ব্যবহার করা হয়? What is this product for? (Exact same words that used by respondents)	
301 item1.2	আপনার এলাকায় (ধরন ১ নাম) এর আর কোন নাম আছে যেটা মানুষ সাধারণত ব্যবহার করে থাকেন? প্রোবঃ প্রচলিত নাম, অপ্রচলিত নাম Are there other names or terms that people in your locality use for this	

	product? Probe: formal, informal	
301 item1.3	(ধরন ১ নাম) দেখতে কেমন একটু বর্ণনা করুন<br>Please describe what this product looks like	
301 item1.4	যখন আপনি (ধরন ১ নাম) পেয়েছেন বা কিনেছেন এর প্যাকেজিং কেমন ছিল? বর্ননা করতে পারবেন? প্রোবঃ বক্স, বোতল, স্ট্রিপ, পিল, প্যাকেজিং এর গায়ের লেখা পড়ে ইত্যাদি<br>Can you describe the packaging when you received or purchased this product comes in?<br><br>Probe: Any other packaging?	

	Probe: Color, Shape, Text/Words, Logos etc.	
301 item1.5	আপনি কিভাবে অন্যান্য সাপ্লিমেন্ট/ওষুধ এর মধ্য থেকে [পণ্যের নাম] চিনতে পারেন?<br><br>প্রোবঃ এর প্যাকেজিং দ্বারা, বক্স, বোতল, স্ট্রিপ, পিল, প্যাকেজিং এর গায়ের লেখা পড়ে ইত্যাদি<br><br>How are you able to recognize [product name] from other products? Probe: by its packaging, box, bottle, strips, pills, reading the packaging, etc.	
301 item1.6	আপনি কোন কোন জায়গা থেকে (ধরন ১ নাম) পেতে বা কিনতে পারেন?<br><br>What are the places you can get or purchase	

	this product from?	
301 item1.7	(ধরন ১ নাম) আপনার সম্প্রদায়ের গর্ভবতী মহিলাদের যখন বিতরণ করা হয় তখন এর প্যাকেজিং কেমন থাকে?<br><br>What does the packaging look like when [product name] is distributed to women in your community?	
301 item1.8	পণ্যের একটি ছবি তুলুন Take a picture of the product	

Module 4: Additional Questions		
**SN**	**Question**	**Response Code**
401 a	(আইএফএ এর ছবি দেখান) জিজ্ঞাসা করুন: এই ধরনের পণ্যকে আপনি কী নামে চিনেন? (Show image with IFA examples) ASK: What do you call this type of product?	
ইন্টারভিউয়ারের জন্য নির্দেশনা: আইএফএ এর ছবি দেখান INSTRUCTION TO INTERVIEWER: Show image of IFA example		
402 a	এই গর্ভাবস্থায় আপনি কি কোন [পণ্যের নাম] পেয়েছেন বা কিনেছেন?During this pregnancy have you received or purchased any [product name] ?	
402 b	402 a যদি হ্যাঁ হয়, আপনি গত মাসে কত দিন [পণ্যের নাম] খেয়েছেন?If yes to 402 a. How many days did you take [product name] in the last month?	
402 c	402 a যদি হ্যাঁ হয়, গত সপ্তাহে আপনি কত দিন [পণ্যের নাম] খেয়েছেন? If yes to 402 a. How many days did you take [product name] in the last week?	
402 d	402 a যদি হ্যাঁ হয়, যখন আপনার কাছে [পণ্যের নাম] সরবরাহ থাকে আপনি সাধারণত প্রতি সপ্তাহে কত দিন এটি গ্রহণ করেন? If yes to 402 a. When you have a supply of [product name] how many days per week do you usually take it?	
402 e	402 a যদি হ্যাঁ হয়, গত মাসে, আপনি বলেছিলেন যে আপনি [402 b] দিনের জন্য এই পণ্যটি খেয়েছেন; আপনি কিভাবে [402 b] দিনের উত্তরটি দিয়েছেন? If yes to 402a.During the last MONTH, you said you took this product for [402 b] days, how did you come up with the answer [402 b] days?	
402 f	আপনি বলেছিলেন যে আপনি সাধারণত প্রতি সপ্তাহে [402 d] দিন নেন, আপনি প্রতি সপ্তাহে [402 d] দিন খাওয়ার উত্তরটি কীভাবে দিয়েছিলেন? You said you usually take this [402 d] days per week,	

	how did you come up with the answer [402 d] days per week?	
ইন্টারভিউয়ারের জন্য নির্দেশনা: প্রসবপূর্ব মাল্টিভিটামিন/এমএমএস এর ছবি দেখান INSTRUCTION TO INTERVIEWER: Show the image of prenatal multivitamin/MMS		
401 b	প্রসবপূর্ব মাল্টিভিটামিন/এমএমএস এর ছবি দেখান জিজ্ঞাসা করুন: এই ধরনের পণ্যকে আপনি কী নামে চিনেন? (Show image with prenatal multivitamin/MMS examples) ASK: What do you call this type of product?	
402 a	এই গর্ভাবস্থায় আপনি কি কোন [পণ্যের নাম] পেয়েছেন বা কিনেছেন?During this pregnancy have you received or purchased any [product name] ?	
402 b	402 a যদি হ্যাঁ হয়, আপনি গত মাসে কত দিন [পণ্যের নাম] খেয়েছেন?If yes to 402 a. How many days did you take [product name] in the last month?	
402 c	402 a যদি হ্যাঁ হয়, গত সপ্তাহে আপনি কত দিন [পণ্যের নাম] খেয়েছেন? If yes to 402a. How many days did you take [product name] in the last week?	
402 d	402 a যদি হ্যাঁ হয়, যখন আপনার কাছে [পণ্যের নাম] সরবরাহ থাকে আপনি সাধারণত প্রতি সপ্তাহে কত দিন এটি গ্রহণ করেন? If yes to 402 a. When you have a supply of [product name] how many days per week do you usually take it?	
402 e	402 a যদি হ্যাঁ হয়, গত মাসে, আপনি বলেছিলেন যে আপনি [402 b] দিনের জন্য এই পণ্যটি খেয়েছেন; আপনি কিভাবে [402 b] দিনের উত্তরটি দিয়েছেন? If yes to 402a.During the last MONTH, you said you took this product for [402 b] days, how did you come up with the answer [402 b] days?	
402 f	আপনি বলেছিলেন যে আপনি সাধারণত প্রতি সপ্তাহে [402 d] দিন নেন, আপনি প্রতি সপ্তাহে [402 d] দিন খাওয়ার উত্তরটি কীভাবে দিয়েছিলেন? You said you usually take this [402 d] days per week,	

	how did you come up with the answer [402 d] days per week?	
ইন্টারভিউয়ারের জন্য নির্দেশনা: ক্যালসিয়াম এর ছবি দেখান INSTRUCTION TO INTERVIEWER: Show the image of calcium		
401 c	ক্যালসিয়াম এর ছবি দেখান জিজ্ঞাসা করুন: এই ধরনের পণ্যকে আপনি কী নামে চিনেন? (Show image with calcium examples) ASK: What do you call this type of product?	
402 a	এই গর্ভাবস্থায় আপনি কি কোন [পণ্যের নাম] পেয়েছেন বা কিনেছেন?During this pregnancy have you received or purchased any [product name] ?	
402 b	402a যদি হ্যাঁ হয়, আপনি গত মাসে কত দিন [পণ্যের নাম] খেয়েছেন?If yes to 402a. How many days did you take [product name] in the last month?	
402 c	402a যদি হ্যাঁ হয়, গত সপ্তাহে আপনি কত দিন [পণ্যের নাম] খেয়েছেন? If yes to 402a. How many days did you take [product name] in the last week?	
402 d	402a যদি হ্যাঁ হয়, যখন আপনার কাছে [পণ্যের নাম] সরবরাহ থাকে আপনি সাধারণত প্রতি সপ্তাহে কত দিন এটি গ্রহণ করেন? If yes to 402a. When you have a supply of [product name] how many days per week do you usually take it?	
402 e	402a যদি হ্যাঁ হয়, গত মাসে, আপনি বলেছিলেন যে আপনি [402b] দিনের জন্য এই পণ্যটি খেয়েছেন; আপনি কিভাবে [402b] দিনের উত্তরটি দিয়েছেন? If yes to 402a.During the last MONTH, you said you took this product for [402b] days, how did you come up with the answer [402b] days?	
402 f	আপনি বলেছিলেন যে আপনি সাধারণত প্রতি সপ্তাহে [402 d] দিন নেন, আপনি প্রতি সপ্তাহে [402 d] দিন খাওয়ার উত্তরটি কীভাবে দিয়েছিলেন? You said you usually take this [402 d] days per week, how did you come up with the answer [402 d] days per week?	

403	মায়েরা নানারকম কারণে ভিটামিন/ সাপ্লিমেন্ট খেতে ভুলে যান। আপনি কেন আপনার গর্ভাবস্থায় ভিটামিন/সাপ্লিমেন্ট খেতে ভুলে যান? Women miss taking vitamins/supplements for many reasons. Why have you missed taking a vitamin/supplement during your pregnancy?	হ্যা Yes = 01<br>না No = 02
404	কেউ যখন আপনাকে সাপ্লিমেন্ট খেতে ভুলে গেছেন এমন প্রশ্ন করে তখন আপনি কি সংকোচবোধ করেন? Do you feel hesitant to report missing a supplement when someone asks you?	হ্যা Yes = 01<br>না No = 02
405	যদি হ্যাঁ হয় তাহলে কেন? If yes, why?	

Text S2: Landscaping Guide, Bangladesh

Tool S1
(Site Inventory)
Maternal Micronutrient Light Touch FR

**Module 1: Identification**			
**SN**	**Question**	**Response Code**	
101	সাক্ষাৎকারের তারিখ Date	[ ][ ] [ ][ ] [ ][ ][ ][ ]<br>DAY MONTH YEAR<br>দিন মাস বছর	
102	পরিদর্শন শুরুর সময়<br>Visit Start Time	[ ][ ] [ ][ ]<br>HR MIN<br>ঘন্টা মিনিট	
103	পরিদর্শন শেষের সময়<br>Visit End Time	[ ][ ] [ ][ ]<br>HR MIN<br>ঘন্টা মিনিট	
104	সুপারভাইজারের নাম ও আইডি Supervisor name and ID		[ ][ ]
105	সাক্ষাৎকার গ্রহণকারীর নাম ও আইডি Interviewer name and ID		[ ][ ]
106	বিভাগ Division	ঢাকা Dhaka = 01<br>সিলেট Sylhet = 02	[ ][ ]
107	জেলা District	ঢাকা Dhaka = 01<br>সিলেট Sylhet = 02	[ ][ ]
108	উপজেলা Upazila	ঢাকা সদর Dhaka Sadar = 01<br>সাভার Savar = 02<br>সিলেট সদর Sylhet Sadar = 03<br>গোয়াইনঘাট Gowainghat = 04	[ ][ ]
109	গ্রামের নাম Name of village		
110	স্থানের ধরণ Location type (PRIMARY)	স্বাস্থ্যকেন্দ্র Health Facility = 01	[ ][ ]
		দোকান/ ফার্মেসি Retail / pharmacy = 02	
		কমিউনিটি স্বাস্থ্যকর্মী (ব্রাক) CHW (BRAC) = 03	
		অন্যান্য Other = 04	
		কমিউনিটি ক্লিনিক CC = 01	[ ][ ]

111	সেবাকেন্দ্রের ধরণ Facility Type	উপজেলা স্বাস্থ্য ও পরিবার কল্যান কেন্দ্র/ পরিবার কল্যান কেন্দ্র UH & FWC/FWC = 02	
		সূর্যের হাসি Surjer Hashi = 03	
		এস এম সি নীল তারকা ক্লিনিক SMC Blue Star Clinic=04	
		মডেল ফার্মেসি Model Pharmcy = 05	
		রেজিস্ট্রার্ড ফার্মেসি Registered Pharmacy=06	
		রেজিস্ট্রার্ড ফার্মেসি নয় Non-registered Pharmacy=07	
		অন্যান্য Other = 08	
112	দোকানের ধরণ Retail Type	সুপারশপ Supershop =01	
		খুচরা দোকান (দোকানে প্রবেশ করতে পারে তবে বিক্রেতা সরবরাহ করতে পারে) Retail shop (can enter shop but may be served) = 02	
		কিয়স্ক (শুধুমাত্র কাউন্টার, নিজে নেয়া যাবে এমন নয়) Kiosk (only counter, no self-service) = 03	
		বাজারের দোকান (অস্থায়ী দোকান) অস্থায়ী খোলা বাজারে (সাপ্তাহিক হাট) Market stall (non-permanent stall) on non-permanent open-air market (e.g. weekly) = 04	
		স্থায়ী বাজারের দোকান Market stall at permanent market = 05	
		গেইন পার্টনার GAIN PARTNER = 06	
		অন্যান্য (নির্দিষ্ট করন) Other (specify)	
113	সম্মতি: আপনি কি এই সাক্ষাৎকারে অংশগ্রহণ করতে সম্মত? CONSENT: Do you agree to patricipate in this interview?	হ্যা Yes = 01<br>না No = 02	[ ][ ]
114	সম্মতি: আপনি কি এই গবেষণার জন্য বিতরণ করা বা বিক্রি করা পণ্যগুলির ছবি তুলতে অনুমতি দিতে সম্মত? CONSENT: Do you agree to allowing photographs	হ্যা Yes = 01<br>না No = 02	[ ][ ]

	of products being distributed or sold for this study?		
note 1	আমি সাক্ষাত্কার শুরু করার আগে আমি আপনাকে আপনার সম্পর্কে কয়েকটি প্রশ্ন জিজ্ঞাসা করব। আপনার শেয়ার করা যেকোনো তথ্য গোপনীয় থাকবে।<br>I will ask you a few questions about yourself before I start the interview. Please note that any information shared is confidential.		

Module 2: Demographic Information			
SN	Question	Response code	
201	স্বাস্থ্য কেন্দ্রের বা দোকানের নাম?<br>নির্দেশনাঃ স্বাস্থ্য কেন্দ্র বা দোকানের নাম জিজ্ঞেস করবেন না। স্বাস্থ্য কেন্দ্রে বা দোকানে প্রবেশের আগে এর নাম লিখে ফেলুন। নাম না থাকলে ঘর ফাকা রাখুন।<br>Name of health facility or retail location?<br><br>Interviewer Instruction (do not ask employee, write shop name before entering the shop). If there no name for the shop leave it blank		
202	উত্তরদাতার পদবী<br>Respondent role/position?	ফার্মাসিস্ট Pharmacist = 01	[ ][ ]
		খুচরা দোকানদার Retail staff = 02	
		স্বাস্থ্যকর্মী Clinical provider = 03	
203	উত্তরদাতা যা বলেছেন ঠিক তা লিখুন Write down exactly what the respondent says for Q202		
204	কতমাস ধরে আপনি এখানে কাজ করেন?<br>How long have you worked here? (In months)	মাস MONTHS<br>জানিনা Don't know = 98	[ ][ ]<br>MONTH<br>S
205	আপনি কি কোন ভিটামিন বা পুষ্টি সম্পূরক বিতরণ বা বিক্রি করে থাকেন?<br>Do you distribute or sell any vitamins or nutritional supplements to pregnant women?	হ্যা Yes = 01<br>না No = 02	[ ][ ]

206	দোকান বা স্বাস্থ্য কেন্দ্রের বাইরের ছবি<br>Take photo(s) of exterior of shop		

Module 3: Vitamins & Nutritional Supplements

Interview instructions: We want the FORMAL name of product here

SN	Question	Response code	
301	এই স্থান থেকে গর্ভবতী মহিলাদের জন্য কোন ধরনের ভিটামিন বা পুষ্টি সম্পূরক (১) বিতরণ করা হয় বা গর্ভবতী মহিলারা ক্রয় করে থাকেন?<br>What vitamins or nutrition supplements are distributed to or purchased by pregnant women at this location? (prod1)	ধরন ১ Freelist item 1 (item1)	
		ধরন ২ Freelist item 2 (item2)	
		ধরন ৩ Freelist item 3 (item3)	
		ধরন ৪ Freelist item 4 (item4)	
		ধরন ৫ Freelist item 5 (item5)	
		ধরন ৬ Freelist item 6 (item6)	
		ধরন ৭ Freelist item 7 (item7)	
		ধরন ৮ Freelist item 8 (item8)	
		ধরন ৯ Freelist item 9 (item9)	
		ধরন ১০ Freelist item 10 (item10)	
		ধরন ১১ Freelist item 11 (item11)	
		ধরন ১২ Freelist item 12(item12)	
		ধরন ১৩ Freelist item 13(item13)	
		ধরন ১৪ Freelist item 14(item14)	
		ধরন ১৫ Freelist item 15(item15)	
		ধরন ১৬ Freelist item 1(item1)	
		ধরন ১৭<br>Freelist item 17(item17)	
		ধরন ১৮	

		Freelist item 18(item18)	
		ধরন ১৯ Freelist item 19(item19)	
		ধরন ২০ Freelist item 20(item20)	

302	এই পণ্যগুলির মধ্যে কোনটি সবচেয়ে জনপ্রিয়? Which one of these products is the most popular?	
303	বেশিরভাগ গর্ভবতী মহিলারাই কি এই সাপ্লিমেন্টটি কিনে থাকেন নাকি নির্দিষ্ট কিছু গর্ভবতী মহিলারা এটা কিনেন? Is it purchased by many/most pregnant women or a sub-group of pregnant women?	

জেলা District	[ ][ ]
উপজেলা Upazila	[ ][ ]
স্থানের ধরণ Location	[ ][ ]
সেবাকেন্দ্রের ধরণ Facility Type	[ ][ ]
দোকানের ধরণ Retail Type	[ ][ ]

Freelist Item	[ ][ ]

30 1 pro 1.1	ভিটামিন বা পুষ্টি সম্পূরক (১) এর ধরণ কি? What is the form of product?	ক্যাপসুল Capsule = 01<br>ট্যাবলেট Tablet = 02<br>সিরাপ Liquid = 03<br>গুড়া Powder = 04<br>অন্যান্য Other (specify) = 05	[ ][ ]
1.2	ভিটামিন বা পুষ্টি সম্পূরক (১) এ কোন কোন ভিটামিন থাকে? What vitamins does it contain?		
1.3	এই ভিটামিন বা পুষ্টি সম্পূরক (১) গুলো কিনতে এসে আপনার গ্রাহক/ক্রেতারা কি নাম/শব্দ ব্যবহার করেন? তারা কোন নির্দিষ্ট শব্দ ব্যবহার করে?<br>(ইন্টারভিউয়ার এর জন্য নির্দেশনা: যখন কেউ আপনার দোকানে আসে, তারা কীভাবে এই পণ্যটি চায়?)<br>What (informal) names/terms do your clients/customers use for this product? What is the specific word they use?<br>(Instruction for the interviewer: When someone comes to your shop, how do they ask for this product?)		
1.4	আপনি যখন আপনার গ্রাহক/ক্রেতার সাথে এই সাপ্লিমেন্টটি (১) নিয়ে কথা বলেন তখন আপনি কোন নাম/শব্দ ব্যবহার করেন?<br>What do name/terms do you use for this product when talking to your clients/customers?		
1.5	ভিটামিন বা পুষ্টি সম্পূরক (১) গ্রাহক/ক্রেতার কাছে বিতরণের সময় কীভাবে প্যাকেজ করা হয়?<br>How is this packaged when distributed to client/customer?	মূল প্যাকেজিংঃ বক্স Original packaging: box = 01<br>মূল প্যাকেজিংঃ পূর্ণ ব্লিস্টার/ওষুধের পাতা Original packaging: Full blister pack = 02<br>মূল প্যাকেজিংঃ আংশিক ব্লিস্টার/ওষুধের পাতা Original packaging: Partial blister pack = 03<br>মূল প্যাকেজিংঃ বোতল Original packaging: bottle = 04	[ ][ ]

		খোলা প্যাকেটঃ ছোট প্যাকেট/খাম Repackaged: sachet/envelope = 05<br><br>অন্যান্য (নির্দিষ্ট করুন) Other [SPECIFY] = 06	
1.6	আর কিভাবে ভিটামিন বা পুষ্টি সম্পূরক (১) প্যাকেজিং করা হয়? How is this packaged? (Additional comments)		
1.7	এই ভিটামিন বা পুষ্টি সম্পূরক (১) পেতে কি কোন স্বাস্থ্যকর্মীর প্রেসক্রিপশন প্রয়োজন হয়?<br>Does this product require a prescription from a health care provider?	হ্যা, সবসময় প্রেসক্রিপশন প্রয়োজন<br>Yes always requires prescription = 01<br>মাঝেমাঝে/ এটা নির্ভর করে<br>Sometimes / it depends (explain) = 02<br>না, প্রেসক্রিপশনের প্রয়োজন নেই<br>No does not require a prescription = 03	[ ][ ]
1.7.1	যদি 2, ব্যাখ্যা করুন If Option 2 for Q1.7, Please explain		
1.8	একজন ক্রেতা /গ্রাহক এই পণ্যটি সাধারণত কি পরিমাণ চেয়ে থাকে? What is the general quantity of this product a client/customer asks for?<br>যেমনঃ #ট্যাবলেট, #ট্যাবলেটের বক্স ইত্যাদি<br>Number of the product Example: # tablets, # boxes with X tabelets each, etc)		
1.8.1	প্রতিবার ভিজিটের সময় সাধারণত গ্রাহক/ক্রেতাকে কি পরিমাণ ভিটামিন বা পুষ্টি সম্পূরক (১) বিতরণ করা হয়?<br>What quantity of the product is usually provided to the client/customer during single visit?<br>যেমনঃ #ট্যাবলেট, #ট্যাবলেটের বক্স ইত্যাদি<br>Example: # tablets, # boxes with X tabelets each, etc)		
1.9	কারা সাধারণত এই ভিটামিন বা পুষ্টি সম্পূরক (১) গ্রহণ/কিনে থাকেন?		

	(নির্দিষ্ট জনগোষ্ঠীর বিবরণঃ যারা প্রাইভেট হাসপাতাল/চেম্বার থেকে সেবা নেয়; উচ্চ আয় ইত্যাদি)<br>Who tends to receive/purchase this product? (describe subgroup - e.g. private care, higher income, etc)	
1.10	এটা কি সাধারণত নির্দিষ্ট কোন সম্প্রদায়ের মায়েদের জন্য সুপারিশ করা হয়ে থাকেন? (নির্দিষ্ট জনগোষ্ঠীর বিবরণঃ যারা প্রাইভেট হাসপাতাল/চেম্বার থেকে সেবা নেয়; উচ্চ আয় ইত্যাদি) কেন?<br>Are you recommend this to a specific group of women? (describe subgroup - e.g. private care, higher income, etc) why?	
1.11	ভিটামিন বা পুষ্টি সম্পূরক (১) সম্পর্কে অন্যান্য কোন মন্তব্য<br>Other enumerator comments about product	
1.12	ভিটামিন বা পুষ্টি সম্পূরক (১) এর ছবিঃ বক্সের সামনের অংশের ছবি Photo of product: Primary package (side A)<br>Primary package example: box that contains blisters, original bottle,	
1.13	ভিটামিন বা পুষ্টি সম্পূরক (১) এর ছবিঃ বক্সের পেছনের অংশের ছবি Photo of product: Primary package (side B)<br>Primary package example: box that contains blisters, original bottle,	
1.14	ভিটামিন বা পুষ্টি সম্পূরক (১) এর ছবিঃ বক্সের ভেতরের ব্লিষ্টারের সামনের অংশের ছবি Photo of product: Secondary Package (side A)<br>Secondary package example: blister packs within a box; repackaging example	
1.15	ভিটামিন বা পুষ্টি সম্পূরক (১) এর ছবিঃ বক্সের ভেতরের ব্লিষ্টারের পেছনের অংশের ছবি<br>Photo of product: Secondary Package (side B)<br>Secondary package example: blister packs within a box; repackaging example	
1.16	ভিটামিন বা পুষ্টি সম্পূরক (১) এর ছবিঃ অন্যান্য বক্স, ব্লিষ্টার আর ওষুধের একসাথে ছবি<br>Photo of product: Other<br>Any other useful photos	

জেলা District	[ ][ ]
উপজেলা Upazila	[ ][ ]
স্থানের ধরণ Location	[ ][ ]
স্বাস্থ্যকেন্দ্রের ধরণ Facility Type	[ ][ ]
দোকানের ধরণ Retail Type	[ ][ ]
Freelist	[ ][ ]

MODULE 4: OTHER PRODUCTS (PILLS/TABLETS/SYRUP) FOR PREGNANT WOMEN			
**S.N.**	**QUESTION**		**RESPONSE CODE**
401 med	এই স্থান থেকে ভিটামিন বা পুষ্টি সম্পূরক এর মত অন্য কোন ধরনের ওষুধ (১) কি গর্ভবতী মহিলাদের বিতরণ করা হয় বা গর্ভবতী মহিলারা ক্রয় করে থাকেন? (গর্ভকালীন জটিলতার জন্য নয়।) Are there medicines or other products that have similar appearance to vitamins or nutrition supplements that are distributed to or purchase by pregnant women at this location? (Just for pregnancy, Not for any complication)	ধরন ১ Freelist item 1 (item1)	
		ধরন ২ Freelist item 2 (item2)	
		ধরন ৩ Freelist item 3 (item3)	
		ধরন ৪ Freelist item 4 (item4)	
		ধরন ৫ Freelist item 5 (item5)	
		ধরন ৬ Freelist item 6 (item6)	
		ধরন ৭ Freelist item 7 (item7)	
		ধরন ৮ Freelist item 8 (item8)	
		ধরন ৯ Freelist item 9 (item9)	
		ধরন ১০ Freelist item 10 (item10)	
		ধরন ১১ Freelist item 11 (item11)	
		ধরন ১২ Freelist item 12(item12)	
		ধরন ১৩ Freelist item 13(item13)	
		ধরন ১৪ Freelist item 14(item14)	
		ধরন ১৫ Freelist item 15(item15)	
		ধরন ১৬ Freelist item 16(item16)	

1.1	ওষুধ (১) এর ধরণ কি? What is the form of product?	আয়রন Iron = 01<br>ফলিক এসিড Folic acid = 02<br>আয়রন ফলিক এসিড Iron Folic Acid = 03<br>ক্যালসিয়াম Calcium (+/- Vit D) = 04<br>মাল্টিভিটামিন Multivitamin (3+ vitamins) = 05	

		কৃমিনাশক বড়ি<br>Deworming Tablets = 06<br>অন্যান্য (নির্দিষ্ট করুন) Other [SPECIFY] = 07	
1.2	ওষুধ (১) এ কোন কোন ভিটামিন থাকে?<br>What vitamins does it contain?		
1.3	ওষুধ (১) কিনতে এসে আপনার গ্রাহক/ক্রেতারা কি নাম/শব্দ ব্যবহার করেন?<br>What (informal) names/terms do your clients/customers use for this product?		
1.4	আপনি যখন আপনার গ্রাহক/ক্রেতার সাথে এই ওষুধ (১) নিয়ে কথা বলেন তখন আপনি কোন নাম/শব্দ ব্যবহার করেন? (যখন কেউ আপনার দোকানে আসে, তারা কীভাবে এই পণ্যটি চায়?)<br>What do name/terms do you use for this product when talking to your clients/customers? (Instruction for the interviewr: When someone comes to your shop, how do they ask for this product?)		
1.5	ওষুধ (১) গ্রাহক/ক্রেতার কাছে বিতরণের সময় কীভাবে প্যাকেজ করা হয়?<br>How is this packaged when distributed to client/customer?	মূল প্যাকেজিংঃ বক্স Original packaging: box = 01<br>মূল প্যাকেজিংঃ পূর্ণ ব্লিস্টার/ওষুধের পাতা Original packaging: Full blister pack = 02<br>মূল প্যাকেজিংঃ আংশিক ব্লিস্টার/ওষুধের পাতা Original packaging: Partial blister pack = 03<br>মূল প্যাকেজিংঃ বোতল Original packaging: bottle = 04<br>খোলা প্যাকেটঃ ছোট প্যাকেট/খাম Repackaged: sachet/envelope = 05<br><br>অন্যান্য (নির্দিষ্ট করুন) Other [SPECIFY] = 06	[ ][ ]

1.6	আর কিভাবে ওষুধ (১) প্যাকেজিং করা হয়?<br>How is this packaged? (Additional comments)		
1.7	এই ওষুধ (১) পেতে কি কোন স্বাস্থ্যকর্মীর প্রেসক্রিপশন প্রয়োজন হয়?<br>Does this product require a prescription from a health care provider?	হ্যা, সবসময় প্রেসক্রিপশন প্রয়োজন<br>Yes always requires prescription = 01<br>মাঝেমাঝে/ এটা নির্ভর করে<br>Sometimes / it depends (explain) = 02<br>না, প্রেসক্রিপশনের প্রয়োজন নেই<br>No does not require a prescription = 03	[ ][ ]
1.7.1	যদি 2, ব্যাখ্যা করুন If Option 2, Explain		
1.8	সাধারণত গ্রাহক/ক্রেতারা যখন ওষুধ নিতে আসেন তখন তারা কি পরিমাণ চেয়ে থাকেন? What is the general quantity of this product a client/customer asks for? Probe: Number of the product Example: # tablets, # boxes with X tabelets each, etc)		
1.9	প্রতিবার ভিজিটের সময় সাধারণত গ্রাহক/ক্রেতাকে কি পরিমাণ ভিটামিন বা পুষ্টি সম্পূরক (১) বিতরণ করা হয়?<br>What quantity of the product is usually provided to the client/customer during single visit?<br>যেমনঃ #ট্যাবলেট, #ট্যাবলেটের বক্স ইত্যাদি<br>Example: # tablets, # boxes with X tabelets each, etc)		[ ][ ]
1.10	কারা সাধারণত এই ভিটামিন বা পুষ্টি সম্পূরক (১) গ্রহণ/কিনে থাকেন?<br>(নির্দিষ্ট জনগোষ্ঠীর বিবরণঃ যারা প্রাইভেট হাসপাতাল/চেম্বার থেকে সেবা নেয়; উচ্চ আয় ইত্যাদি)<br>Who tends to receive/purchase this product?<br>(describe subgroup - e.g. private care, higher income, etc)		

1.11	ওষুধ (১) সম্পর্কে অন্যান্য কোন মন্তব্য<br>Other enumerator comments about product		
1.12	ওষুধ (১) এর ছবিঃ বক্সের সামনের অংশের ছবি<br>Photo of product: Primary package (side A)<br>Primary package example: box that contains blisters, original bottle,		
1.13	ওষুধ (১) এর ছবিঃ বক্সের পেছনের অংশের ছবি<br>Photo of product: Primary package (side B)<br>Primary package example: box that contains blisters, original bottle,		
1.14	ওষুধ (১) এর ছবিঃ বক্সের ভেতরের ব্লিষ্টারের সামনের অংশের ছবি<br>Photo of product: Secondary Package (side A)<br>Secondary package example: blister packs within a box; repackaging example		
1.15	ওষুধ (১) এর ছবিঃ বক্সের ভেতরের ব্লিষ্টারের পেছনের অংশের ছবি<br>Photo of product: Secondary Package (side B)<br>Secondary package example: blister packs within a box; repackaging example		
1.16	ওষুধ (১) এর ছবিঃ অন্যান্য<br>বক্স, ব্লিষ্টার আর ওষুধের একসাথে ছবি<br>Photo of product: Other<br>Any other useful photos		

Text S3: Landscaping Guide Ethiopia

1B. Freelisting/KII with Pharmacists, Pharmacy Staff, Health Workers, Market Sellers - Phase I

Name of venue:
Type of venue (check box):

- A. Pharmacy
- B. Private Heath Facility
- C. Market
    - 1. Supermarket
    - 2. Retail shop
    - 3. Kiosk
    - 4. Stalls in a non-permanent open-air market
    - 5. Stalls in a permanent market
    - 6. Government store
    - 99. Other, Specify

Sub-city:
Woreda:
Participant Name:
Participant ID:
Role of participant (e.g. pharmacist, pharmacy staff, health worker, market sellers):
Length of time working in this role (years):
Date of data collection:
Time Start of Interview:
Time End of Interview:
Code of the Enumerator

Step 1: Free Listing

Please list the names of products that women access here [list name of venue] while they are pregnant. [Interviewer should encourage participant to list as many products as possible, continue to probe for, "anything else" or "anymore."] [Write down everything the participant says – at this stage do not clarify] [Once participant is completely done their freelist and you have probed for "anything else" you can ask clarifying questions such as: you listed both nutrition supplement and vitamin, can you tell me more about each of these products]

Freelist

	Amharic (record exactly what they say)	English (to be filled out later)
1.		
2.		
3.		
4.		
5.		
6.		
7.		
8.		
9.		
10.		
11.		
12.		
13.		
14.		

Step 2: Product Specific Questions

For each product listed:

	Freelist item:	Freelist item:	Freelist item:
**Question / Probe**	**Notes**	**Notes**	**Notes**
1. Let's start with [list name of first product in table], can you show this product to me? Can you describe this product?			
2. Are there any informal names that your customers use for this product?			
3. What is the form of packaging when you initially receive the product here at facility/store?			
Probe: large bottle for repackaging; small bottles, large or small boxes with blister packs			
4. What does packaging look like when you sell or distribute the product to the woman?			

Probe: Do you repackage it?			
Probe: Is it always repackaged in the same way?			
Probe: Any other ways…?			
5. How often should a woman use this product?			
Probe based on response (for example if they say "all of the time", ask what all of the time means, if they say "only when they feel sick" ask them to talk more about what they mean when they say feel sick)			
6. Do you provide women with any instructions regarding this product?			
Probe: Frequency to consume?			
Probe: Purpose of the product?			

7. Do only pregnant women take this product?			
Probe: who else? For what?			
Probe: Do women take this product after the baby has come? What about before they are pregnant?			
**Observation**			
8. Description of the Packaging [*record size, color, shape, be as detailed as possible*]			
9. Frequency of Product Intake [*record any written instructions on frequency of intake*]			
10. Additional Observation Notes			
11. Did you obtain a physical sample?			

12. Did you take a picture?			

	Freelist item:	Freelist item:	Freelist item:
Question / Probe	Notes	Notes	Notes
1. Let's start with [list name of first product in table], can you show this product to me? Can you describe this product?			
2. Are there any informal names that your customers use for this product?			
3. What is the form of packaging when you initially receive the product here at facility/store?			
Probe: large bottle for repackaging; small bottles, large or small boxes with blister packs			
4. What does packaging look like when you sell or distribute the product to the woman?			
Probe: Do you repackage it?			
Probe: Is it always repackaged in the same way?			

Probe: Any other ways…?			
5. How often should a woman use this product?			
Probe based on response (for example if they say "all of the time", ask what all of the time means, if they say "only when they feel sick" ask them to talk more about what they mean when they say feel sick)			
6. Do you provide women with any instructions regarding this product?			
Probe: Frequency to consume?			
Probe: Purpose of the product?			
7. Do only pregnant women take this product?			
Probe: who else? For what?			

Probe: Do women take this product after the baby has come? What about before they are pregnant?			
**Observation**			
8. Description of the Packaging [*record size, color, shape, be as detailed as possible*]			
9. Frequency of Product Intake [*record any written instructions on frequency of intake*]			
10. Additional Observation Notes			
11. Did you obtain a physical sample?			
12. Did you take a picture?			

	**Freelist item:**	**Freelist item:**	**Freelist item:**
**Question / Probe**	**Notes**	**Notes**	**Notes**

1. Let's start with [list name of first product in table], can you show this product to me? Can you describe this product?			
2. Are there any informal names that your customers use for this product?			
3. What is the form of packaging when you initially receive the product here at facility/store?			
Probe: large bottle for repackaging; small bottles, large or small boxes with blister packs			
4. What does packaging look like when you sell or distribute the product to the woman?			
Probe: Do you repackage it?			
Probe: Is it always repackaged in the same way?			
Probe: Any other ways…?			

5. How often should a woman use this product?			
Probe based on response (for example if they say "all of the time", ask what all of the time means, if they say "only when they feel sick" ask them to talk more about what they mean when they say feel sick)			
6. Do you provide women with any instructions regarding this product?			
Probe: Frequency to consume?			
Probe: Purpose of the product?			
7. Do only pregnant women take this product?			
Probe: who else? For what?			
Probe: Do women take this product after the baby has come? What about before they are pregnant?			

Observation			
8. Description of the Packaging [*record size, color, shape, be as detailed as possible*]			
9. Frequency of Product Intake [*record any written instructions on frequency of intake*]			
10. Additional Observation Notes			
11. Did you obtain a physical sample?			
12. Did you take a picture?			

Data DENT
Data for Decisions in Nutrition

Text S4: Freelisting Semi-Structured Interview, Ethiopia

1A. Freelisting & KII Guide with Currently Pregnant or Recently Delivered Women-Phase I

Sub-city:
Woreda:
Participant Name:

- ☐ A. Currently Pregnant Women
- ☐ B. Recently Delivered Women

Participant ID:
GPS Coordinates:
Date of data collection:
Time Start of Interview:
Time End of Interview:
Code of Enumerator:

CURRENTLY PREGNANT RESPONDENT'S BACKGROUND AND PREGNANCY HISTORY

1.	How old are you? Can you tell me what month and year were you born?	Month [ ][ ]<br>98. Don't know month<br>Year [ ][ ][ ][ ]<br>99. Don't know year
2.	How old were you at your last birthday? (response required)	Age in completed years [ ][ ]
3.	What is the highest level of education you attended: primary, secondary, or higher?	1. None<br>2. Primary (grade 1 – 8)<br>3. Secondary (Grade 9 – 12)<br>4. Higher (college and above)
4.	Now I would like to ask about all the births you have had during your life. Have you ever given birth? **[If no, SKIP to Q7.]**	1. Yes<br>2. No
5.	How many total births have you given? (ask for the total number of births even if did not result in a live birth)?	Number of total births [ ][ ]
6.	How old is your youngest child?	1. Weeks [ ][ ]<br>2. Months [ ][ ]<br>3. Year [ ][ ]
7.	How many weeks or months pregnant are you?	1. Weeks [ ][ ]

		2. Months [ ][ ]
8.	Did you receive antenatal care during this pregnancy? **[If no, END INTERVIEW]**	1. No<br>2. Yes<br>98. Don't Know
9.	Where did you receive antenatal care for this pregnancy? Anywhere else?<br><br>RECORD ALL SOURCES. PROBE TO IDENTIFY TYPE OF SOURCE. IF UNABLE TO DETERMINE IF PUBLIC, PRIVATE, OR NGO SECTOR, RECORD 'X' AND WRITE THE NAME OF THE PLACE(S).	1. Home/ Her home<br>2. Other home<br>3. Government hospital<br>4. Government health center<br>5. Other public sector (specify)<br>6. Private hospital<br>7. Private clinic<br>8. Other private medical sector (specify)<br>9. NGO hospital<br>10. NGO clinic<br>11. Other NGO medical sector (specify)<br>12. Other, (specify)
10.	Whom did you see?<br><br>PROBE TO IDENTIFY EACH TYPE OF PERSON AND RECORD ALL MENTIONED.	1. Doctor<br>2. Nurse/midwife<br>3. Health officer<br>4. Traditional birth attendant<br>5. Health Extension Workers<br>6. Not able to differentiate<br>7. Other, (specify)
11.	How many weeks or months pregnant were you when you first received antenatal care for this pregnancy?	1. Weeks [ ][ ]<br>2. Months [ ][ ]<br>98. Don't know
12.	How many times have you received antenatal care during this pregnancy?	Number of times [ ][ ]<br>98. Don't know

RECENTLY DELIVERED RESPONDENT'S BACKGROUND AND PREGNANCY HISTORY

1.	How old are you? Can you tell me what month and year were you born?	Month [ ][ ]<br>98. Don't know month<br>Year [ ][ ][ ][ ]<br>99. Don't know month
2.	How old were you at your last birthday? (response required)	Age in completed years [ ][ ]
3.	What is the highest level of education you attended: primary, secondary, or higher?	1. None<br>2. Primary (grade 1 – 8)<br>3. Secondary (Grade 9 – 12)<br>4. Higher (college and above)
4.	Now I would like to ask about all the births you have had during your life. Have you ever given birth?<br>**[If no, END INTERVIEW]**	1. Yes<br>2. No
5.	How many total births have you given? (ask for the total number of births even if did not result in a live birth)?	Number of total births [ ][ ]
6.	How old is your youngest child?	1. Weeks [ ][ ]<br>2. Months [ ][ ]<br>3. Year [ ][ ]
7.	Did you see anyone for antenatal care during your most recent pregnancy? **[If no, END INTERVIEW**	1. No<br>2. Yes<br>98. Don't Know
8.	Where did you receive antenatal care during your most recent pregnancy? Anywhere else?<br><br>RECORD ALL SOURCES. PROBE TO IDENTIFY TYPE OF SOURCE. IF UNABLE TO DETERMINE IF PUBLIC, PRIVATE, OR NGO SECTOR, RECORD 'X' AND WRITE THE NAME OF THE PLACE(S).	1. Home/ Her home<br>2. Other home<br>3. Government hospital<br>4. Government health center<br>5. Other public sector (specify)<br>6. Private hospital<br>7. Private clinic<br>8. Other private medical sector (specify)<br>9. NGO hospital<br>10. NGO clinic<br>11. Other NGO medical sector (specify)<br>12. Other, (specify)
9.	Whom did you see? Anyone else?	1. Doctor<br>2. Nurse/midwife

	PROBE TO IDENTIFY EACH TYPE OF PERSON AND RECORD ALL MENTIONED.	3. Health officer<br>4. Traditional birth attendant<br>5. Health Extension Workers<br>6. Not able to differentiate<br>7. Other (specify
11.	How many weeks or months pregnant were you when you first received antenatal care for your most recent pregnancy?	1. Weeks [ ][ ]<br>2. Months [ ][ ]<br>98. Don't know
12.	How many times did you received antenatal care during your most recent pregnancy?	Number of times [ ][ ]<br>98. Don't know

Step 1: Freelisting
Interviewer: *When a women in your community is pregnant, what products are they exposed to at a health center, pharmacy, or market?* [Interviewer should encourage women to list as many products as possible, continue to probe for, "anything else" or "anymore."] [Write down everything the woman says – at this stage do not clarify]. [Once the woman is completely done with their freelist and you have probed for "anything else" you can ask clarifying questions such as: you listed both nutrition supplement and vitamin, can you tell me more about each of these products]
Freelist

	Amharic (record exactly what they say)	English (to be filled out later)
1.		
2.		
3.		
4.		
5.		
6.		
7.		
8.		
9.		
10.		
11.		
12.		

13.		
14.		

Step 2: KII Question Guide
Once the freelist activity has ended, begin KII.

	Freelist Item:	Freelist Item:	Freelist Item:
Question / Probe	Notes	Notes	Notes
1. Can you describe what this product is?			
2. Are there other names or words that people use for this product?			
Probe: formal			
Probe: informal			
3. Please describe what this product looks like?			
Probe: Color			

Probe: Shape			
Probe: Size			
4. Please describe the packaging this product comes in?			
Probe: Color			
Probe: Shape			
Probe: Text/words			
Probe: logos			
5. Where do pregnant women in your community most commonly access this product?			

6. How often should a woman take [product name] while she is pregnant? (for example if they say “all of the time”, ask what all of the time means, if they say “only when they feel sick” ask them to talk more about what they mean when they say feel sick)			
Probe: based on response			
7. Why should a pregnant woman take this product?			
Probe: Why do people take it? What is the purpose?			
8. Do women share this product with anyone else?			
Probe: With whom, how often?			

9. Is the product only for pregnant women?			
Probe: if no, what other types of people use this product? (sick people, children, men, elderly, etc.)			
10. Does a woman take this product before she is pregnant?			
Probe: When does she start taking it			
11. Does a women take this product after baby is delivered?			
Probe: When stop taking?			
12. If a woman said she took after baby came or before pregnant: How often should a woman take [product name] [after the baby comes] [before they are pregnant]?			

Probe based on response (for example if they say “all of the time”, ask what all of the time means, if they say “only when they feel sick” ask them to talk more about what they mean when they say feel sick)			

	**Freelist Item:**	**Freelist Item:**	**Freelist Item:**
**Question / Probe**	**Notes**	**Notes**	**Notes**
1. Can you describe what this product is?			
2. Are there other names or words that people use for this product?			
Probe: formal			
Probe: informal			

3. Please describe what this product looks like?			
Probe: Color			
Probe: Shape			
Probe: Size			
4. Please describe the packaging this product comes in?			
Probe: Color			
Probe: Shape			
Probe: Text/words			
Probe: logos			

5. Where do pregnant women in your community most commonly access this product?			
6. How often should a woman take [product name] while she is pregnant? (for example if they say "all of the time", ask what all of time means, if they say "only when they feel sick" ask them to talk more about what they mean when they say feel sick)			
Probe: based on response			
7. Why should a pregnant woman take this product?			
Probe: Why do people take it? What is the purpose?			
8. Do women share this product with anyone else?			

Probe: With whom, how often?			
9. Is the product only for pregnant women?			
Probe: if no, what other types of people use this product? (sick people, children, men, elderly, etc.)			
10. Does a woman take this product before she is pregnant?			
Probe: When does she start taking it			
11. Does a women take this product after baby is delivered?			
Probe: When stop taking?			

12. If a woman said she took after baby came or before pregnant: How often should a woman take [product name] [after the baby comes] [before they are pregnant]?			
Probe based on response (for example if they say "all of the time", ask what all of the time means, if they say "only when they feel sick" ask them to talk more about what they mean when they say feel sick)			

Step 2: KII Question Guide
Once the freelist activity has ended, begin KII.

	Freelist Item:	Freelist Item:	Freelist Item:
**Question / Probe**	**Notes**	**Notes**	**Notes**
1. Can you describe what this product is?			
2. Are there other names or words that people use for this product?			
Probe: formal			
Probe: informal			
3. Please describe what this product looks like?			
Probe: Color			

Probe: Shape			
Probe: Size			
4. Please describe the packaging this product comes in?			
Probe: Color			
Probe: Shape			
Probe: Text/words			
Probe: logos			
5. Where do pregnant women in your community most commonly access this product?			

6. How often should a woman take [product name] while she is pregnant? (for example if they say "all of the time", ask what all of the time means, if they say "only when they feel sick" ask them to talk more about what they mean when they say feel sick)			
Probe: based on response			
7. Why should a pregnant woman take this product?			
Probe: Why do people take it? What is the purpose?			
8. Do women share this product with anyone else?			
Probe: With whom, how often?			

9. Is the product only for pregnant women?			
Probe: if no, what other types of people use this product? (sick people, children, men, elderly, etc.)			
10. Does a woman take this product before she is pregnant?			
Probe: When does she start taking it			
11. Does a women take this product after baby is delivered?			
Probe: When stop taking?			
12. If a woman said she took after baby came or before pregnant: How often should a woman take [product name] [after the baby comes] [before they are pregnant]?			

Probe based on response (for example if they say “all of the time”, ask what all of the time means, if they say “only when they feel sick” ask them to talk more about what they mean when they say feel sick)			

Ask this question at the very end of the KII (not after each product).
13. Is there anything else that you would like to mention or discuss on your freelist?

Data DENT
Data for Decisions in Nutrition

Addis Continental Institute of Public Health

Text S5: Pile-Sorting Semi-Structured Interview, Ethiopia

1C. Pile-Sorting Guide with Currently Pregnant and Recently Delivered Women

Health Facility Name Where Interview Conducted:
Sub-city:
Woreda:
Participant Name:

- ☐ A. Currently Pregnant Women
- ☐ B. Recently Delivered Women

Participant ID:
GPS Coordinates:
Date of data collection:
Time Start of Interview:
Time End of Interview:
Code of Enumerator:
Step 1: Respondent Background

CURRENTLY PREGNANT RESPONDENT'S BACKGROUND AND PREGNANCY HISTORY

1.	How old are you? Can you tell me what month and year were you born?	Month [ ][ ]<br>98. Don't know month<br>Year [ ][ ][ ][ ]<br>99. Don't know year
2.	How old were you at your last birthday? (response required)	Age in completed years [ ][ ]
3.	What is the highest level of education you attended: primary, secondary, or higher?	5. None<br>6. Primary (grade 1 – 8)<br>7. Secondary (Grade 9 – 12)<br>8. Higher (college and above)
4.	Now I would like to ask about all the births you have had during your life. Have you ever given birth? **[If no, SKIP to Q7.]**	3. Yes<br>4. No
5.	How many total births have you had? (ask for the total number of births even if did not result in a live birth)?	Number of total births [ ][ ]
6.	How old is your youngest child?	4. Weeks [ ][ ]<br>5. Months [ ][ ]<br>6. Year [ ][ ]
7.	How many weeks or months pregnant are you?	3. Weeks [ ][ ]

		4. Months [ ][ ]
8.	Did you receive antenatal care during this pregnancy? **[If no, END INTERVIEW]**	3. No<br>4. Yes<br>98. Don't Know
9.	Where did you receive antenatal care for this pregnancy? Anywhere else?<br><br>RECORD ALL SOURCES. PROBE TO IDENTIFY TYPE OF SOURCE. IF UNABLE TO DETERMINE IF PUBLIC, PRIVATE, OR NGO SECTOR, RECORD 'X' AND WRITE THE NAME OF THE PLACE(S).	13. Home/ Her home<br>14. Other home<br>15. Government hospital<br>16. Government health center<br>17. Other public sector (specify)<br>18. Private hospital<br>19. Private clinic<br>20. Other private medical sector (specify)<br>21. NGO hospital<br>22. NGO clinic<br>23. Other NGO medical sector (specify)<br>24. Other, (specify)
10.	Whom did you see?<br><br>PROBE TO IDENTIFY EACH TYPE OF PERSON AND RECORD ALL MENTIONED.	8. Doctor<br>9. Nurse/midwife<br>10. Health officer<br>11. Traditional birth attendant<br>12. Health Extension Workers<br>13. Not able to differentiate<br>14. Other, (specify)
11.	How many weeks or months pregnant were you when you first received antenatal care for this pregnancy?	3. Weeks [ ][ ]<br>4. Months [ ][ ]<br>98. Don't know
12.	How many times have you received antenatal care during this pregnancy?	Number of times [ ][ ]<br>98. Don't know

Data DENT
Data for Decisions in Nutrition

Addis Continental Institute of Public Health

RECENTLY DELIVERED RESPONDENT'S BACKGROUND AND PREGNANCY HISTORY

1.	How old are you? Can you tell me what month and year were you born?	Month [ ][ ]<br>98. Don't know month<br>Year [ ][ ][ ][ ]<br>99. Don't know month
2.	How old were you at your last birthday? (response required)	Age in completed years [ ][ ]
3.	What is the highest level of education you attended: primary, secondary, or higher?	5. None<br>6. Primary (grade 1 – 8)<br>7. Secondary (Grade 9 – 12)<br>8. Higher (college and above)
4.	Now I would like to ask about all the births you have had during your life. Have you ever given birth?<br>**[If no, END INTERVIEW]**	3. Yes<br>4. No
5.	How many total births have you given? (ask for the total number of births even if did not result in a live birth)?	Number of total births [ ][ ]
6.	How old is your youngest child?	4. Weeks [ ][ ]<br>5. Months [ ][ ]<br>6. Year [ ][ ]
7.	Did you see anyone for antenatal care during your most recent pregnancy? **[If no, END INTERVIEW]**	3. No<br>4. Yes<br>98. Don't Know
8.	Where did you receive antenatal care during your most recent pregnancy? Anywhere else?<br><br>RECORD ALL SOURCES. PROBE TO IDENTIFY TYPE OF SOURCE. IF UNABLE TO DETERMINE IF PUBLIC, PRIVATE, OR NGO SECTOR, RECORD 'X' AND WRITE THE NAME OF THE PLACE(S).	13. Home/ Her home<br>14. Other home<br>15. Government hospital<br>16. Government health center<br>17. Other public sector (specify)<br>18. Private hospital<br>19. Private clinic<br>20. Other private medical sector (specify)<br>21. NGO hospital<br>22. NGO clinic<br>23. Other NGO medical sector (specify)<br>24. Other, (specify)

9.	Whom did you see? Anyone else?<br><br>PROBE TO IDENTIFY EACH TYPE OF PERSON AND RECORD ALL MENTIONED.	8. Doctor<br>9. Nurse/midwife<br>10. Health officer<br>11. Traditional birth attendant<br>12. Health Extension Workers<br>13. Not able to differentiate<br>14. Other (specify)
10..	How many weeks or months pregnant were you when you first received antenatal care for your most recent pregnancy?	3. Weeks [ ][ ]<br>4. Months [ ][ ]<br>98. Don't know
11.	How many times did you received antenatal care during your most recent pregnancy?	Number of times [ ][ ]<br>98. Don't know

Step 2A: Facilitated Multi-Sort Pile Sorting:
Interviewer: ***Here are a set of cards representing different products that women may be exposed to during pregnancy. I'd like you to start by identifying all the products that you recognize and telling me what you call them.***
Probes: Can you tell me what this product is for? How did you identify this product? (eg: by its packaging box, bottle, strips, pills) [make sure to confirm the products that they recognize and probe to ensure nothing is missing].
List the Names of Cards Selected and indicate image number (all cards will be labeled with letters A, B, C, D, etc.):

	Card name and image number (include * next to the name and image number for products that were introduced by the interviewer)	Interviewer: What do you call this product?	Interviewer: What is this product for?	Interviewer: How did you identify this product? (eg: by its packaging box, bottle, strips, pills) Probe: **Did you read the package to help identify the product?**
1				
2				
3				
4				

5				
6				
7				
8				
9				
10				

Note that if in the first pass through the cards the participant did not identify MMS or IFA, then before moving to the next step (2B) enumerator should identify the cards with MMS and IFA and explain what each product is. MMS and IFA should then be included in the step 2B pilesort exercise

- MMS (Unimmap)-This is a pill that has many vitamins and minerals that are good for women to take when they are pregnant
- IFA (Iron Folate – Ferrous Sulfate 200 mg + Folic Acid 0.4 mg Tablets)-This is a pill that has iron and folic acid that are good for women to take when they are pregnant

Step 2B: Initial Pilesort

Interviewer: ***Now that we have a set of cards with products that you recognize, I'd like for you to sort the cards into piles according to how similar the products on the card are. You can make as many or as few piles as you'd like. There are multiple copies of each card because you can put a product into more than one pile.***

NOTE: [if there are two or less cards identified, skip to step 2C]

Step 2B: Once the participant has created their pile(s), have them discuss each pile:	
1. Tell me about this pile. Do you have a name that you would give this pile?<br><br>(Clearly identify each pile in your field notes. Take a picture of each pile and indicate which cards are in each pile]	Pile 1
	Pile 2
	Pile 3
2. How did you decide to group these cards in this pile?	Pile 1
	Pile 2
	Pile 3
3. What is similar about all of the cards in this pile?	Pile 1

	Pile 2
	Pile 3
4. How would you compare the piles to one another? What is distinct or different about each pile?	
5. Did you have any difficulty deciding where to put any of the cards?	
Step 2C: Can you please pick up the cards and sort them again into two piles: one pile of cards that have vitamins (micronutrients) (e.g., iron, vitamin, prenatal) that women might take during pregnancy and a pile that contains products that are not vitamins for women during pregnancy?	
6. Can you tell me about the cards in your piles (e.g., similarities, differences, product purposes)?	Pile 1
	Pile 2
7. Did you have any difficulty deciding where to put any of the cards?	
Step 2D: Now can you resort the cards by making a pile with cards that have products that women should take daily for their entire pregnancy?	
8. Can you tell me about the cards in your pile (e.g., similarities, differences, product purposes)?	Pile 1

	Pile 2
9. Did you have any difficulty deciding where to put any of the cards?	
**Step 3: Additional Questions**	
10. Show me which cards have a product that contains iron	
11. Show me which cards have a product that contains a vitamin(s) (multivitamin)?	
12. Show me which cards have a product that would be considered a prenatal?	
13. How is this card [hold up IFA card] and [uinimap card] different? How are they similar?	
For Pregnant Women Ask:	
14. During this pregnancy, were you given or did you buy any iron-containing tablets or iron-containing syrup? *Repeat questions*	

14-17 for all iron-containing products (e.g. iron folate, ferrous sulfate, foliron, prenatal, Promam, Fenza)<br><br>Can you point to which product(s) you were given or bought?		
15. How many of these [NAME OF PRODUCT(S) LISTED IN Q14] have you consumed during your entire pregnancy so far?<br>a. How did you estimate how many you consumed during your pregnancy so far?		
Additional possible probes:	Did you think about when you first received the product?	
	Did you think about how frequently you received the product?	
	Did you think about where you received the product?	

	Did you think about your routine when taking the product?	
16. What about in the last month?<br>a. How did you estimate how many you consumed last month?		
17. What about in the last week?<br>a. How did you estimate how many you consumed last week?		
For Recently Delivered Women Ask:		
18. During your last pregnancy, were you given or did you buy any iron-containing tablets or iron-containing syrup? *Repeat questions 18-19 for all iron-containing products (e.g. iron folate, ferrous sulfate, foliron, prenatal, Promam, Fenza)*<br>a. Can you point to which product(s) you were given or bought?		

19. How many [NAME OF PRODUCT(S) LISTED IN Q18] did you consume during your entire pregnancy?<br>a. How did you estimate how many you consumed during your pregnancy so far?		
Additional possible probes:	Did you think about when you first received the product?	
	Did you think about how frequently you received the product?	
	Did you think about where you received the product?	
	Did you think about your routine when taking the product?	

Data DENT
ta for Decisions in Nutrition

Addis Continental Institute of Public Health

Text S6: Round 1 Cognitive Interview, CPW, Ethiopia

PHASE II: COGNITIVE INTERVIEW WITH CURRENTLY PREGNANT WOMEN

MODULE 1. IDENTIFICATION

IDENTIFICATION	RESPONSE/CODE
NAME OF THE HEALTH FACILITY [Only to fill in if conducted at health facility]	
NAME OF THE SUB-CITY	
NAME OF THE WOREDA	
MOTHER ID#	
PRIMARY LANGUAGE SPOKEN	
MOBILE NUMBER WHERE RESPONDENT CAN BE REACHED	
**INTERVIEWER INFORMATION**	**CODE**
CODE OF THE INTERVIEWER	
CODE OF THE NOTE-TAKER/SECOND INTERVIEWER	
DATE OF THE INTERVIEW	[ ][ ] : [ ][ ] DAY MONTH
START TIME (24-HOUR CLOCK)	[ ][ ] : [ ][ ] HR MIN
END TIME (24-HOUR CLOCK)	[ ][ ] : [ ][ ] HR MIN
DURATION (HOUR/MIN)	[ ][ ] : [ ][ ] HR MIN
**RESULT CODES:**	
COMPLETE....................1<br>PARTIALLY COMPLETE....................2	REFUSAL....................3<br>ELIGIBLE RESPONDENT AWAY FOR EXTENDED PERIOD....................4

MODULE 2. CURRENTLY PREGNANT RESPONDENT'S BACKGROUND AND PREGNANCY HISTORY

1.	How old are you? Can you tell me what month and year were you born?	Month [ ][ ]<br>98. Don't know month<br>Year [ ][ ][ ][ ]<br>99. Don't know year
2.	How old were you at your last birthday? (response required)	Age in completed years [ ][ ]
3.	What is the highest level of education you attended: primary, secondary, or higher?	9. None<br>10. Primary (grade 1 – 8)<br>11. Secondary (Grade 9 – 12)<br>12. Higher (college and above)
4.	Have you lived in Addis Ababa continuously for the last year?	5. Yes → 5<br>6. No
4a.	If no, when did you come to Addis Ababa?	[ ][ ] [ ][ ]][ ]<br>MONTH YEAR<br>98. Don't know
5.	Now I would like to ask about all the births you have had during your life. Have you ever given birth? **[If no, SKIP to Q7.]**	1. Yes<br>2. No
6.	How many total births have you had? (ask for the total number of births even if did not result in a live birth)?	Number of total births [ ][ ]
7.	How old is your youngest child?	7. Weeks [ ][ ]<br>8. Months [ ][ ]<br>9. Year [ ][ ]
8.	How many weeks or months pregnant are you?	5. Weeks [ ][ ]<br>6. Months [ ][ ]
9.	Did you receive antenatal care during this pregnancy? **[If no, END INTERVIEW]**	5. No<br>6. Yes<br>98. Don't Know
10	Where did you receive antenatal care for this pregnancy? Anywhere else?<br><br>RECORD ALL SOURCES. PROBE TO IDENTIFY TYPE OF SOURCE. IF UNABLE	25. Home/ Her home<br>26. Other home<br>27. Government hospital<br>28. Government health center<br>29. Other public sector (specify)

	TO DETERMINE IF PUBLIC, PRIVATE, OR NGO SECTOR, RECORD 'X' AND WRITE THE NAME OF THE PLACE(S).	30. Private hospital<br>31. Private clinic<br>32. Other private medical sector (specify)<br>33. NGO hospital<br>34. NGO clinic<br>35. Other NGO medical sector (specify)<br>36. Other, (specify)
11.	Whom did you see?<br><br>PROBE TO IDENTIFY EACH TYPE OF PERSON AND RECORD ALL MENTIONED.	15. Doctor<br>16. Nurse/midwife<br>17. Health officer<br>18. Traditional birth attendant<br>19. Health Extension Workers<br>20. Not able to differentiate<br>21. Other, (specify)
12.	How many weeks or months pregnant were you when you first received antenatal care for this pregnancy?	5. Weeks [ ][ ]<br>6. Months [ ][ ]<br>98. Don't know
13.	How many times have you received antenatal care during this pregnancy?	Number of times [ ][ ]<br>98. Don't know

Data DENT
Data for Decisions in Nutrition

Addis Continental Institute of Public Health

MODULE 3. MICRONUTRIENT SUPPLEMENTATION QUESTIONS

INTERVIEWER READ ALOUD: Today, I will be asking you questions about products for pregnant women. We would like to know if you understand the questions and how you understand them. The questions we are about to ask you might seem simple or repetitive. But your responses are important to us. There are also no right or wrong answers.

**1.**	**During this pregnancy have you been given or have you bought any vitamin tablet or syrup?**<br>DO NOT SHOW VISUAL AID	YES . . . . …… . 1<br>NO . . . . 2<br>DON'T KNOW . . . . …. 8
	Q1. OBSERVATIONS<br>• Note pause, facial expression, verbal responses, etc	
**1a.**	Was it easy or difficult to give an answer to this question?<br>Why was it [EASY / DIFFICULT]?	
**1b.**	I asked **"During this pregnancy have you been given or have you bought any vitamin tablet or syrup?"** In your own words can you tell me what I have just asked you?	
**1c.**	Can you explain to me the time period the question was talking about?	
**1d.**	I asked about "**vitamin**" in my question. Can you explain to me what **"vitamin"** means in your own words?	
**1e.**	Could you tell me some examples of vitamins that women take during pregnancy?	
**1f.**	I asked **"have you given or have you bought".** Can you explain to me what **"have you given or have you bought"** means in your own words?	
**1g.**	Q1 INTERVIEWER NOTES	

			YES	NO	DON'T KNOW
2	**During this pregnancy have you been given or have you bought any of these vitamin tablets or syrups:**<br><br>A. IRON OR IRON AND FOLIC ACID?<br>B. PRENATAL?<br>C. FOLIC ACID?<br>D. ANY OTHER VITAMIN? (SPECIFY)<br><br>DO NOT SHOW VISUAL AID	a. Iron<br>b. Prenatal<br>c. Folic Acid<br>d. Other vitamin	1<br>1<br>1<br>1	2<br>2<br>2<br>2	8<br>8<br>8<br>8
		SPECIFY Other vitamin			

	Q2. OBSERVATIONS<br>Note pause, facial expression, verbal responses, etc	
**2a.**	Where these questions easy or difficult for you to answer?<br><br>Why was it [EASY / DIFFICULT]?	
**2b.**	I asked "**During this pregnancy have you been given or have you bought any of these vitamin tablets or syrups**" In your own words can you tell me what I have just asked you?	
**2c.**	I asked about "iron or iron folic acid" in my question. Can you explain to me what "iron" means to you in your own words	
**2d.**	Can you tell me what " iron folic acid" means to you in your own words?	
**2e.**	I used the word "prenatal" in my question. Can you explain to me what "prenatal" mean in your own words?	
**2f.**	I used the word "folic acid" in my question. Can you explain to me what "folic acid" means in your own words?	
**2g.**	I used the words "any other vitamin" in my question. Can you explain to me what "any other vitamin" means in your own words?	
INTERVIEWER READ ALOUD: Now I am going to show you pictures and ask you the question again		
**2h.**	SHOW VISUAL AID IRON-3<br><br>**"During this pregnancy have you been given or have you bought any iron or iron-folic acid?"**	

	Do the pictures make it easier or more difficult to answer this question?<br>Why was it [EASIER/ MORE DIFFICULT]?	
2i.	SHOW VISUAL AID PRENATAL-3<br>**"During this pregnancy have you been given or have you bought any prenatal?"**<br>Do the pictures make it easier or more difficult to answer this question?<br>Why was it [EASIER/ MORE DIFFICULT]?	
2j	SHOW VISUAL AID - FOLIC ACID -3<br>**"During this pregnancy have you been given or have you bought any folic acid?"**<br>Do the pictures make it easier or more difficult to answer this question?<br>Why was it [EASIER/ MORE DIFFICULT]?	
2k	IF ANSWERS TO Q2 h-j ARE DIFFERENT FROM Q2 A-D<br>I noticed that you gave a different answer after I shared the photo, why did you change your answer?	NOT DIFFERENT………………………SKIP<br>DIFFERENT………………………(explain)
2l.	Q2 INTERVIEWER NOTES	

7.	**During this pregnancy where do you get the vitamin tablets or syrup? Anywhere else?**<br>PROBE TO IDENTIFY THE TYPE OF SOURCE. IF UNABLE TO DETERMINE IF PUBLIC, PRIVATE, OR NGO SECTOR, RECORD 'X' AND WRITE THE NAME OF THE PLACE(S).	**ADD ETHIOPIAN DHS HIGH LEVEL RESPONSE CATEGORIES**
	Q3 OBSERVATIONS<br>Note pause, facial expression, verbal responses, etc	
3a.	Was it easy or difficult to remember **where you got** the vitamin tablets or syrups?	

	Why was it [EASY/ DIFFICULT]?	
**3b.**	How do you remember where you get the vitamin tablets or syrups?	
**3c.**	IF Q2 RESPOND "YES" TO >1<br><br>Earlier you said that you got [IRON] Where do you get this?	Q2 yes to 1……………………………….SKIP<br>Q2 yes to >1 ………………….(explain)<br><br>Answer: From here.
**3d.**	IF Q2 RESPOND "YES" TO >1<br><br>Earlier you said that you got [PRENATAL] Where do you get this?	Q2 yes to 1……………………………….SKIP<br>Q2 yes to >1 ………………….(explain)
**3e.**	IF Q2 RESPOND "YES" TO >1<br><br>Earlier you said that you got [FOLIC ACID] Where do you get this?	Q2 yes to 1……………………………….SKIP<br>Q2 yes to >1 ………………….(explain)<br><br>Answer: From here.
**3f.**	IF Q2 RESPOND "YES" TO >1<br><br>Earlier you said that you got [OTHER VITAMIN] Where do you get this?	Q2 yes to 1……………………………….SKIP<br>Q2 yes to >1 ………………….(explain)

**4.**	**During this pregnancy, how many** weeks or months pregnant **were you when you first started taking a vitamin tablet or syrup?**	1. Weeks [ ][ ]<br>2. Months [ ][ ]<br>98. Don't know
	Q4 OBSERVATIONS<br><br>Note pause, facial expression, verbal responses, etc	
**4a.**	Was this question easy or difficult for you to answer?<br><br>Why was it [EASY/ DIFFICULT]?	
**4b.**	I used the words "**how many weeks or months pregnant**". Can you explain to me what **"how many weeks or months pregnant"** means in your own words?	

Data DENT
Data for Decisions in Nutrition

Addis Continental Institute of Public Health

**4c**	You said you first started taking vitamins when you were weeks or months pregnant. How did you come up with the answer of weeks or months?	
**4d.**	You said you first started taking when you were weeks or months pregnant, how sure are you of that?	
**4e.**	IF Q2 RESPOND “YES” TO >1<br><br>Earlier you said that you got [IRON] how many **weeks or** **<u>months pregnant</u>** were you when you **<u>first started</u>** taking [IRON]?	Q2 yes to 1……………………………….SKIP<br>Q2 yes to >1 ………………<br>1. Weeks [ ][ ]<br>2. Months [ ][ ]<br>98. Don't know
**4f.**	IF Q2 RESPOND “YES” TO >1<br><br>Earlier you said that you got [PRENATAL] how many **<u>weeks o</u>**r **<u>months pregnant</u>** were you when you **<u>first</u>** **<u>started</u>** taking [PRENTAL]?	Q2 yes to 1……………………………….SKIP<br>Q2 yes to >1 ………………<br>1. Weeks [ ][ ]<br>2. Months [ ][ ]<br>98. Don't know
**4g.**	IF Q2 RESPOND “YES” TO >1<br><br>Earlier you said that you got [[FOLIC ACID] how many **<u>weeks or</u>** **<u>months pregnant</u>** were you when you **<u>first</u>** **<u>started</u>** taking [FOLIC ACID]?	Q2 yes to 1……………………………….SKIP<br>Q2 yes to >1 ………………<br>1. Weeks [ ][ ]<br>2. Months [ ][ ]<br>98. Don't know
**4h.**	IF Q2 RESPOND “YES” TO >1<br><br>Earlier you said that you got [[OTHER VITAMINS] how many **weeks or** **<u>months pregnant</u>** were you when you **<u>first started</u>** taking [FOLIC ACID]?	Q2 yes to 1……………………………….SKIP<br>Q2 yes to >1 ………………<br>1. Weeks [ ][ ]<br>2. Months [ ][ ]<br>98. Don't know
**4i.**	Q4 INTERVIEWER NOTES	

Data DENT
Data for Decisions in Nutrition

Addis Continental Institute of Public Health

5.	**During the last <u>month</u>, for how many days did you take the a vitamin tablet or syrup?**<br><br>**IF ANSWER IS NOT NUMERIC, ASK FOR THE APPROXIMATE NUMBER OF DAYS THEY TOOK THE TABLET OR SYRUP.**	[ ][ ]NUMBER OF DAYS (0-31)<br>998 = Does not know
	Q6 OBSERVATIONS<br><br>Note pause, facial expression, verbal responses, etc	
5a.	Was it easy or difficult to remember how many days you took a vitamin tablet or syrup over the last month?<br><br>Why was it [EASY/ DIFFICULT]?	
5b.	I asked "**during the last month**" How did you count "**during the last month**"?<br><br>Probe: People think of a month in different ways. Did you think about the [LAST HOLIDAY/ LAST ANC VISIT/ LAST 30 DAYS]?	
5c.	I asked "**how many days did you take**" Can you explain to me what "**take**" means in your own words?<br><br>Probe: For you, does take mean that you consume the tablet or syrup or does it mean that you received the tablet?	
5d.	You said you took for days, How did you come up with the answer days?	
5e.	You said you took it for days, How sure are you of that?	
5f.	Q6 INTERVIEWER NOTES	

6.	**During the last month, how often did you take vitamin tablets or syrups? (Read responses)**	Daily ....1<br>A few days a week ....2<br>One day a week ....3<br>A few days a month ....4<br>One day per month or fewer…………5<br>Other (specify)…………………………………………6
6a.	Q7 OBSERVATIONS<br><br>Note pause, facial expression, verbal responses, etc	

**6b**	Was it easy or difficult to decide what answer to choose?<br><br>Why was it [EASY/ DIFFICULT]?	
**6c.**	I asked "**how often did you take**" in my question. Can you explain to me what "**how often did you take**" means to you in your own words?	
**6d.**	How did you come up with the answer of ?	
**6e.**	I used the word "**daily**". Can you explain to me what **"daily"** means to you in your own words?	
**6f.**	I said "**a few days per week**". Can you explain to me what **"a few days per week"** means to you in your own words?	
**6g.**	How many days is "**a few days per week**"?	
**6h.**	I said "**a few days a month**". Can you explain to me what "a few days a month" means to you in your own words?	
**6i.**	How many days is "**a few days a month**"?	
**6j.**	Q7 INTERVIEWER NOTES	

**7.**	**During the last 7 days, for how many days did you take the a vitamin tablet or syrup?**<br><br>**IF ANSWER IS NOT NUMERIC, PROBE FOR APPROXIMATE NUMBER OF DAYS.**	[ ] NUMBER OF DAYS (0-7)<br>998 = Does not know
	Q7 OBSERVATIONS<br><br>Note pause, facial expression, verbal responses, etc	
**7a.**	Was it easy or difficult is it to remember how many days you took a vitamin tablet or syrup over the last 7 days?<br><br>Why was it [EASY/ DIFFICULT]?	
**7b**	Can you explain to me the time period the question was talking about?	
**7c.**	You said you took for days, how did you come up with the answer of days?	

**7d.**	You said you took for days, How sure are you of that?	
**7e.**	Q7 INTERVIEWER NOTES	

Text S7: Round 1 Cognitive Interview RDW Ethiopia

PHASE II: COGNITIVE INTERVIEW WITH RECENTLY DELIVERED WOMEN

MODULE 1. IDENTIFICATION

IDENTIFICATION	RESPONSE/CODE
NAME OF THE HEALTH FACILITY [Only to fill in if conducted at health facility]	
NAME OF THE SUB-CITY	
NAME OF THE WOREDA	
NAME OF MOTHER	
MOTHER ID#	
PRIMARY LANGUAGE SPOKEN	
MOBILE NUMBER WHERE RESPONDENT CAN BE REACHED	
**INTERVIEWER INFORMATION**	**CODE**
CODE OF THE INTERVIEWER	
CODE OF THE NOTE-TAKER/SECOND INTERVIEWER	
DATE OF THE INTERVIEW	[ ][ ] [ ][ ] DAY MONTH
START TIME (24-HOUR CLOCK)	[ ][ ] : [ ][ ] HR MIN
END TIME (24-HOUR CLOCK)	[ ][ ] : [ ][ ] HR MIN
DURATION (HOUR/MIN)	
**RESULT CODES:**<br>COMPLETE.. 1<br>PARTIALLY COMPLETE 2	REFUSAL.. 3<br>ELIGIBLE RESPONDENT AWAY FOR EXTENDED PERIOD..................................... 4

MODULE 2. RECENTLY DELIVERED RESPONDENT'S BACKGROUND AND PREGNANCY HISTORY

1.	How old are you? Can you tell me what month and year were you born?	Month [ ][ ]<br>98. Don't know month<br>Year [ ][ ][ ][ ]<br>99. Don't know month
2.	How old were you at your last birthday? (response required)	Age in completed years [ ][ ]
3.	What is the highest level of education you attended: primary, secondary, or higher?	9. None<br>10. Primary (grade 1 – 8)<br>11. Secondary (Grade 9 – 12)<br>12. Higher (college and above)
4.	Have you lived in Addis Ababa continuously for the last year?	1. Yes → Skip to 5<br>2. No
4a.	If no, when did you come to Addis Ababa?	[ ][ ] [ ][ ]][ ]<br>MONTH YEAR<br>98. Don't know
5.	Now I would like to ask about all the births you have had during your life. Have you ever given birth?<br>**[If no, END INTERVIEW]**	5. Yes<br>6. No
6.	How many total births have you given? (ask for the total number of births even if did not result in a live birth)?	Number of total births [ ][ ]
7.	How old is your youngest child?	7. Weeks [ ][ ]<br>8. Months [ ][ ]<br>9. Year [ ][ ]
8.	Did you see anyone for antenatal care during your most recent pregnancy? **[If no, END INTERVIEW]**	5. No<br>6. Yes<br>98. Don't Know
9.	Where did you receive antenatal care during your most recent pregnancy? Anywhere else?<br><br>RECORD ALL SOURCES. PROBE TO IDENTIFY TYPE OF SOURCE. IF UNABLE TO DETERMINE IF PUBLIC, PRIVATE, OR NGO SECTOR, RECORD 'X' AND WRITE THE NAME OF THE PLACE(S).	25. Home/ Her home<br>26. Other home<br>27. Government hospital<br>28. Government health center<br>29. Other public sector (specify)<br>30. Private hospital<br>31. Private clinic<br>32. Other private medical sector (specify)<br>33. NGO hospital<br>34. NGO clinic<br>35. Other NGO medical sector (specify)

		36. Other, (specify)
	Whom did you see? Anyone else?<br><br>PROBE TO IDENTIFY EACH TYPE OF PERSON AND RECORD ALL MENTIONED.	15. Doctor<br>16. Nurse/midwife<br>17. Health officer<br>18. Traditional birth attendant<br>19. Health Extension Workers<br>20. Not able to differentiate<br>21. Other (specify)
10.	How many weeks or months pregnant were you when you first received antenatal care for your most recent pregnancy?	5. Weeks [ ][ ]<br>6. Months [ ][ ]<br>98. Don’t know
11.	How many times did you received antenatal care during your most recent pregnancy?	Number of times [ ][ ]<br>98. Don't know

MODULE 3. MICRONUTRIENT SUPPLEMENTATION QUESTIONS

INTERVIEWER READ ALOUD: Today, I will be asking you questions about products for pregnant women. We would like to know if you understand the questions and how you understand them. The questions we are about to ask you might seem simple or repetitive. But your responses are important to us. There are also no right or wrong answers.

**1.**	**During your last pregnancy were you given or did you buy any vitamin tablet or syrup?**<br>DO NOT SHOW VISUAL AID	YES . . . . 1<br>NO . . . . 2<br>DON'T KNOW 8
	Q1. OBSERVATIONS<br>Note pause, facial expression, verbal responses, etc	
**1a.**	•<br>Was it easy or difficult to give an answer to this question?<br>Why was it [EASY / DIFFICULT]?	
**1b.**	I asked **"During your last pregnancy were you given or did you buy any vitamin tablet or syrup?"** In your own words can you tell me what I have just asked you?	
**1c.**	Can you explain to me the time period the question was talking about?	
**1d.**	I asked about "**vitamin**" in my question. Can you explain to me what **"vitamin"** means in your own words?	
**1e.**	Could you tell me some examples of vitamins that women take during pregnancy?	
**1f.**	I asked **"were you given or did you buy".** Can you explain to me what **"were you given or did you buy"** means in your own words?	
**1g.**	Q1 INTERVIEWER NOTES	

**2**<br>**During your last pregnancy were you given or did you buy any of these vitamin tablets or syrups:**<br>E. IRON OR IRON AND FOLIC ACID?<br>F. PRENATAL?<br>G. FOLIC ACID?<br>H. ANY OTHER VITAMIN? (SPECIFY)<br>DO NOT SHOW VISUAL AID		YES	NO	DON'T KNOW
	e. Iron	1	2	8
	f. Prenatal	1	2	8
	g. Folic Acid	1	2	8
	h. Other vitamin	1	2	8
	SPECIFY Other vitamin			

	Q2. OBSERVATIONS<br>Note pause, facial expression, verbal responses, etc	
**2a.**	Where these questions easy or difficult for you to answer?<br>Why was it [EASY / DIFFICULT]?	
**2b.**	I asked "**During your last pregnancy were you given or did you buy any of these vitamin tablets or syrups**". In your own words can you tell me what I have just asked you?	
**2c.**	I asked about "iron or iron folic acid" in my question. Can you explain to me what "iron" means to you in your own words	
**2d.**	Can you tell me what " iron folic acid" means to you in your own words?	
**2e.**	I used the word "prenatal" in my question. Can you explain to me what "prenatal" mean in your own words?	
**2f.**	I used the word "folic acid" in my question. Can you explain to me what "folic acid" means in your own words?	
**2g.**	I used the words "any other vitamin" in my question. Can you explain to me what "any other vitamin" means in your own words?	
INTERVIEWER READ ALOUD: Now I am going to show you pictures and ask you the question again		
**2i.**	SHOW VISUAL AID OF IRON-3<br>**"During your last pregnancy were you given or did you buy any iron or iron-folic acid?"**<br>Do the pictures make it easier or more difficult to answer this question?	

	Why was it [EASIER/ MORE DIFFICULT]?	
2j.	SHOW VISUAL AID OF PRENATAL-3<br>**"During your last pregnancy were you given or did you buy any prenatal?"**<br>Do the picture make it easier or more difficult to answer this question?<br>Why was it [EASIER/ MORE DIFFICULT]?	
2k.	SHOW VISUAL AID – FOLIC ACID-3<br>**"During your last pregnancy were you given or did you buy any folic acid?"**<br>Do the pictures make it easier or more difficult to answer this question?<br>Why was it [EASIER/ MORE DIFFICULT]?	
2l.	Q2 INTERVIEWER NOTES	

7.	**During your last pregnancy where did you get the vitamin tablet or syrups? Anywhere else?**<br><br>PROBE TO IDENTIFY THE TYPE OF SOURCE. IF UNABLE TO DETERMINE IF PUBLIC, PRIVATE, OR NGO SECTOR, RECORD 'X' AND WRITE THE NAME OF THE PLACE(S).	1. Government hospitals<br>2. Health centers<br>3. Private hospitals<br>4. NGO/ Charity clinics<br>5. Private pharmacy<br>6. Public Pharmacy<br>7. Other, specify
	Q3 OBSERVATIONS<br><br>Note pause, facial expression, verbal responses, etc<br><br>Why was it [EASY/ DIFFICULT]?	
3a.	Was it easy or difficult **to remember where** you got the vitamin tablets or syrups?	
3b.	How did you remember where you got the vitamin tablets or syrup?	
3c.	IF Q2 RESPOND "YES" TO >1	Q2 yes to 1……..………………………SKIP

	Earlier you said that you got [IRON] Where do you get this?	Q2 yes to >1 …………………(explain)
3d.	IF Q2 RESPOND "YES" TO >1<br><br>Earlier you said that you got [PRENATAL] Where do you get this?	Q2 yes to 1………………………………SKIP<br>Q2 yes to >1 …………………(explain)
3e.	IF Q2 RESPOND "YES" TO >1<br><br>Earlier you said that you got [FOLIC ACID] Where do you get this?	Q2 yes to 1………………………………SKIP<br>Q2 yes to >1 …………………(explain)
3f.	IF Q2 RESPOND "YES" TO >1 Earlier you said that you got [OTHER VITAMIN] Where do you get this?	Q2 yes to 1………………………………SKIP<br>Q2 yes to >1 …………………(explain)
3g.	Q3 INTERVIEWER NOTES	

8.	**During your last pregnancy, how many months did you take vitamin tablets or syrup?**<br><br>**IF ANSWER IS NOT NUMERIC, PROBE FOR APPROXIMATE NUMBER OF DAYS.**	[ ][ ] MONTHS<br>98 = Does not know
	Q4 OBSERVATIONS<br><br>Note pause, facial expression, verbal responses, etc	
4a.	Was it easy or difficult to remember how many months you took a vitamin tablet or syrup?<br><br>Why was it [EASY/DIFFICULT]?	
4b.	How did you come up with the answer of months?	
4c.	You said months, how sure are you of that?	

**4d.**	IF Q2 RESPOND "YES" TO >1 A-D<br><br>Earlier you said that you got [IRON] how many **months** did you take [ ]?	Q2 yes to 1…………………………………SKIP<br>Q2 yes to >1 ………………….(explain)
**4e.**	IF Q2 RESPOND "YES" TO >1 A-D<br><br>Earlier you said that you got [PRENATAL] how many **months** did you take [ ]?	Q2 yes to 1…………………………………SKIP<br>Q2 yes to >1 ………………….(explain)
**4f.**	IF Q2 RESPOND "YES" TO >1 A-D<br><br>Earlier you said that you got [[FOLIC ACID] how many **months** did you take [ ]?	Q2 yes to 1…………………………………SKIP<br>Q2 yes to >1 ………………….(explain)
**4g.**	IF Q2 RESPOND "YES" TO >1 A-D<br><br>Earlier you said that you got [[OTHER VITAMINS] how many **months** did you take [ ]?	Q2 yes to 1…………………………………SKIP<br>Q2 yes to >1 ………………….(explain)
**4h.**	Q4 INTERVIEWER NOTES	

**5.**	**During your last pregnancy, how many weeks or months pregnant were you when you first started taking a vitamin tablet or syrup?**<br><br>**IF ANSWER IS NOT NUMERIC, PROBE FOR APPROXIMATE NUMBER OF DAYS.**	1. Weeks [ ][ ]<br>2. Months [ ][ ]<br>98. Don't know
	Q5 OBSERVATIONS<br><br>Note pause, facial expression, verbal responses, etc	
**5a.**	Was this question easy or difficult for you to answer?<br><br>Why was it [EASY/ DIFFICULT]?	

Data DENT
Data for Decisions in Nutrition

Addis Continental Institute of Public Health

**5b.**	I used the words "**how many weeks or <u>months</u> <u>pregnant</u>**". Can you explain to me what **<u>"how many months pregnant"</u>** means in your own words?	
**5c**	You said you started first started taking vitamins when you were weeks or months pregnant. How did you come up with the answer of weeks or months?	
**5d.**	You said you first started taking when you were weeks or months pregnant, how sure are you of that?	
**5e.**	IF Q2 RESPOND "YES" TO >1<br><br>Earlier you said that you got [IRON] how many **<u>weeks or months pregnant</u>** were you when you **<u>first started</u>** taking [IRON]?	Q2 yes to 1……………………………….SKIP<br>Q2 yes to >1 ………………<br>1. Weeks [ ][ ]<br>2. Months [ ][ ]<br>98. Don't know
**5f.**	IF Q2 RESPOND "YES" TO >1<br><br>Earlier you said that you got [PRENATAL] how many **<u>weeks or months pregnant</u>** were you when you **<u>first started</u>** taking [PRENTAL]?	Q2 yes to 1……………………………….SKIP<br>Q2 yes to >1 ………………<br>1. Weeks [ ][ ]<br>2. Months [ ][ ]<br>98. Don't know
**5g.**	IF Q2 RESPOND "YES" TO >1<br><br>Earlier you said that you got [[FOLIC ACID] how many **<u>weeks or months pregnant</u>** were you when you **<u>first started</u>** taking [FOLIC ACID]?	Q2 yes to 1……………………………….SKIP<br>Q2 yes to >1 ………………<br>1. Weeks [ ][ ]<br>2. Months [ ][ ]<br>98. Don't know
**5h.**	IF Q2 RESPOND "YES" TO >1<br><br>Earlier you said that you got [[OTHER VITAMINS] how many **<u>weeks or months pregnant</u>** were you when you **<u>first started</u>** taking [FOLIC ACID]?	Q2 yes to 1……………………………….SKIP<br>Q2 yes to >1 ………………<br>1. Weeks [ ][ ]

		2. Months [ ][ ]<br>98. Don’t know
**5i.**	Q5 INTERVIEWER NOTES	

6.	**During your last pregnancy, how many weeks or months pregnant were you when you <u>stopped</u> taking a vitamin tablet or vitamin syrup?**<br><br>**IF ANSWER IS NOT NUMERIC, PROBE FOR APPROXIMATE NUMBER OF DAYS.**	1. Weeks [ ][ ]<br>2. Months [ ][ ] (0-10)<br><br>12 = After giving birth<br>98 = Does not know
	Q6 OBSERVATIONS<br><br>Note pause, facial expression, verbal responses, etc	
**6a.**	Was this question easy or difficult for you to answer?<br><br>Why was it <u>[EASY/ DIFFICULT]?</u>	
**6b**	You said you started stopped taking when you were weeks or months pregnant. How did you come up with the answer of weeks or months pregnant?	
**6c**	You said you were X weeks or months, how sure are you of that?	
**6d**	IF Q2 RESPOND “YES” TO >1 A-D<br><br>Earlier you said that you got [IRON] how many **<u>weeks or months pregnant</u>** were you when you **<u>stopped</u>** taking [IRON]?	
**6e.**	IF Q2 RESPOND “YES” TO >1 A-D<br><br>Earlier you said that you got [PRENATAL] how many **<u>weeks or months pregnant</u>** were you when you **<u>stopped</u>** taking [PRENTAL]?	
**6f.**	IF Q2 RESPOND “YES” TO >1 A-D	

	Earlier you said that you got [[FOLIC ACID] how many **weeks or months pregnant** were you when you **stopped** taking [FOLIC ACID]?	
6g.	IF Q2 RESPOND "YES" TO >1 A-D<br><br>Earlier you said that you got [[OTHER VITAMINS] how many **weeks or months pregnant** were you when you **stopped** taking [FOLIC ACID]?	
6h.	Q6 INTERVIEWER NOTES	

7.	**During your last pregnancy, when you had vitamin tablet or syrup at home, how often did you take them? (Read responses)**	Daily ....1<br>A few days a week ....2<br>One day a week ....3<br>A few days a month ....4<br>One day per month or fewer…………..5<br>Other (specify)………………………… ………………6
	Q7 OBSERVATIONS<br><br>Note pause, facial expression, verbal responses, etc	
7a.	Was it easy or difficult to decide what answer to choose?<br><br>Why was it [EASY/ DIFFICULT]?	
7b.	I said "**when you had vitamin tablet or syrup at home**". Can you explain to me what "**when you had vitamin tablet or syrup at home**" means to you in your own words?	
7c.	I asked "**how often did you take**" in my question. Can you explain to me what "**how often did you take**" means to you in your own words?	
7d.	How did you come up with the answer of ?	
7e.	I used the word "**daily**". Can you explain to me what **"daily"** means to you in your own words?	
7f.	I said "**a few days per week**". Can you explain to me what **"a few days per week"** means to you in your own words?	

	Probe: How many days is "a few days per week"?	
**7g.**	I said "**a few days a month**". Can you explain to me what "a few days a month" means to you in your own words?<br><br>Probe: How many days is "**a few days a month**"?	
**7h.**	Q7 INTERVIEWER NOTES	

**8.**	**During the whole pregnancy, for how many days did you take vitamin tablets or vitamin syrup?**<br><br>**IF ANSWER IS NOT NUMERIC, PROBE FOR APPROXIMATE NUMBER OF DAYS.**	[ ][ ][ ] NUMBER OF DAYS (0-350)<br>998 = Does not know
	Q8 OBSERVATIONS<br><br>Note pause, facial expression, verbal responses, etc	
**8a.**	Was it easy or difficult is it to remember how many days you took a vitamin tablet or syrup for your whole pregnancy?<br><br>Why was it [EASY/ DIFFICULT]?	
**8b.**	You said you took for days. How did you come up with the answer of days?	
**8c.**	You said you took for days, How sure are you of that?	
**8d.**	Q8 INTERVIEWER NOTES	

Data DENT
Data for Decisions in Nutrition

Addis Continental Institute of Public Health

Text S8: Round 1 Cognitive Interview Module 4, Ethiopia

MODULE 4. VISUAL AID

INTERVIEWER READ ALOUD: Now I would like to ask you some more questions about the pictures we showed you earlier. We can make changes to these pictures so they are more attractive and more helpful. We will ask you questions about how you see these pictures and how we can make them better.

1. IRON / IRON FOLIC ACID: SHOW IRON-3 AS YOU ASK THE FOLLOWING QUESTIONS		
**1a**	What products do you recognize in this picture?<br><br>IF "NONE" SKIP TO 1C	
**1b**	Have you ever been given or purchased any of the products in this picture? If yes, which ones?<br><br>RECORD LETTERS	
**1c**	Have you ever been given or purchased any of the products that are similar to these but is not pictured here?<br><br>If yes, what is the name product(s)?	Yes……………………………………………1<br>No……………………………………………2
**1d**	All three products in this picture provide the same types of contents. Can you explain to me what <u>contents all the products</u> have?	
**1e**	What in the pictures helps you identify what the products are and what contents they contain?<br><br>Probe: Did you recognize packaging box, bottle, strips, or pill?	
**1f.**	Was it easy or difficult to see and identify the products in the picture?<br><br>Why was it <u>[EASY/ DIFFICULT]</u>?	
**1g.**	There are words printed on the product boxes. Are you reading those words?<br><br>*<u>Probe:</u>* For you personally, is it important to read the words on the products to identify what the product is?<br><br>*<u>Probe:</u>* If yes, are the words on the box visible to you? Are they large/clear enough to read?	.

**1h**	How should the picture be changed to make it easier to see and identify the type of products?<br><br>*Probe:* Change the font? Change the size? Add labels/names to product? Other ideas?	
**1i**	We want women to see these images and think about "iron or iron folic acid".<br><br>Probe: Is there a product we should add to this picture to make it clearer these are all examples of "iron or iron folic acid"?<br><br>Probe: Is there are product we should remove from picture to make it clear that these are all examples of "iron or iron folic acid?	
**1j**	SHOW IRON-3<br><br>Is there anything else you want to say about these pictures?	

2. PRENATAL : SHOW PRENATAL-3 AS YOU ASK THE FOLLOWING QUESTIONS		
**2a**	What products do you recognize in this picture?<br><br>IF "NONE" SKIP TO 2C	
**2b**	Have you ever been given or purchased any of the products in this picture? If yes, which ones?<br><br>RECORD LETTERS	Yes……………………………………………1<br>No……………………………………………2<br><br>If yes, which? (
**2c**	Have you ever been given or purchased any of the products that are similar to these but is not pictured here?<br><br>If yes, what is the name product(s)?	Yes……………………………………………1<br>No……………………………………………2
**2d**	All three products in this picture provide the same types of contents. Can you explain to me what contents all the products have?	
**2e**	What in the pictures helps you identify what the products are and what contents they contain?<br><br>Probe: Did you recognize packaging box, bottle, strips, or pill?	

**2f.**	Was it easy or difficult to see and identify the products in the picture?<br><br>Why was it [EASY/ DIFFICULT]?	
**2g.**	There are words printed on the product boxes. Are you reading those words?<br><br>*Probe:* For you personally, is it important to read the words on the products to identify what the product is?<br><br>*Probe:* If yes, are the words on the box visible to you? Are they large/clear enough to read?	
**2h**	How should the picture be changed to make it easier to see and identify the type of products?<br><br>*Probe:* Change the font? Change the size? Add labels/names to product? Other ideas?	
**2i.**	REFER TO ANSWER IN MODULE 3 Q2e.<br><br>Earlier in our discussion you said that a prenatal is , is that correct?<br><br>If no,: What does the word "prenatal" mean to you?	Yes, correct ....1<br>No, not correct 2
**2j.**	Do you consider all the products shown here to be "prenatals"?<br><br>If no, show me which products are prenatals. (NOTE LETTER)<br><br>*Probe:* why are products they did not select NOT a prenatal?	Yes...1<br>No..2
**2k.**	We want women to see these images and think about supplements for pregnant women that contain many different vitamins<br><br>What word or phrase would you use to talk about "supplements for pregnant women that contain many different vitamins?<br><br>Probe: Are there other names you would give to this type of product	

**2l.**	Probe: Is there a product we should add to this picture to make it clearer these are all examples of "supplements that contain many different vitamins"?<br><br>Probe: Is there are product we should remove from picture to make it clear that these are all examples of "supplements that contain many different vitamins?	
**2m.**	Is there anything else you want to say about these pictures?	

3. FOLIC ACID : SHOW FOLIC ACID AS YOU ASK THE FOLLOWING QUESTIONS		
**3a**	What products do you recognize in this picture?<br><br>IF "NONE" SKIP TO 3C	
**3b**	Have you ever been given or purchased any of the products in this picture? If yes, which ones?<br><br>RECORD LETTERS	Yes……………………………………………1<br>No…………………………………………….2<br><br>If yes, which? (
**3c**	Have you ever been given or purchased any of the products that are similar to these but is not pictured here?<br><br>If yes, what is the name product(s)?	Yes……………………………………………1<br>No…………………………………………….2
**3d**	All three products in this picture provide the same types of contents. Can you explain to me what contents all the products have?	
**3e**	What in the pictures helps you identify what the products are and what contents they contain?<br><br>Probe: Did you recognize packaging box, bottle, strips, or pill?	
**3f.**	Was it easy or difficult to see and identify the products in the picture?<br><br>Why was it [EASY/ DIFFICULT]?	

3g.	There are words printed on the product boxes. Are you reading those words?<br><br>*Probe:* For you personally, is it important to read the words on the products to identify what the product is?<br><br>*Probe:* If yes, are the words on the box visible to you? Are they large/clear enough to read?	
3h.	We want women to see these images and think about "folic acid"<br><br>Probe: Is there a product we should add to this picture to make it clearer these are all examples of "folic acid"?<br><br>Probe: Is there are product we should remove from picture to make it clear that these are all examples of "folic acid"?	
3i	Is there anything else you want to say about these pictures?	

4. VITAMIN : SHOW VITAMIN -5 AS YOU ASK THE FOLLOWING QUESTIONS		
4a.	This picture has products selected from all of the other pictures you have seen.<br><br>What word or phrase would you use to describe all of the products on this card?<br><br>Anything else?	.
4b	Some people use the word "Supplement" for products like these.<br><br>What does the term "supplement" mean to you?<br><br>Which of these products, if any, would you consider a supplement?	
4c	Some people use the word "vitamin" for products like these.<br><br>What does the term "vitamin" mean to you?<br><br>Which of these products, if any, would you consider a vitamin?	

Text S9: Round 2 Cognitive Interview CPW Ethiopia

PHASE II: COGNITIVE INTERVIEW WITH CURRENTLY PREGNANT WOMEN

MODULE 1. IDENTIFICATION

IDENTIFICATION	RESPONSE/CODE
NAME OF THE HEALTH FACILITY [Only to fill in if conducted at health facility]	
NAME OF THE SUB-CITY	
NAME OF THE WOREDA	
CURRENTLY PREGNANT OR RECENTLY DELIVERED	1. CURRENTLY PREGNANT<br>2. RECENTLY DELIVERED
MOTHER ID#	
PRIMARY LANGUAGE SPOKEN	
MOBILE NUMBER WHERE RESPONDENT CAN BE REACHED	
**INTERVIEWER INFORMATION**	**CODE**
CODE OF THE INTERVIEWER	
CODE OF THE NOTE-TAKER/SECOND INTERVIEWER	
DATE OF THE INTERVIEW	[ ][ ] : [ ][ ]<br>DAY<br>MONTH
START TIME (24-HOUR CLOCK)	[ ][ ] : [ ][ ]<br>HR MIN
END TIME (24-HOUR CLOCK)	[ ][ ] : [ ][ ]<br>HR MIN
DURATION (HOUR/MIN)	[ ][ ] : [ ][ ]<br>HR MIN
**RESULT CODES:**	
COMPLETE...1<br>PARTIALLY COMPLETE 2	REFUSAL.. 3<br>ELIGIBLE RESPONDENT AWAY FOR EXTENDED PERIOD.. 4

Data DENT
ta for Decisions in Nutrition

Addis Continental Institute of Public Health

MODULE 2. CURRENTLY PREGNANT RESPONDENT'S BACKGROUND AND PREGNANCY HISTORY

1.	How old are you? Can you tell me what month and year were you born?	Month [ ][ ]<br>98. Don't know month<br>Year [ ][ ][ ][ ]<br>99. Don't know year
2.	How old were you at your last birthday? (response required)	Age in completed years [ ][ ]
3.	What is the highest grade or number of years you have completed at school?	GRADE/YEARS [ ][ ]<br>98. No schooling
4.	Have you lived in Addis Ababa continuously for the last year?	7. Yes → 5<br>8. No
4a.	If no, when did you come to Addis Ababa?	[ ][ ] [ ][ ]][ ]<br>MONTH YEAR<br>98. Don't know
5.	Now I would like to ask about all the births you have had during your life. Have you ever given birth? **[If no, SKIP to Q7.]**	3. Yes<br>4. No
6.	How many total births have you had? (ask for the total number of births even if did not result in a live birth)?	Number of total births [ ][ ]
7.	How old is your youngest child?	10. Weeks [ ][ ]<br>11. Months [ ][ ]<br>12. Year [ ][ ]
8.	How many weeks or months pregnant are you?	7. Weeks [ ][ ]<br>8. Months [ ][ ]
9.	Did you receive antenatal care during this pregnancy? **[If no, END INTERVIEW]**	7. No<br>8. Yes<br>98. Don't Know
10	Where did you receive antenatal care for this pregnancy? Anywhere else?<br><br>RECORD ALL SOURCES. PROBE TO IDENTIFY TYPE OF SOURCE. IF UNABLE TO DETERMINE IF PUBLIC, PRIVATE, OR NGO SECTOR, RECORD 'X' AND WRITE THE NAME OF THE PLACE(S).	37. Home/ Her home<br>38. Other home<br>39. Government hospital<br>40. Government health center<br>41. Other public sector (specify)<br>42. Private hospital<br>43. Private clinic

		44. Other private medical sector (specify)<br>45. NGO hospital<br>46. NGO clinic<br>47. Other NGO medical sector (specify)<br>48. Other, (specify)
11.	Whom did you see?<br><br>PROBE TO IDENTIFY EACH TYPE OF PERSON AND RECORD ALL MENTIONED.	22. Doctor<br>23. Nurse/midwife<br>24. Health officer<br>25. Traditional birth attendant<br>26. Health Extension Workers<br>27. Not able to differentiate<br>28. Other, (specify)
12.	How many weeks or months pregnant were you when you first received antenatal care for this pregnancy?	8. Weeks [ ][ ]<br>9. Months [ ][ ]<br>98. Don't know
13.	How many times have you received antenatal care during this pregnancy?	Number of times [ ][ ]<br>98. Don't know

MODULE 3. MICRONUTRIENT SUPPLEMENTATION QUESTIONS

INTERVIEWER READ ALOUD: Today I will be asking you questions about products for pregnant women. We would like to know if you understand the questions and how you understand them. The questions we are about to ask you might seem simple or repetitive. But your responses are important to us. There are also no right or wrong answers.

			YES	NO	DON'T KNOW
1	**During this pregnancy have you been given or have you bought any of these vitamin tablets or syrups:**<br>I. IRON OR IRON AND FOLIC ACID?<br>J. PRENATAL?<br><br>SHOW VISUAL AID	i. Iron<br>j. Prenatal	1<br>1	2<br>2	8<br>8

	Q1. OBSERVATIONS<br>Note pause, facial expression, verbal responses, etc	
**1a.**	Where these questions easy or difficult for you to answer?<br><br>Why was it [EASY / DIFFICULT]?	
**1b.**	I asked "**During this pregnancy have you been given or have you bought any of these vitamin tablets or syrups**" In your own words can you tell me what I have just asked you?	
**1c.**	I asked about "iron or iron folic acid" in my question. Can you explain to me what "iron" means to you in your own words	
**1d.**	Can you tell me what " iron folic acid" means to you in your own words?	
**1e.**	I used the word "prenatal" in my question. Can you explain to me what "prenatal" mean in your own words?	
**1f.**	Q1 INTERVIEWER NOTES	

**2.**	**During this pregnancy where do you get the vitamin tablets or syrup? Anywhere else?**<br><br>PROBE TO IDENTIFY THE TYPE OF SOURCE. IF UNABLE TO DETERMINE IF PUBLIC, PRIVATE, OR	8. Government hospitals<br>9. Health centers<br>10. Private hospitals<br>11. NGO/ Charity clinics<br>12. Private pharmacy

	NGO SECTOR, RECORD 'X' AND WRITE THE NAME OF THE PLACE(S).	13. Public Pharmacy<br>14. From overseas<br>15. Other, specify
	Q2 OBSERVATIONS<br><br>Note pause, facial expression, verbal responses, etc	
**2a.**	Was it easy or difficult to remember **where you got** the vitamin tablets or syrups?<br><br>Why was it [EASY/ DIFFICULT]?	
**2b.**	Q2 INTERVIEWER NOTES	

**3.**	**During this pregnancy, how many** months pregnant **were you when you first started taking these vitamin tablet or syrup?**	3. Months [ ][ ]<br>98. Don't know
	Q3 OBSERVATIONS<br><br>Note pause, facial expression, verbal responses, etc	
**3a.**	Was this question easy or difficult for you to answer?<br><br>Why was it [EASY/ DIFFICULT]?	
**3b.**	I used the words "**how many months pregnant**". Can you explain to me what "**how many months pregnant**" means in your own words?	
**3c**	You said you first started taking vitamins when you were months pregnant. How did you come up with the answer of months?	
**3d.**	You said you first started taking when you were months pregnant, how sure are you of that?	
**3e.**	IF Q1 RESPOND "YES" TO >1<br><br>Earlier you said that you got [IRON] how many **months pregnant** were you when you **first started** taking [IRON]?	Q1 yes to 1……..……………………….SKIP<br>Q1 yes to >1 ………………<br>3. Months [ ][ ]<br>98. Don't know
**3f.**	IF Q1 RESPOND "YES" TO >1	Q1 yes to 1……..……………………….SKIP

	Earlier you said that you got [PRENATAL/MMS] how many **months pregnant** were you when you **first started** taking [PRENTAL/MMS]?	Q1 yes to >1 ………………<br>3. Months [ ][ ]<br>98. Don't know
**3f.**	IF Q1 RESPOND "YES" TO >1 A-B<br><br>Did you ever take both a iron tablet or syrup and a prenatal tablet on the same day?<br><br>Probe: If yes, please explain. For how much time did you take both supplements/vitamins on the same day?	
**3g.**	Q3 INTERVIEWER NOTES	

**4.**	**In the last week, how many days did you take this vitamin tablet or syrup?**<br><br>**IF ANSWER IS NOT NUMERIC, PROBE FOR APPROXIMATE NUMBER OF DAYS.**	[ ] NUMBER OF DAYS (0-7)<br>998 = Does not know
	Q4 OBSERVATIONS<br><br>Note pause, facial expression, verbal responses, etc	
**4a.**	Was it easy or difficult is it to remember how many days in the last week you took a vitamin tablet or syrup?<br><br>Why was it [EASY/ DIFFICULT]?	
**4b**	Can you explain to me the time period the question was talking about?	
**4c.**	You said you took for days, how did you come up with the answer of days?	
**4d.**	You said you took for days, How sure are you of that?	
**4e.**	Q7 INTERVIEWER NOTES	

**5.**	**During the last month, for how many days did you take these vitamin tablet or syrup?**<br><br>**IF ANSWER IS NOT NUMERIC, ASK FOR THE APPROXIMATE NUMBER OF DAYS THEY TOOK THE TABLET OR SYRUP.**	[ ][ ]NUMBER OF DAYS (0-31)<br>998 = Does not know

	Q5 OBSERVATIONS<br><br>Note pause, facial expression, verbal responses, etc	
**5a.**	Was it easy or difficult to remember how many days you took a vitamin tablet or syrup over the last month?<br><br>Why was it [EASY/ DIFFICULT]?	
**5b.**	I asked "**during the last month**" How did you count "**during the last month**"?<br><br>Probe: People think of a month in different ways. Did you think about the [LAST HOLIDAY/ LAST ANC VISIT/ LAST 30 DAYS]?	
**5c.**	I asked "**how many days did you take**" Can you explain to me what **"take"** means in your own words?<br><br>Probe: For you, does take mean that you consume the tablet or syrup or does it mean that you received the tablet?	
**5d.**	You said you took for days, How did you come up with the answer days?	
**5e.**	You said you took it for days, How sure are you of that?	
**5f.**	Q5 INTERVIEWER NOTES	

**6.**	**During the last month, how often did you take these vitamin tablets or syrups? (Read responses)**	Daily ....1<br>A few days a week ....2<br>One day a week ....3<br>A few days a month ....4<br>One day per month or fewer…………..5<br>Other (specify)………………………… ……………….6
**6a.**	Q6 OBSERVATIONS<br><br>Note pause, facial expression, verbal responses, etc	
**6b**	Was it easy or difficult tocome up with a response?<br><br>Why was it [EASY/ DIFFICULT]?	
**6c.**	I asked "**how often did you take**" in my question. Can you explain to me what "**how often did you take**" means to you in your own words?	

**6d.**	How did you come up with the answer of ?	
**6e.**	**During your last pregnancy, how often did these vitamin tablets or syrups? [Record response word for word]**	
**6e.**	Q7 INTERVIEWER NOTES	
**7.**	**During the last month, were there days when you did not take the tablet or syrup?**	YES . . . . 1<br>NO . . . . 2<br>DON'T KNOW 8
	**IF YES →, how often did you miss taking the tablet or syrup?**	Daily ....1<br>A few days a week ....2<br>One day a week ....3<br>A few days a month ....4<br>One day per month or fewer…………5<br>Other (specify)………………………………………6
	Q7 OBSERVATIONS<br><br>Note pause, facial expression, verbal responses, etc	
**7a.**	Was it easy or difficult to decide what answer to choose?<br><br>Why was it [EASY/ DIFFICULT]?	
**7b.**	I said "**you did not take**". Can you explain to me what "**you did not take**" means to you in your own words?	
**7c.**	If you did miss a day, what caused you to miss the day. [sometimes women miss a day because the tablet or syrup upsets their stomach/they forget/their routine is different]	
**7d.**	How did you come up with your answer?	
**7e.**	Did you feel pressure to give a certain answer whether or not that was true for you?<br><br>If yes, why did you feel pressure?	
**7f.**	Q7 INTERVIEWER NOTES	

Data DENT
ta for Decisions in Nutrition

Addis Continental Institute of Public Health

Text S10: Round 2 Cognitive Interview, RDW, Ethiopia

PHASE II: COGNITIVE INTERVIEW WITH RECENTLY DELIVERED WOMEN

MODULE 1. IDENTIFICATION

**IDENTIFICATION**	**RESPONSE/CODE**
NAME OF THE HEALTH FACILITY [Only to fill in if conducted at health facility]	
NAME OF THE SUB-CITY	
NAME OF THE WOREDA	
CURRENTLY PREGNANT OR RECENTLY DELIVERED	1. CURRENTLY PREGNANT<br>2. RECENTLY DELIVERED
NAME OF MOTHER	
MOTHER ID#	
PRIMARY LANGUAGE SPOKEN	
MOBILE NUMBER WHERE RESPONDENT CAN BE REACHED	
**INTERVIEWER INFORMATION**	**CODE**
CODE OF THE INTERVIEWER	
CODE OF THE NOTE-TAKER/SECOND INTERVIEWER	
DATE OF THE INTERVIEW	[ ][ ] [ ][ ] DAY MONTH
START TIME (24-HOUR CLOCK)	[ ][ ] : [ ][ ] HR MIN
END TIME (24-HOUR CLOCK)	[ ][ ] : [ ][ ] HR MIN
DURATION (HOUR/MIN)	
**RESULT CODES:**<br>COMPLETE....1<br>PARTIALLY COMPLETE....2	REFUSAL....3<br>ELIGIBLE RESPONDENT AWAY FOR EXTENDED PERIOD....4

MODULE 2. RECENTLY DELIVERED RESPONDENT'S BACKGROUND AND PREGNANCY HISTORY

1.	How old are you? Can you tell me what month and year were you born?	Month [ ][ ]<br>98. Don't know month<br>Year [ ][ ][ ][ ]<br>99. Don't know month
2.	How old were you at your last birthday? (response required)	Age in completed years [ ][ ]
3.	What is the highest grade or number of years you have completed at school?	GRADE/YEARS [ ][ ]<br>98. No schooling
4.	Have you lived in Addis Ababa continuously for the last year?	3. Yes → Skip to 5<br>4. No
4a.	If no, when did you come to Addis Ababa?	[ ][ ] [ ][ ]][ ]<br>MONTH YEAR<br>98. Don't know
5.	Now I would like to ask about all the births you have had during your life. Have you ever given birth?<br>**[If no, END INTERVIEW]**	7. Yes<br>8. No
6.	How many total births have you given? (ask for the total number of births even if did not result in a live birth)?	Number of total births [ ][ ]
7.	How old is your youngest child?	10. Weeks [ ][ ]<br>11. Months [ ][ ]<br>12. Year [ ][ ]
8.	Did you see anyone for antenatal care during your most recent pregnancy? **[If no, END INTERVIEW]**	7. No<br>8. Yes<br>98. Don't Know
9.	Where did you receive antenatal care during your most recent pregnancy? Anywhere else?<br><br>RECORD ALL SOURCES. PROBE TO IDENTIFY TYPE OF SOURCE. IF UNABLE TO DETERMINE IF PUBLIC, PRIVATE, OR NGO SECTOR, RECORD 'X' AND WRITE THE NAME OF THE PLACE(S).	37. Home/ Her home<br>38. Other home<br>39. Government hospital<br>40. Government health center<br>41. Other public sector (specify)<br>42. Private hospital<br>43. Private clinic<br>44. Other private medical sector (specify)<br>45. NGO hospital<br>46. NGO clinic<br>47. Other NGO medical sector (specify)<br>48. Other, (specify)
	Whom did you see? Anyone else?	22. Doctor

	PROBE TO IDENTIFY EACH TYPE OF PERSON AND RECORD ALL MENTIONED.	23. Nurse/midwife<br>24. Health officer<br>25. Traditional birth attendant<br>26. Health Extension Workers<br>27. Not able to differentiate<br>28. Other (specify)
10.	How many weeks or months pregnant were you when you first received antenatal care for your most recent pregnancy?	9. Weeks [ ][ ]<br>10. Months [ ][ ]<br>98. Don't know
11.	How many times did you received antenatal care during your most recent pregnancy?	Number of times [ ][ ]<br>98. Don't know

Data DENT
ta for Decisions in Nutrition

Addis Continental Institute of Public Health

MODULE 3. MICRONUTRIENT SUPPLEMENTATION QUESTIONS

INTERVIEWER READ ALOUD: Today I will be asking you questions about products for pregnant women. We would like to know if you understand the questions and how you understand them. The questions we are about to ask you might seem simple or repetitive. But your responses are important to us. There are also no right or wrong answers.

		YES	NO	DON'T KNOW
1. **During your last pregnancy were you given or did you buy any of these vitamin tablets or syrups:**<br>K. IRON OR IRON AND FOLIC ACID?<br>L. PRENATAL?<br>SHOW VISUAL AID	k. Iron<br>l. Prenatal<br>m.	1<br>1	2<br>2	 8<br>8

	Q1. OBSERVATIONS<br>Note pause, facial expression, verbal responses, etc	
**1a.**	Where these questions easy or difficult for you to answer?<br>Why was it [EASY / DIFFICULT]?	
**1b.**	I asked "**During your last pregnancy were you given or did you buy any of these vitamin tablets or syrups**". In your own words can you tell me what I have just asked you?	
**1c.**	I asked about "iron or iron folic acid" in my question. Can you explain to me what "iron" means to you in your own words	
**1d.**	Can you tell me what " iron folic acid" means to you in your own words?	
**1e.**	I used the word "prenatal" in my question. Can you explain to me what "prenatal" mean in your own words?	
**1f.**	Q1 INTERVIEWER NOTES	

**2.**	**During your last pregnancy where did you get the vitamin tablet or syrups? Anywhere else?**<br><br>PROBE TO IDENTIFY THE TYPE OF SOURCE. IF UNABLE TO DETERMINE IF PUBLIC, PRIVATE, OR NGO SECTOR, RECORD 'X' AND WRITE THE NAME OF THE PLACE(S).	16. Government hospitals<br>17. Health centers<br>18. Private hospitals<br>19. NGO/ Charity clinics<br>20. Private pharmacy<br>21. Public Pharmacy<br>22. From overseas<br>23. Other, specify
	Q2 OBSERVATIONS<br><br>Note pause, facial expression, verbal responses, etc	
**2a.**	Was it easy or difficult **to remember where** you got the vitamin tablets or syrups?<br><br>Why was it [EASY/ DIFFICULT]?	
**2b.**	Q2 INTERVIEWER NOTES	

**3.**	**During your last pregnancy, how many months did you take these vitamin tablets or syrup?**<br><br>**IF ANSWER IS NOT NUMERIC, PROBE FOR APPROXIMATE NUMBER OF DAYS.**	[ ][ ] MONTHS<br>98 = Does not know
	Q3 OBSERVATIONS<br><br>Note pause, facial expression, verbal responses, etc	
**3a.**	Was it easy or difficult to remember how many months you took a vitamin tablet or syrup?<br><br>Why was it [EASY/DIFFICULT]?	
**3b.**	How did you come up with the answer of months?	
**3c.**	You said months, how sure are you of that?	
**3d.**	IF Q1 RESPOND “YES” TO >1 A-B	Q1 yes to1…………………………….SKIP

	Earlier you said that you got [IRON] how many **months** did you take [IRON]?	Q1 yes to >1 ………………….(explain)
**3e.**	IF Q1 RESPOND "YES" TO >1 A-B<br><br>Earlier you said that you got [PRENATAL] how many **months** did you take [PRENATAL]?	Q1 yes to 1……..……………………………SK IP<br>Q1 yes to >1 ………………….(explain)
**3f.**	IF Q1 RESPOND "YES" TO >1 A-B<br><br>Did you ever take both a iron tablet or syrup and a prenatal tablet on the same day?<br><br>Probe: If yes, please explain. For how much time did you take both supplements/vitamins on the same day?	
**3g.**	Q3 INTERVIEWER NOTES	

**4.**	**During your last pregnancy, how many months pregnant were you when you first started taking a vitamin tablet or syrup?**<br><br>**IF ANSWER IS NOT NUMERIC, PROBE FOR APPROXIMATE NUMBER OF DAYS.**	4. Months [ ][ ]<br>98. Don't know
	Q4 OBSERVATIONS<br><br>Note pause, facial expression, verbal responses, etc	
**4a.**	Was this question easy or difficult for you to answer?<br><br>Why was it [EASY/ DIFFICULT]?	
**4b.**	I used the words "**how many months pregnant**". Can you explain to me what **"how many months pregnant"** means in your own words?	
**4c**	You said you started first started taking vitamins when you were months pregnant. How did you come up with the answer of months?	
**4d.**	You said you first started taking when you were months pregnant, how sure are you of that?	
**4e.**	IF Q1 RESPOND "YES" TO >1<br><br>Earlier you said that you got [IRON] how many **months pregnant** were you when you **first started** taking [IRON]?	Q1 yes to 1……..……………………………SK IP<br>Q1 yes to >1 ………………

		4. Months [ ][ ]<br>98. Don't know
**4f.**	IF Q1 RESPOND "YES" TO >1<br><br>Earlier you said that you got [PRENATAL] how many **months pregnant** were you when you **first started** taking [PRENTAL]?	Q1 yes to 1……………………………SKIP<br>Q1 yes to >1 ……………<br>3. Months [ ][ ]<br>98. Don't know
**4g.**	Q4 INTERVIEWER NOTES	

5.	**During your last pregnancy, how many months pregnant were you when you stopped taking a vitamin tablet or vitamin syrup?**<br><br>**IF ANSWER IS NOT NUMERIC, PROBE FOR APPROXIMATE NUMBER OF DAYS.**	3. Months [ ][ ] (0-10)<br><br>12 = After giving birth<br>98 = Does not know
	Q5 OBSERVATIONS<br><br>Note pause, facial expression, verbal responses, etc	
**5a.**	Was this question easy or difficult for you to answer?<br><br>Why was it [EASY/ DIFFICULT]?	
**5b**	You said you started stopped taking when you were months pregnant. How did you come up with the answer of months pregnant?	
**5c**	You said you were X weeks or months, how sure are you of that?	
**5d**	IF Q1 RESPOND "YES" TO >1 A-B<br><br>Earlier you said that you got [IRON] how many **months pregnant** were you when you **stopped** taking [IRON]?	
**5e.**	IF Q1 RESPOND "YES" TO >1 A-B	

	Earlier you said that you got [PRENATAL] how many **months pregnant** were you when you **stopped** taking [PRENTAL]?	
**6f.**	Q5 INTERVIEWER NOTES	

**6.**	**During your last pregnancy, when you had a supply of these vitamin tablet or syrup, how often did you take them? [Read responses]**	Daily ....1<br>A few days a week ....2<br>One day a week ....3<br>A few days a month ....4<br>One day per month or fewer…………5<br>Other (specify)…………………………………………6
	Q6 OBSERVATIONS<br><br>Note pause, facial expression, verbal responses, etc	
**6a.**	Was it easy or difficult to decide what answer to choose?<br><br>Why was it [EASY/ DIFFICULT]?	
**6b.**	I said "**when you had a supply of this vitamin tablet or syrup**". Can you explain to me what "**when you had a supply of this vitamin tablet or syrup**" means to you in your own words?	
**6c.**	I asked "**how often did you take**" in my question. Can you explain to me what "**how often did you take**" means to you in your own words?	
**6d.**	How did you come up with the answer of ?	
**6e**	**During your last pregnancy, when you had a supply of these vitamin tablet or syrup, how often did you take them? [Record response word for word]**	
**6f.**	Q6 INTERVIEWER NOTES	

**7.**		YES . . . . 1<br>NO 2

	**During your last pregnancy, when you had a supply of these vitamin tablet or syrup, were there days when you did not take the tablet or syrup?**	DON'T KNOW 8
	**IF YES →, how often did you miss taking the tablet or syrup?**	Daily ....1<br>A few days a week ....2<br>One day a week ....3<br>A few days a month ....4<br>One day per month or fewer…………5<br>Other (specify)…………………………………………….6
	Q7 OBSERVATIONS<br><br>Note pause, facial expression, verbal responses, etc	
**7a.**	Was it easy or difficult to decide what answer to choose?<br><br>Why was it <u>[EASY/ DIFFICULT]?</u>	
**7b.**	I said "**were there days when you did not take**". Can you explain to me what "**were there days when you did not take**" means to you in your own words?	
**7c.**	If you did miss a day, what caused you to miss the day. [sometimes women miss a day because the tablet or syrup upsets their stomach/they forget/their routine is different]	
**7d.**	How did you come up with your answer?	
**7e.**	Did you feel pressure to give a certain answer whether or not that was true for you?<br><br>If yes, why did you feel pressure?	
**7f.**	Q7 INTERVIEWER NOTES	

Text S11: Round 2 Cognitive Interview, Module 4, Ethiopia

MODULE 4. VISUAL AID

INTERVIEWER READ ALOUD: Now I would like to ask you some more questions about the pictures we showed you earlier. We can make changes to these pictures so they are more attractive and more helpful. We will ask you questions about how you see these pictures and how we can make them better.

1. IRON / IRON FOLIC ACID: SHOW IRON-3 AS YOU ASK THE FOLLOWING QUESTIONS		
**1a**	What products do you recognize in this picture?<br><br>IF "NONE" SKIP TO 1C	
**1b**	Have you ever been given or purchased any of the products in this picture? If yes, which ones?<br><br>RECORD LETTERS	
**1c**	Have you ever been given or purchased any of the products that are similar to these but is not pictured here?<br><br>If yes, what is the name product(s)?<br><br>If yes, what about [name product] is similar to these other products?	Yes……………………………………………1<br>No……………………………………………..2<br><br>OPEN RESPONSE
**~~1d.~~**	Earlier when I showed you this picture (IRON-3) and I asked "**During your last pregnancy were you given or did you buy iron or iron folic acid"**<br><br>Did you think I was asking whether you took one of these specific three products in these specific packages or did you think of these and other products similar to these that are not specifically pictured?	
**1f**	SHOW IRON-3<br><br>Is there anything else you want to say about these pictures?	

2. PRENATAL: SHOW PRENATAL-3 AS YOU ASK THE FOLLOWING QUESTIONS		
**2a**	What products do you recognize in this picture?<br><br>IF "NONE" SKIP TO 2L	
**2b**	Have you ever been given or purchased any of the products in this picture? If yes, which ones?<br><br>RECORD LETTERS	Yes……………………………………………1<br>No……………………………………………..2<br><br>If yes, which?

**2c**	Have you ever been given or purchased any of the products that are similar to these but is not pictured here?<br><br>If yes, what is the name product(s)?<br><br>If yes, what about [name product] is similar to these other products?	Yes……………………………………………1<br>No……………………………………………..2<br><br>OPEN RESPONSE
**2d**	Earlier when I showed you this picture (PRENATAL-3) and I asked **"During your last pregnancy were you given or did you buy PRENATAL OR MMS"**<br><br>Did you think I was asking whether you took one of these three products in these specific packages or did you think of these and other products similar to these that are not specifically pictured?	
**2e**	Do you consider all the products shown here to be "prenatals"?<br><br>If no, show me which products are prenatals. (NOTE LETTER)<br><br>*Probe:* why are products they did not select NOT a prenatal?	Yes……………………………………………1<br>No……………………………………………..2
If they identified UNIMMAP ask the following:		
**2f**	Who gave you this product?	
**2g**	What name did that person call this product?<br><br>Probe: What name/word did they use?	
**2h**	Do you or other women use another name for this product?<br><br>Probe: What other name/word do you or other women use ? Any other?	
**2i**	Why should a pregnant woman take this product?<br><br>What does this product contain?	
**2j**	When you received this product how was it packaged?<br><br>Probe: packaged box, bottle, strip, plastic bag/ loose pills?	
**2k**	Is there anything else you want to say about these pictures?	

3. VITAMIN: SHOW VITAMIN -5 AS YOU ASK THE FOLLOWING QUESTIONS		
**3b**	Some people use the word "supplement" for products like these.<br><br>What does the term "supplement" mean to you?<br><br>Which of these products, if any, would you consider a supplement?	
**3c**	Some people use the word "vitamin" for products like these.<br><br>What does the term "vitamin" mean to you?<br><br>Is there a difference between a vitamin and as supplement?<br><br>Which of these products, if any, would you consider a vitamin?	

Text S12: Round 3 Cognitive Interview, CPW, Ethiopia

PHASE II: COGNITIVE INTERVIEW WITH CURRENTLY PREGNANT WOMEN

MODULE 1. IDENTIFICATION

IDENTIFICATION	RESPONSE/CODE
NAME OF THE HEALTH FACILITY [Only to fill in if conducted at health facility]	
NAME OF THE SUB-CITY	
NAME OF THE WOREDA	
CURRENTLY PREGNANT OR RECENTLY DELIVERED	1. CURRENTLY PREGNANT<br>2. RECENTLY DELIVERED
MOTHER ID#	
PRIMARY LANGUAGE SPOKEN	
MOBILE NUMBER WHERE RESPONDENT CAN BE REACHED	
**INTERVIEWER INFORMATION**	**CODE**
CODE OF THE INTERVIEWER	
CODE OF THE NOTE-TAKER/SECOND INTERVIEWER	
DATE OF THE INTERVIEW	[ ][ ] : [ ][ ]<br>DAY<br>MONTH
START TIME (24-HOUR CLOCK)	[ ][ ] : [ ][ ]<br>HR MIN
END TIME (24-HOUR CLOCK)	[ ][ ] : [ ][ ]<br>HR MIN
DURATION (HOUR/MIN)	[ ][ ] : [ ][ ]<br>HR MIN
**RESULT CODES:**	
COMPLETE....................1<br>PARTIALLY COMPLETE....................2	REFUSAL....................3<br>ELIGIBLE RESPONDENT AWAY FOR EXTENDED PERIOD....................4

Data DENT
ta for Decisions in Nutrition

Addis Continental Institute of Public Health

MODULE 2. CURRENTLY PREGNANT RESPONDENT'S BACKGROUND AND PREGNANCY HISTORY

1.	How old are you? Can you tell me what month and year were you born?	Month [ ][ ]<br>98. Don't know month<br>Year [ ][ ][ ][ ]<br>99. Don't know year
2.	How old were you at your last birthday? (response required)	Age in completed years [ ][ ]
3.	What is the highest grade or number of years you have completed at school?	GRADE/YEARS [ ][ ]<br>98. No schooling
4.	Have you lived in Addis Ababa continuously for the last year?	9. Yes → 5<br>10. No
4a.	If no, when did you come to Addis Ababa?	[ ][ ] [ ][ ][ ][ ]<br>MONTH YEAR<br>98. Don't know
5.	Now I would like to ask about all the births you have had during your life. Have you ever given birth? **[If no, SKIP to Q7.]**	5. Yes<br>6. No
6.	How many total births have you had? (ask for the total number of births even if did not result in a live birth)?	Number of total births [ ][ ]
7.	How old is your youngest child?	13. Weeks [ ][ ]<br>14. Months [ ][ ]<br>15. Year [ ][ ]
8.	How many weeks or months pregnant are you?	9. Weeks [ ][ ]<br>10. Months [ ][ ]
9.	Did you receive antenatal care during this pregnancy? **[If no, END INTERVIEW]**	9. No<br>10. Yes<br>98. Don't Know
10	Where did you receive antenatal care for this pregnancy? Anywhere else?<br><br>RECORD ALL SOURCES. PROBE TO IDENTIFY TYPE OF SOURCE. IF UNABLE TO DETERMINE IF PUBLIC, PRIVATE, OR NGO SECTOR, RECORD 'X' AND WRITE THE NAME OF THE PLACE(S).	49. Home/ Her home<br>50. Other home<br>51. Government hospital<br>52. Government health center<br>53. Other public sector (specify)<br>54. Private hospital<br>55. Private clinic<br>56. Other private medical sector (specify)<br>57. NGO hospital<br>58. NGO clinic

		59. Other NGO medical sector (specify)<br>60. Other, (specify)
11.	Whom did you see?<br><br>PROBE TO IDENTIFY EACH TYPE OF PERSON AND RECORD ALL MENTIONED.	29. Doctor<br>30. Nurse/midwife<br>31. Health officer<br>32. Traditional birth attendant<br>33. Health Extension Workers<br>34. Not able to differentiate<br>35. Other, (specify)
12.	How many weeks or months pregnant were you when you first received antenatal care for this pregnancy?	10. Weeks [ ][ ]<br>11. Months [ ][ ]<br>98. Don't know
13.	How many times have you received antenatal care during this pregnancy?	Number of times [ ][ ]<br>98. Don't know

Data DENT
Data for Decisions in Nutrition

Addis Continental Institute of Public Health

MODULE 3. MICRONUTRIENT SUPPLEMENTATION QUESTIONS

INTERVIEWER READ ALOUD: Today, I will be asking you questions about products for pregnant women. We would like to know if you understand the questions and how you understand them. The questions we are about to ask you might seem simple or repetitive. But your responses are important to us. There are also no right or wrong answers.

	**2. During this pregnancy were you given or did you buy any iron tablets or syrups?**<br><br>**DO NOT SHOW VISUAL AID**	YES . . . . …… . 1<br>NO . . . . 2<br>DON'T KNOW . . . .… 8
	Q1. OBSERVATIONS<br>Note pause, facial expression, verbal responses, etc.	
**1a.**	Where these questions easy or difficult for you to answer?<br>Why was it [EASY / DIFFICULT]?	
**1b.**	If I asked instead about "pill (kinin) for anemia" in my question. Can you explain to me what "pill (kinin) for anemia" means to you in your own words.<br>Is this "pill (kinin) for anemia" different than "iron tablet or syrup." Please explain.	
**1c.**	Q1 INTERVIEWER NOTES	

			YES	NO	DON'T KNOW
**2**	**During this pregnancy which iron tablets or syrups did you take…**<br>M. IRON WITH EXTRA VITAMINS OR PRENTATAL ?<br>N. IRON OR IRON FOLIC ACID?<br>SHOW VISUAL AIDS FOR EACH GROUP	n. Iron with extra vitamins or prenatal	1<br>1	2<br>2	8<br>8

	INTERVIEW READ ALOUD: Please think about these and similar products; the pictures are just examples	o. Iron or Iron Folic Acid			

	Q2. OBSERVATIONS<br>Note pause, facial expression, verbal responses, etc	
**2a.**	Where these questions easy or difficult for you to answer?<br>Why was it [EASY / DIFFICULT]?	
**2b.**	I asked about "iron with extra vitamins"" in my question. Can you explain to me what "iron with extra vitamins " means to you in your own words	
**2c.**	Can you name any of the extra vitamins that it contains?	
**2d.**	When I showed you the picture, I said: "Please think about these and similar products; the pictures are just examples." Can you explain what this means to you in your own words	
**2e.**	Q1 INTERVIEWER NOTES	

**3.**	**During this pregnancy where do you get iron tablets or syrup? Anywhere else?**<br>PROBE TO IDENTIFY THE TYPE OF SOURCE. IF UNABLE TO DETERMINE IF PUBLIC, PRIVATE, OR NGO SECTOR, RECORD 'X' AND WRITE THE NAME OF THE PLACE(S).	24. Government hospitals<br>25. Health centers<br>26. Private hospitals<br>27. NGO/ Charity clinics<br>28. Private pharmacy<br>29. Public Pharmacy<br>30. From overseas<br>31. Other, specify
	Q3 OBSERVATIONS<br>Note pause, facial expression, verbal responses, etc	
**3a.**	Q2 INTERVIEWER NOTES	

**4.**	**During this pregnancy, how many** months pregnant **were you when you first started taking iron tablet or syrup?**	5. Months [ ][ ]<br>98. Don't know
	Q3 OBSERVATIONS<br>Note pause, facial expression, verbal responses, etc	
**4a.**	Was this question easy or difficult for you to answer?	

Data DENT
ta for Decisions in Nutrition

Addis Continental Institute of Public Health

	Why was it [EASY/ DIFFICULT]?	
**4b.**	IF Q2 RESPOND "YES" TO >1<br><br>Earlier you said that you got [IRON WITH EXTRA VITAMINS OR PRENATAL] how many **months pregnant** were you when you **first started** taking [IRON]?	Q2 yes to 1…………………………SKIP<br>Q2 yes to >1 ………………<br>5. Months [ ][ ]<br>98. Don't know
**4c.**	IF Q2 RESPOND "YES" TO >1<br><br>Earlier you said that you got [IRON OR IRON FOLIC ACID] how many **months pregnant** were you when you **first started** taking [IRON OR IRON FOLIC ACID]?	Q2 yes to 1……………………………….SKIP<br>Q2 yes to >1 ………………<br>4. Months [ ][ ]<br>98. Don't know
**4d.**	IF Q1 RESPOND "YES" TO >1 A-B<br><br>Did you ever take both an [IRON WITH EXTRA VITAMINS OR PRENATAL] tablet or syrup and an [IRON OR IRON FOLIC ACID] tablet on the same day?<br><br>Probe: If yes, please explain. For how much time did you take both irons on the same day?	
**4e.**	Q3 INTERVIEWER NOTES	

**5.**	**In the last week, how many days did you take these iron tablet or syrup?**<br><br>**IF ANSWER IS NOT NUMERIC, PROBE FOR APPROXIMATE NUMBER OF DAYS.**	[ ] NUMBER OF DAYS (0-7)<br>998 = Does not know
	Q4 OBSERVATIONS<br><br>Note pause, facial expression, verbal responses, etc	
**5a.**	Was it easy or difficult is it to remember how many days in the last week you took an iron tablet or syrup?<br><br>Why was it [EASY/ DIFFICULT]?	
**5b**	Can you explain to me the time period the question was talking about?	

**5c.**	You said you took for days, how did you come up with the answer of days?	
**5d.**	You said you took for days, How sure are you of that?	
**5e.**	Q7 INTERVIEWER NOTES	

Text S13: Round 3 Cognitive Interview, RDW, Ethiopia

PHASE II: COGNITIVE INTERVIEW WITH RECENTLY DELIVERED WOMEN

MODULE 1. IDENTIFICATION

IDENTIFICATION	RESPONSE/CODE
NAME OF THE HEALTH FACILITY [Only to fill in if conducted at health facility]	
NAME OF THE SUB-CITY	
NAME OF THE WOREDA	
CURRENTLY PREGNANT OR RECENTLY DELIVERED	1. CURRENTLY PREGNANT<br>2. RECENTLY DELIVERED
NAME OF MOTHER	
MOTHER ID#	
PRIMARY LANGUAGE SPOKEN	
MOBILE NUMBER WHERE RESPONDENT CAN BE REACHED	
**INTERVIEWER INFORMATION**	**CODE**
CODE OF THE INTERVIEWER	
CODE OF THE NOTE-TAKER/SECOND INTERVIEWER	
DATE OF THE INTERVIEW	[ ][ ][ ][ ]<br>DAY<br>MONTH
START TIME (24-HOUR CLOCK)	[ ][ ] : [ ][ ]<br>HR<br>MIN
END TIME (24-HOUR CLOCK)	[ ][ ] : [ ][ ]<br>HR MIN
DURATION (HOUR/MIN)	
**RESULT CODES:**<br>COMPLETE...1<br>PARTIALLY COMPLETE 2	REFUSAL...3<br>ELIGIBLE RESPONDENT AWAY FOR EXTENDED PERIOD 4

MODULE 2. **RECENTLY DELIVERED** RESPONDENT'S BACKGROUND AND PREGNANCY HISTORY

1.	How old are you? Can you tell me what month and year were you born?	Month [ ][ ]<br>98. Don't know month<br>Year [ ][ ][ ][ ]<br>99. Don't know month
2.	How old were you at your last birthday? (response required)	Age in completed years [ ][ ]
3.	What is the highest grade or number of years you have completed at school?	GRADE/YEARS [ ][ ]<br>98. No schooling
4.	Have you lived in Addis Ababa continuously for the last year?	5. Yes → Skip to 5<br>6. No
4a.	If no, when did you come to Addis Ababa?	[ ][ ] [ ][ ]][ ]<br>MONTH YEAR<br>98. Don't know
5.	Now I would like to ask about all the births you have had during your life. Have you ever given birth?<br>**[If no, END INTERVIEW]**	9. Yes<br>10. No
6.	How many total births have you given? (ask for the total number of births even if did not result in a live birth)?	Number of total births [ ][ ]
7.	How old is your youngest child?	13. Weeks [ ][ ]<br>14. Months [ ][ ]<br>15. Year [ ][ ]
8.	Did you see anyone for antenatal care during your most recent pregnancy? **[If no, END INTERVIEW]**	9. No<br>10. Yes<br>98. Don't Know
9.	Where did you receive antenatal care during your most recent pregnancy? Anywhere else?<br><br>RECORD ALL SOURCES. PROBE TO IDENTIFY TYPE OF SOURCE. IF UNABLE TO DETERMINE IF PUBLIC, PRIVATE, OR NGO SECTOR,	49. Home/ Her home<br>50. Other home<br>51. Government hospital<br>52. Government health center<br>53. Other public sector (specify)<br>54. Private hospital<br>55. Private clinic<br>56. Other private medical sector (specify)

	RECORD 'X' AND WRITE THE NAME OF THE PLACE(S).	57. NGO hospital<br>58. NGO clinic<br>59. Other NGO medical sector (specify)<br>60. Other, (specify)
	Whom did you see? Anyone else?<br><br>PROBE TO IDENTIFY EACH TYPE OF PERSON AND RECORD ALL MENTIONED.	29. Doctor<br>30. Nurse/midwife<br>31. Health officer<br>32. Traditional birth attendant<br>33. Health Extension Workers<br>34. Not able to differentiate<br>35. Other (specify)
10.	How many weeks or months pregnant were you when you first received antenatal care for your most recent pregnancy?	11. Weeks [ ][ ]<br>12. Months [ ][ ]<br>98. Don't know
11.	How many times did you received antenatal care during your most recent pregnancy?	Number of times [ ][ ]<br>98. Don't know

MODULE 3. MICRONUTRIENT SUPPLEMENTATION QUESTIONS

INTERVIEWER READ ALOUD: Today I will be asking you questions about products for pregnant women. We would like to know if you understand the questions and how you understand them. The questions we are about to ask you might seem simple or repetitive. But your responses are important to us. There are also no right or wrong answers.

	**3. During your last pregnancy were you given or did you buy any iron tablets or syrups?**<br><br>**DO NOT SHOW VISUAL AID**	YES . . . . 1<br>NO . . . . 2<br>DON'T KNOW 8			
	Q1. OBSERVATIONS<br>Note pause, facial expression, verbal responses, etc.				
**1a.**	Where these questions easy or difficult for you to answer?<br><br>Why was it [EASY / DIFFICULT]?				
**1b.**	Q1 INTERVIEWER NOTES				
	**4. During your last pregnancy which iron tablets or syrups did you take…**<br><br>**A.** IRON WITH MANY VITAMINS OR PRENTATAL?<br>**B.** IRON OR IRON FOLIC ACID?<br><br>SHOW VISUAL AIDS FOR EACH CATEGORY	p. Iron with many vitamins or prenatal	YES<br><br>1<br>1	NO<br><br>2<br>2	DON'T KNOW<br><br>8<br>8

Data DENT
Data for Decisions in Nutrition

Addis Continental Institute of Public Health

INTERVIEW READ ALOUD: Please think about these and similar products; the pictures are just examples.	q. Iron or Iron FOLIC ACID			

	Q2. OBSERVATIONS<br>Note pause, facial expression, verbal responses, etc	
**2a.**	Where these questions easy or difficult for you to answer?<br>Why was it [EASY / DIFFICULT]?	
**2b.**	I asked about "iron with many VITAMINS" in my question. Can you explain to me what "iron with many vitamins" means to you in your own words	
**2c.**	Can you name any of the many vitamins that it contains?	
**2d.**	When I showed you the picture, I said: "Please think about these and similar products; the pictures are just examples." Can you explain what this means to you in your own words	
**2e.**	Q2 INTERVIEWER NOTES	

3	**During your last pregnancy where did you get these iron tablets or syrups? Anywhere else?**<br><br>PROBE TO IDENTIFY THE TYPE OF SOURCE. IF UNABLE TO DETERMINE IF PUBLIC, PRIVATE, OR NGO SECTOR, RECORD 'X' AND WRITE THE NAME OF THE PLACE(S).	32. Government hospitals<br>33. Health centers<br>34. Private hospitals<br>35. NGO/ Charity clinics<br>36. Private pharmacy<br>37. Public Pharmacy<br>38. From overseas<br>39. Other, specify
	Q3 OBSERVATIONS<br><br>Note pause, facial expression, verbal responses, etc	

**3a.**	Q3 INTERVIEWER NOTES	

**4.**	**During your last pregnancy, how many months did you take these iron tablets or syrup?**<br><br>**IF ANSWER IS NOT NUMERIC, PROBE FOR APPROXIMATE NUMBER OF DAYS.**	[ ][ ] MONTHS<br>98 = Does not know
	Q3 OBSERVATIONS<br><br>Note pause, facial expression, verbal responses, etc	
**4a.**	Was it easy or difficult to remember how many months you took a vitamin tablet or syrup?<br><br>Why was it [EASY/DIFFICULT]?	
**4b.**	How did you come up with the answer of months?	
**4c.**	You said months, how sure are you of that?	
**4d.**	IF Q2 RESPOND "YES" TO >1 A-B<br><br>Earlier you said that you got [IRON WITH MANY VITAMINS OR PRENATAL] how many **months** did you take [IRON WITH MANY VITAMINS OR PRENATAL]?	Q2 yes to1……………………………SKIP<br>Q2 yes to >1 ………………….(explain)
**4e.**	IF Q2 RESPOND "YES" TO >1 A-B<br><br>Earlier you said that you got [IRON OR IRON FOLIC ACID] how many **months** did you take [IRON OR IRON FOLIC ACID]?	Q2 yes to 1……………………………SKIP<br>Q2 yes to >1 ………………….(explain)
**4f.**	IF Q1 RESPOND "YES" TO >1 A-B	

Data DENT
Data for Decisions in Nutrition

Addis Continental Institute of Public Health

	Did you ever take both an [IRON WITH MANY VITAMINS OR PRENATAL] tablet or syrup and an [IRON] tablet on the same day?<br><br>Probe: If yes, please explain. For how much time did you take both irons on the same day?	
**4g.**	Q3 INTERVIEWER NOTES	

**5.**	**During your last pregnancy, when you had a supply of these iron tablet or syrup, how many days a WEEK did you usually take them? [Read responses]**	Never………………………………………………1<br>Rarely (1-2 days/wk)……………………...2<br>A few days per week (3-4 d/wk)……..3<br>Most days per week (5-7 d/wk)…….4<br>Everyday......................................5<br>Other(specify)………………………………… 6
	Q6 OBSERVATIONS<br><br>Note pause, facial expression, verbal responses, etc	
**5a.**	Was it easy or difficult to decide what answer to choose?<br><br>Why was it [EASY/ DIFFICULT]?	
**5b.**	How did you come up with the answer of ?	
**5c.**	Women might miss days for many different reasons. Are you thinking about how often you think you should take or how often you actually take?	
**5d.**	Q6 INTERVIEWER NOTES	

Text S14: Round 3 Cognitive Interview Module4 Ethiopia

MODULE 4. VISUAL AID

INTERVIEWER READ ALOUD: Now I would like to ask you some more questions about the pictures we showed you earlier. We can make changes to these pictures so they are more attractive and more helpful. We will ask you questions about how you see these pictures and how we can make them better.

1. PRENATAL: SHOW PRENATAL-3 AS YOU ASK THE FOLLOWING QUESTIONS<br><br>INTERVIEW READ ALOUD: Please think about these and similar products; the pictures are just examples		
**1a**	What products do you recognize in this picture?<br><br>IF "NONE" SKIP TO 2A	
**1b**	Have you ever been given or purchased any of the products in this picture? If yes, which ones?<br><br>RECORD LETTERS	Yes…………………………………………1<br>No………………………………………….2<br><br>If yes, which?
**1c**	Earlier when I showed you this picture (PRENATAL-3) and I asked "were you given or did you buy iron with extra vitamins or prenatal""<br><br>Did you think I was asking whether you took one of these three products in these specific packages or did you think of these and other products similar to these that are not specifically pictured?	
If they recognized UNIMMAP ask the following:		
**1d**	What do you call this product?	
**1e**	Do you or other women use other names for this product?<br><br>Probe: What other name/word do you or other women use? Any other?	
**1f**	Why should a pregnant woman take this product?	

	What does this product contain?	

SHOW IRON -3 AND PRENATAL- 3 AT THE SAME TIME.		
**2a**	Iron comes in many forms. Is there a difference between iron and iron with extra vitamins?<br><br>If yes, what is the difference?	
